# FBXO3 stabilizes USP4 and Twist1 to promote PI3K-mediated breast cancer metastasis

**DOI:** 10.1371/journal.pbio.3002446

**Published:** 2023-12-22

**Authors:** Jing Xu, Rongtian Guo, Nasi Wen, Luping Li, Yong Yi, Jingzhen Chen, Zongyu He, Jian Yang, Zhi-Xiong Jim Xiao, Mengmeng Niu

**Affiliations:** Center of Growth, Metabolism and Aging, Key Laboratory of Bio-Resource and Eco-Environment, Ministry of Education, College of Life Sciences, Sichuan University, Chengdu, China; B.C. Cancer Agency, CANADA

## Abstract

Tumor metastasis is the major cause of breast cancer morbidity and mortality. It has been reported that the F-box protein FBXO3 functions as an E3 ubiquitin ligase in regulating various biological processes, including host autoimmune, antiviral innate immunity, and inflammatory response. However, the role of FBXO3 in tumor metastasis remains elusive. We have previously shown that ΔNp63α is a common inhibitory target in oncogene-induced cell motility and tumor metastasis. In this study, we show that FBXO3 plays a vital role in PI3K-mediated breast cancer metastasis independent of its E3 ligase activity and ΔNp63α in breast cancer cells and in mouse. FBXO3 can bind to and stabilize USP4, leading to Twist1 protein stabilization and increased breast cancer cell migration and tumor metastasis. Mechanistically, FBXO3 disrupts the interaction between USP4 and aspartyl aminopeptidase (DNPEP), thereby protecting USP4 from DNPEP-mediated degradation. Furthermore, p110α^H1047R^ facilitates the phosphorylation and stabilization of FBXO3 in an ERK1-dependent manner. Knockdown of either FBXO3 or USP4 leads to significant inhibition of PI3K-induced breast cancer metastasis. Clinically, elevated expression of p110α/FBXO3/USP4/Twist1 is associated with poor overall survival (OS) and recurrence-free survival (RFS) of breast cancer patients. Taken together, this study reveals that the FBXO3-USP4-Twist1 axis is pivotal in PI3K-mediated breast tumor metastasis and that FBXO3/USP4 may be potential therapeutic targets for breast cancer treatment.

## Abstract 2

Metastasis is the major cause of breast cancer morbidity and mortality. This study reveals a non-canonical function of the E3 ubiquitin ligase FBXO3 in PI3K-induced breast cancer metastasis, showing that FBXO3 binds to and protects USP4 from aspartyl aminopeptidase (DNPEP)-mediated degradation, resulting in Twist1 protein stabilization and increased tumor metastasis.

## Introduction

Breast cancer is the highest diagnosed cancer in recent years and the leading cause of cancer-related deaths among women worldwide [1,2]. Breast cancer exhibits high heterogeneity and high metastatic propensity to distinct organs, including lymph nodes, bone, brain, lung, and liver, which is a leading cause of cancer-related mortality and imposes a huge hurdle for breast cancer treatment [3,4]. It is well documented that activation of a variety of signaling pathways play critical roles in breast cancer metastasis, such as phosphoinositide-3-kinase (PI3K) pathway, mitogen-activated protein kinase (MAPK) pathway, transforming growth factor (TGF)-β/Smad pathway and vascular endothelial growth factor (VEGF) pathway [5–8].

Abnormal activation of the PI3K signaling pathway is pivotal in breast cancer development [9]. PI3Ks are heterodimeric kinases consisting of a catalytic p110 subunit (p110α, p110β, or p110δ) and a regulatory p85 subunit [10]. The hotspot mutation in the PIK3CA gene encoding p110α, H1047R, is the most frequent cancer-specific mutation in breast cancer among others, such as p110α^E542K^ or p110α^E545K^ mutation [11,12]. Activated PI3K continuously transduces oncogenic signals to the downstream pathways, including AKT or ERK pathways [13,14]. Notably, PI3K/AKT signaling induces the phosphorylation of mTORC1, leading to the activation of p70S6K and inactivation of 4EBP1, resulting in increased translation of epithelial–mesenchymal transition (EMT)-related transcription factors, such as Twist, Snail, or Slug, thereby facilitating breast cancer metastasis [15,16]. In addition, our previous study shows that PI3K/AKT signaling can promote cell motility and tumor metastasis by suppressing the expression of ΔNp63α, a member of p53 protein family [17]. PI3K signaling can also cooperate with other signaling pathways, such as TGF-β, NF-κβ, and Wnt/β-catenin, to induce EMT and promote tumor metastasis [15].

EMT plays a critical role in promoting tumor metastasis by enhancing cell mobility, migration, and invasion ability [18]. EMT-related transcription factors, including Twist, Snail, and Zeb family members, can regulate the expression of E-cadherin, Vimentin, N-cadherin, and β-catenin thereby facilitating tumor metastasis [19]. Twist1 is a key EMT-related transcriptional factor through direct transcription repression of E-cadherin and transcription activation of N-cadherin [20]. Various signaling pathways can regulate Twist expression. In response to hypoxia, hypoxia-inducible factor 1α (HIF1α) can up-regulate Twist1 expression to induce EMT and tumor cell dissemination [21]. MAPKs can phosphorylate Twist1 at Ser68 resulting in Twist1 protein stabilization [22]. Our recent work shows that ubiquitin-specific protease 4 (USP4) functions as a deubiquitinase to remove ubiquitin from Twist1, resulting in Twist1 protein stabilization [23].

USP4, as a deubiquitinating enzyme, can remove monoubiquitinated and polyubiquitinated chains from its substrates [24,25]. It plays crucial roles in a variety of cellular and biological processes, including tumorigenesis, distant metastasis, and stemness [26,27]. USP4-mediated regulation of tumor metastasis may be context-dependent. USP4 directly deubiquitylates TGF-β receptor I to induce the phosphorylation of Smad2 and up-regulate matrix metalloproteinase (MMP)-9, thereby facilitating cell migration and invasion of breast cancer cells [28,29]. USP4 can also deubiquitylate PRL-3 to lead to AKT activation, E-cadherin reduction, and distant metastasis [30]. However, USP4 has been reported to target TRAF2 and TRAF6, resulting in the inhibition of TNFα- and IL-1β-induced cell migration [31]. Our recent study shows that USP4 is a cell migration inducer via up-regulation of Twist1 expression [23].

FBXO3 belongs to the F-box protein family. As a substrate-binding subunit of SCF ubiquitin complex, FBXO3 plays a critical role in host autoimmune, antiviral innate immunity, and inflammatory responses [32–34]. FBXO3 binds to and targets the autoimmune regulator (AIRE) protein for degradation to ensure proper expression of tissue-specific antigens [34]. FBXO3 can also regulate the IFN signaling via destabilizing the transcription factors irf3, irf7, and p62 [33,35]. In addition, FBXO3 can activate pro-inflammatory signaling by protecting tumor necrosis factor receptor-associated factors (TRAFs) from FBXL2-mediated degradation [32]. However, the role of FBXO3 in tumor metastasis is largely unclear. In this study, we discovered a noncanonical function of FBXO3 in promoting breast cancer metastasis in an E3 ligase activity-independent manner. We show that the FBXO3-USP4-Twist1 axis plays a causal role in PI3K/ERK-mediated breast cancer cell migration and tumor metastasis.

## Results

### FBXO3 promotes breast cancer cell migration independent of its E3 ligase activity

**Fig 1:**
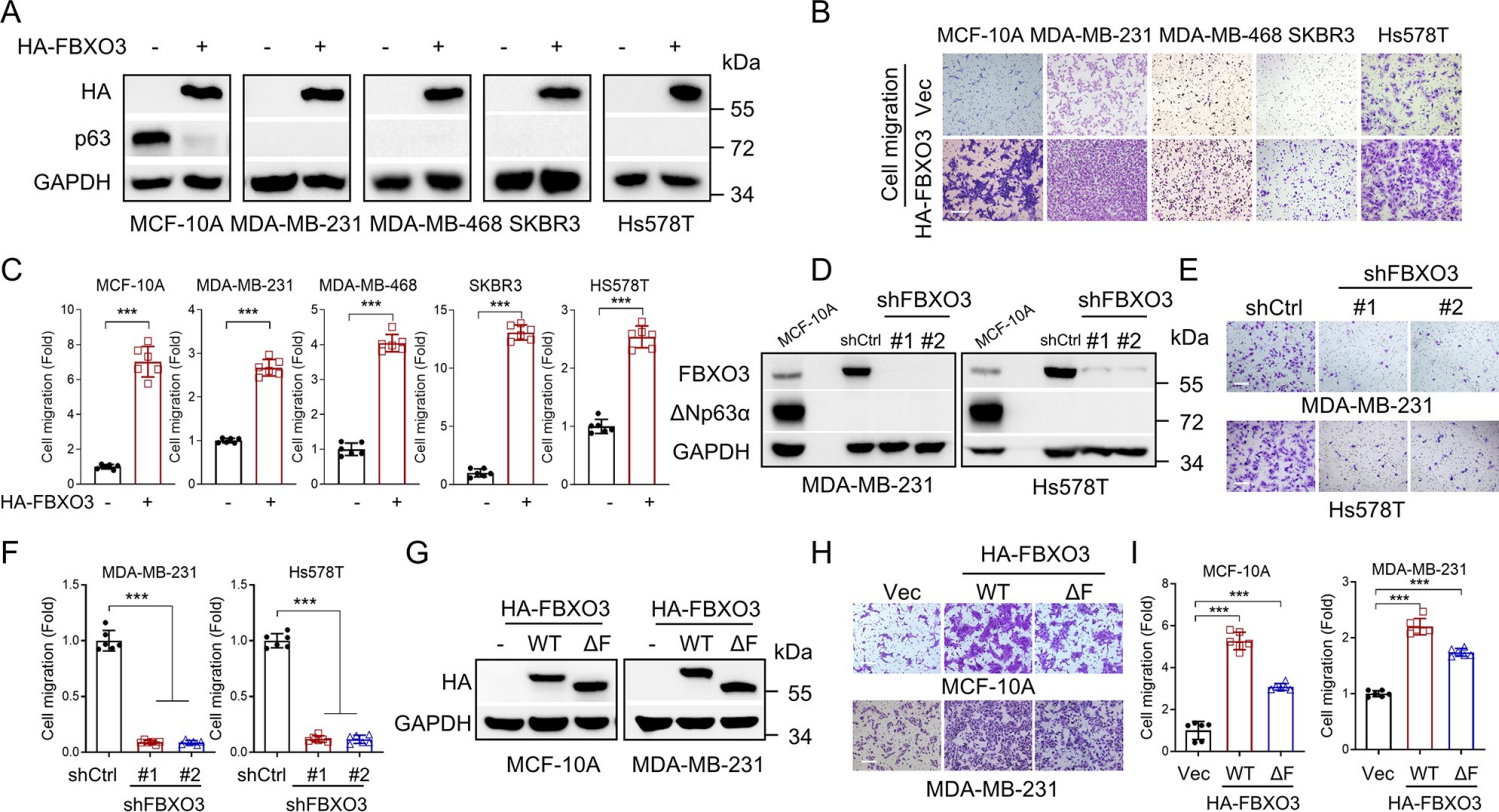
FBXO3 promotes breast cancer cell migration in the ΔNp63α- or E3 ubiquitin ligase-independent manner. (A–C) Human non-transformed mammary epithelial MCF-10A cells with ΔNp63α expression or breast cancer cells without ΔNp63α expression (MDA-MB-231, MDA-MB-468, SKBR3, or Hs578T cells) stably expressing HA-FBXO3 or a vector control (-) were subjected to western blot analyses (A) or transwell assays (B, C). (D–F) MDA-MB-231 or Hs578T cells stably expressing specific shRNA specific for FBXO3 (shFBXO3-#1 or shFBXO3-#2) or GFP (shCtrl) were subjected to western blot analyses (D) or transwell assays (E, F). (G–I) MCF-10A or MDA-MB-231 cells stably expressing HA-FBXO3 WT, HA-FBXO3^ΔF^ (ΔF) or a vector control (-) were subjected to western blot analyses (G) or transwell assays (H, I). Data from 3 independent transwell experiments were presented as means ± SD. ***p < 0.001. Scale bar = 100 μm. The data underlying the graphs shown in the figure can be found in S1 Data. WT, wild-type.

Our previous study demonstrates that ΔNp63α is a common inhibitory target in oncogene-induced cell motility and tumor metastasis [17], we therefore investigated whether FBXO3 can regulate cell migration of breast cancer cells and whether it is implicated in ΔNp63α. Our results showed that while ectopic expression of FBXO3 significantly promoted cell migration in MCF-10A cells, a cell line predominantly expressing ΔNp63α, FBXO3 could also significantly increase cell migration of breast cancer cells in which the expression of ΔNp63α protein were undetectable, including MDA-MB-231, MDA-MB-468, SKBR3, or Hs578T cells (Figs 1A–1C and S1A). The complementary experiments showed that silencing of FBXO3 expression markedly inhibited cell migration in MDA-MB-231 or Hs578T cells, as evidenced by transwell assay and wound-healing assay (Figs 1D–1F and S1B). These results suggest that FBXO3 can promote cell migration in a ΔNp63α-independent manner.

It has been shown that FBXO3 executes its biological functions primarily in its E3 ubiquitin ligase-dependent manner. We then examined whether the E3 ubiquitin ligase activity of FBXO3 is required to impact cell mobility in our experimental system. Notably, ectopic expression of FBXO3^ΔF^ mutant (deletion of F-box domain), which is defective in E3 ubiquitin ligase activity [36], could significantly promote cell migration, albeit less potently compared to the wild-type FBXO3 (Figs 1G–1I, S1C, and S1D). These results suggest that FBXO3 can function to regulate cell migration independent of its E3 ubiquitin ligase activity.

### FBXO3 up-regulates Twist1 expression to promote cell migration and tumor metastasis

**Fig 2:**
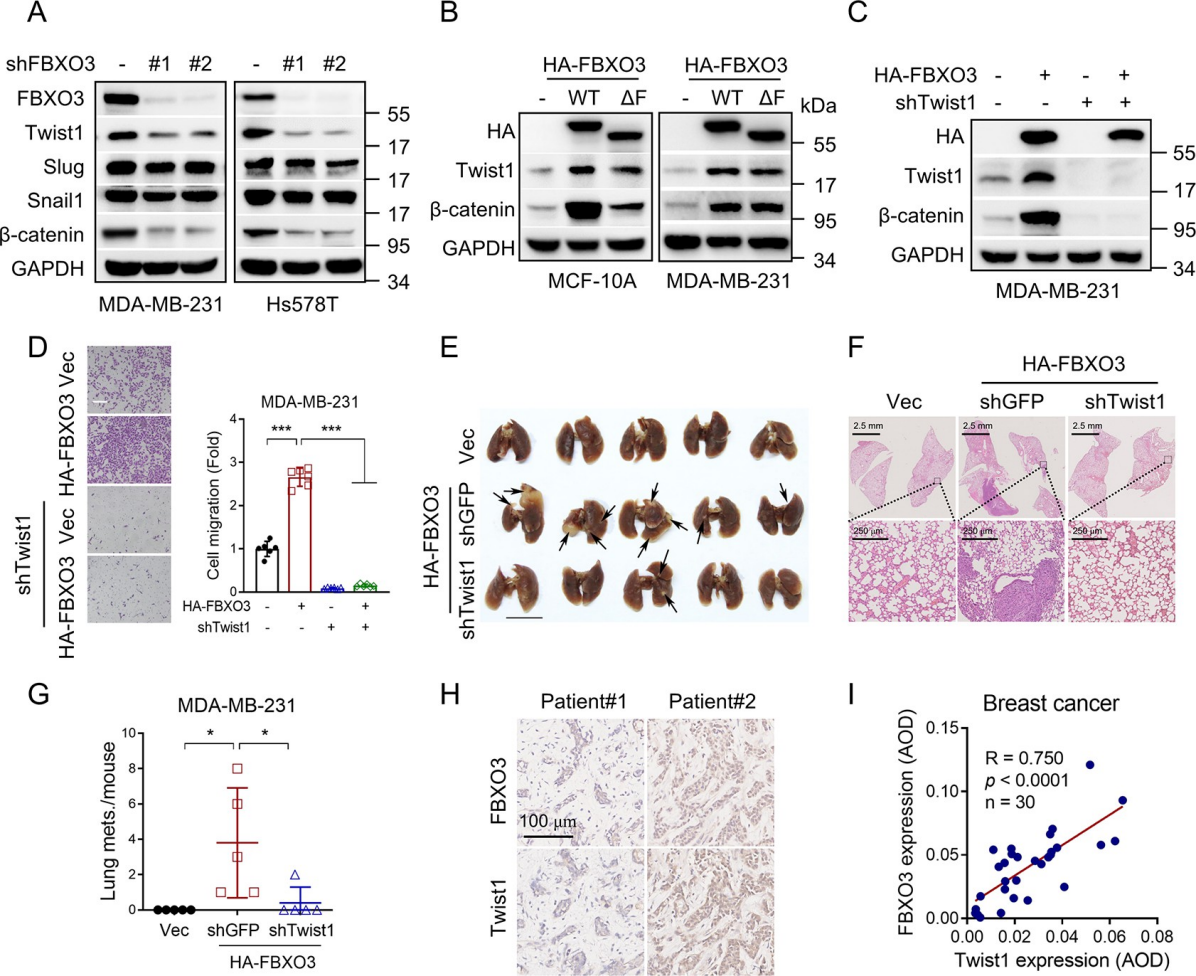
FBXO3 promotes cell migration and tumor metastasis via up-regulation of Twist1 expression. (A) MDA-MB-231 or Hs578T cells stably expressing shFBXO3-#1, shFBXO3-#2, or shGFP (-) were subjected to western blot analyses. (B) MDA-MB-231 or MCF-10A cells stably expressing HA-FBXO3 WT, HA-FBXO3^ΔF^ (ΔF), or a vector control (-) were subjected to western blot analyses. (C) MDA-MB-231 cells stably expressing HA-FBXO3 were infected with a recombinant lentivirus expressing specific shRNA targeting to Twist1 or a vector control (-), and were subjected to western blot analyses. (D) MDA-MB-231 stable cells were subjected to transwell assays. Data from 3 independent experiments were presented as means ± SD. ***p < 0.001. Scale bar = 100 μm. (E–G) MDA-MB-231 stable cells were subjected to tail vein injection in BALB/c nude mice (*n* = 5). Lungs of mice were photographed (E) and stained with HE staining (F), and the number of tumors per mouse was counted (G). Data were presented as means ± SEM. *p < 0.05. (H, I) Consecutive TMA slides derived from human breast cancer specimens (HBreD030PG03, OUTDO, Shanghai, China) were subjected to IHC for expression of FBXO3 and Twist1. IHC staining was quantified by AOD. Representative images of IHC staining and Pearson correlation of FBXO3 and Twist1 expression were shown. Scale bar = 100 μm. The data underlying the graphs shown in the figure can be found in S1 Data. AOD, average optical density; IHC, immunohistochemistry; TMA, tissue microarray; WT, wild-type.

We then investigated the molecular basis by which FBXO3 regulates cell migration independent of ΔNp63α or its E3 ligase activity. Given the critical roles of epithelial–Mesenchymal transition (EMT) transcription factors (including Twist1, Snail1, or Slug) in cell migration, we hypothesized that FBXO3 might affect the expression of EMT transcription factors. Interestingly, silencing of FBXO3 markedly decreased Twist1 expression, while it had little effect on Snail1 and Slug expression in MDA-MB-231 or Hs578T cells (Fig 2A). Notably, similar to wild-type FBXO3, ectopic expression of FBXO3^ΔF^ could also increase the expression of Twist1, accompanied with up-regulated expression of β-catenin, a known downstream target of Twist1 [37] (Fig 2B). These results suggest that Twist1 is a key downstream effector of FBXO3 in regulating cell migration.

We next investigated the causal role of up-regulated Twist1 in FBXO3-induced breast cancer cell migration and tumor metastasis. As shown in Fig 2C and 2D, silencing of Twist1 completely rescued FBXO3-induced cell migration in MDA-MB-231 cells. Furthermore, ectopic expression of FBXO3 significantly increased metastatic nodules in mouse models, which could be effectively rescued by silencing of Twist1 (Fig 2E–2G). We further analyzed the clinical relevance of the FBXO3-Twist1 axis in breast cancer. Immunohistochemical analyses of human breast tissue microarray (TMA) showed a significant positive correlation of the expression of FBXO3 and Twist1 in breast cancer samples (*p* < 0.0001) (Fig 2H and 2I). These results indicate that Twist1 plays a vital role in FBXO3-medicated cancer cell migration and tumor metastasis.

### USP4 is a critical downstream effector of FBXO3 in regulation of Twist1 expression and cell migration

**Fig 3:**
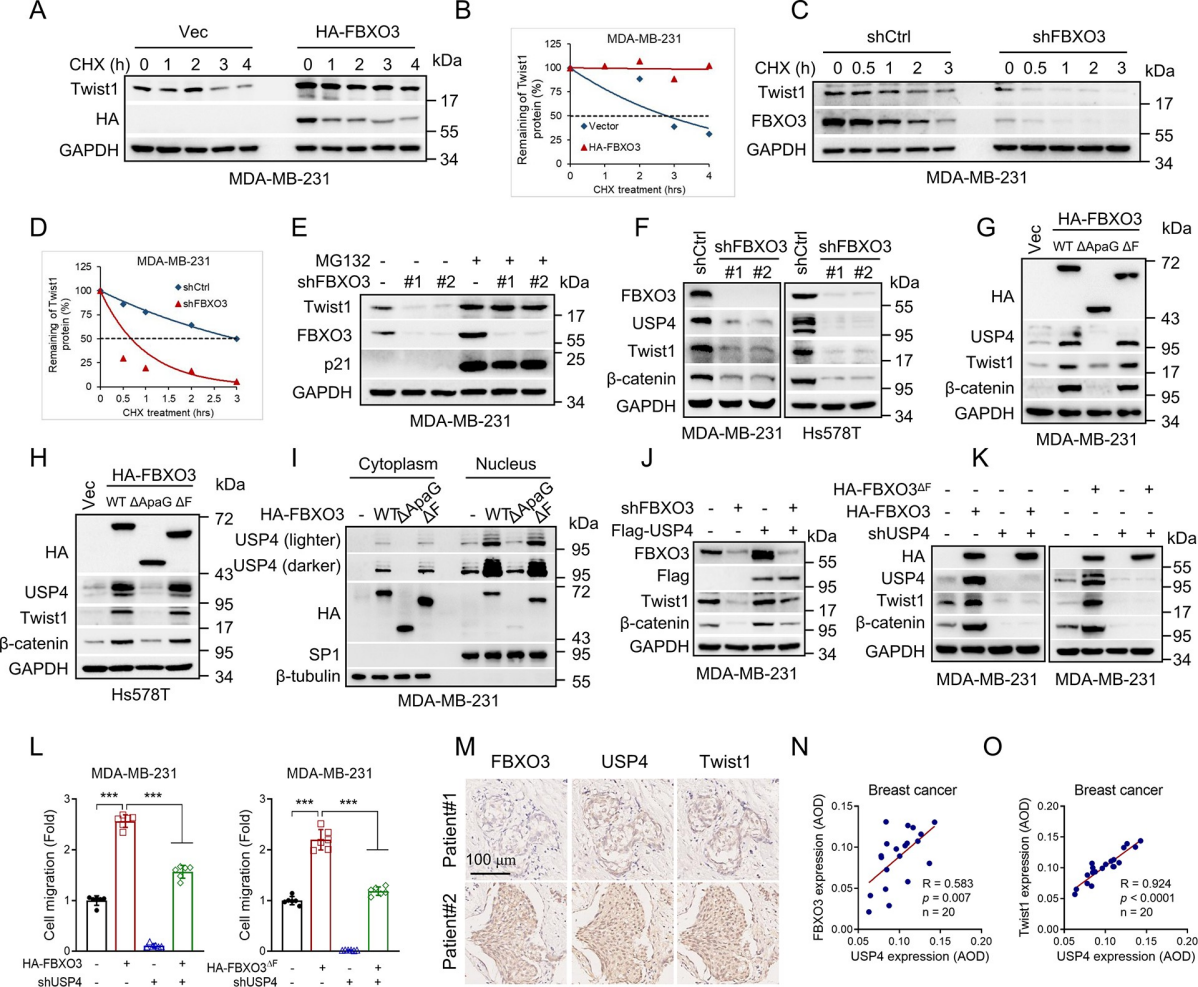
USP4 plays critical roles in FBXO3-induced Twist1 expression and cell migration. (A–D) MDA-MB-231 stable cells were treated with cycloheximide (CHX) for the indicted times (A, C), and the relative Twist1 protein expression levels were quantitated by image J software (B, D). (E) MDA-MB-231 cells stably silencing of FBXO3 were treated with proteasome inhibitor MG132 (10 μm) for 8 h prior to western blot analyses. (F) MDA-MB-231 or Hs578T cells stably expressing shFBXO3-#1, shFBXO3-#2, or shCtrl were subjected to western blot analyses. (G, H) MDA-MB-231 or Hs578T cells stably expressing wild-type HA-FBXO3 (WT), HA-FBXO3^ΔApaG^ (ΔApaG), HA-FBXO3^ΔF^ (ΔF), or a vector control (Vec) were subjected to western blot analyses. (I) MDA-MB-231 cells stably expressing HA-FBXO3, HA-FBXO3^ΔApaG^ (ΔApaG), HA-FBXO3^ΔF^ (ΔF), or a vector control (-) were subjected to the nuclear separation assays. (J, K) MDA-MB-231 stable cells were subjected to western blot analyses. (L) MDA-MB-231 stable cells were subjected to transwell assays. Data from 3 independent experiments were presented as means ± SD. ***p < 0.001. (M–O) Consecutive TMA slides derived from human breast cancer specimens (HBreD020PG01, OUTDO, Shanghai, China) were subjected to IHC for expression of USP4, FBXO3, and Twist1. Representative images of IHC staining (M) and Pearson correlation of USP4 and FBXO3/Twist1 expression were shown (N, O). Scale bar = 100 μm. The data underlying the graphs shown in the figure can be found in S1 Data. IHC, immunohistochemistry; TMA, tissue microarray; WT, wild-type.

We further explored the molecular basis by which FBXO3 regulates Twist1 expression. As shown in S2A Fig, knockdown of FBXO3 did not significantly impact on steady-state Twist1 mRNA levels. However, knockdown of FBXO3 markedly shortened the half-life of Twist1 protein, whereas ectopic expression of HA-FBXO3 prolonged the half-life of Twist1 protein in MDA-MB-231 or MCF-10A cells (Figs 3A–3D, S2B, and S2C). Notably, FBXO3 knockdown-mediated down-regulation of Twist1 could be rescued by proteasome inhibitor (MG132) (Fig 3E), indicating that FBXO3 can prevent Twist1 for proteasome-mediated degradation. We have previously shown that USP4 can deubiquitinate and stabilize Twist1 protein [23], so we then asked whether USP4 is involved in FBXO3-induced Twist1 stabilization. As shown in Fig 3F, silencing of FBXO3 markedly reduced USP4, concomitant with down-regulation of Twist1 expression in MDA-MB-231 and Hs578T cells. In complementary experiments, ectopic expression of either wild-type FBXO3 or FBXO3^ΔF^, led to the up-regulation of UPS4 and Twist1 protein expression (Fig 3G and 3H). Notably, FBXO3 protein contains an ApaG domain in addition to F-box domain [32]. Unlike FBXO3^ΔF^, FBXO3^ΔApaG^ mutant lacking the ApaG domain failed to regulate USP4 and Twist1 (Fig 3G and 3H). In keeping with this observation, ectopic expression of FBXO3 or FBXO3^ΔF^, but not FBXO3^ΔApaG^, led to increased USP4 expression in both nucleus and cytoplasm (Fig 3I). These results indicate that FBXO3 increases Twist1 and USP4 protein expression in an E3 ligase activity-independent manner, in which the FBXO3-ApaG domain is indispensable.

To investigate the causal effects of USP4 on FBXO3-induced Twist1 expression and cell migration, we performed rescuing experiments. As shown in Fig 3J, restoration of USP4 effectively reversed FBXO3 knockdown-mediated down-regulation of Twist1 and β-catenin. Consistently, wild-type FBXO3- or FBXO3^ΔF^- mediated the up-regulation of Twist1 and β-catenin could be rescued by silencing of USP4 in MDA-MB-231 or Hs578T cells (Figs 3K and S2D). Furthermore, silencing of USP4 significantly rescued FBXO3- or FBXO3^ΔF^-induced cell migration in MDA-MB-231 cells (Figs 3L, S2E, and S2F). Together, these results indicate that the USP4 is a critical downstream effector of FBXO3 in regulation of Twist1 expression and cell migration. Notably, IHC analyses of human breast TMA showed that USP4 was positively correlated with the expression of FBXO3 or Twist1 in breast cancer samples (Fig 3M–3O).

### FBXO3 binds to USP4 and disrupts the interaction between USP4 and DNPEP, leading to stabilization of USP4 and Twist1

**Fig 4:**
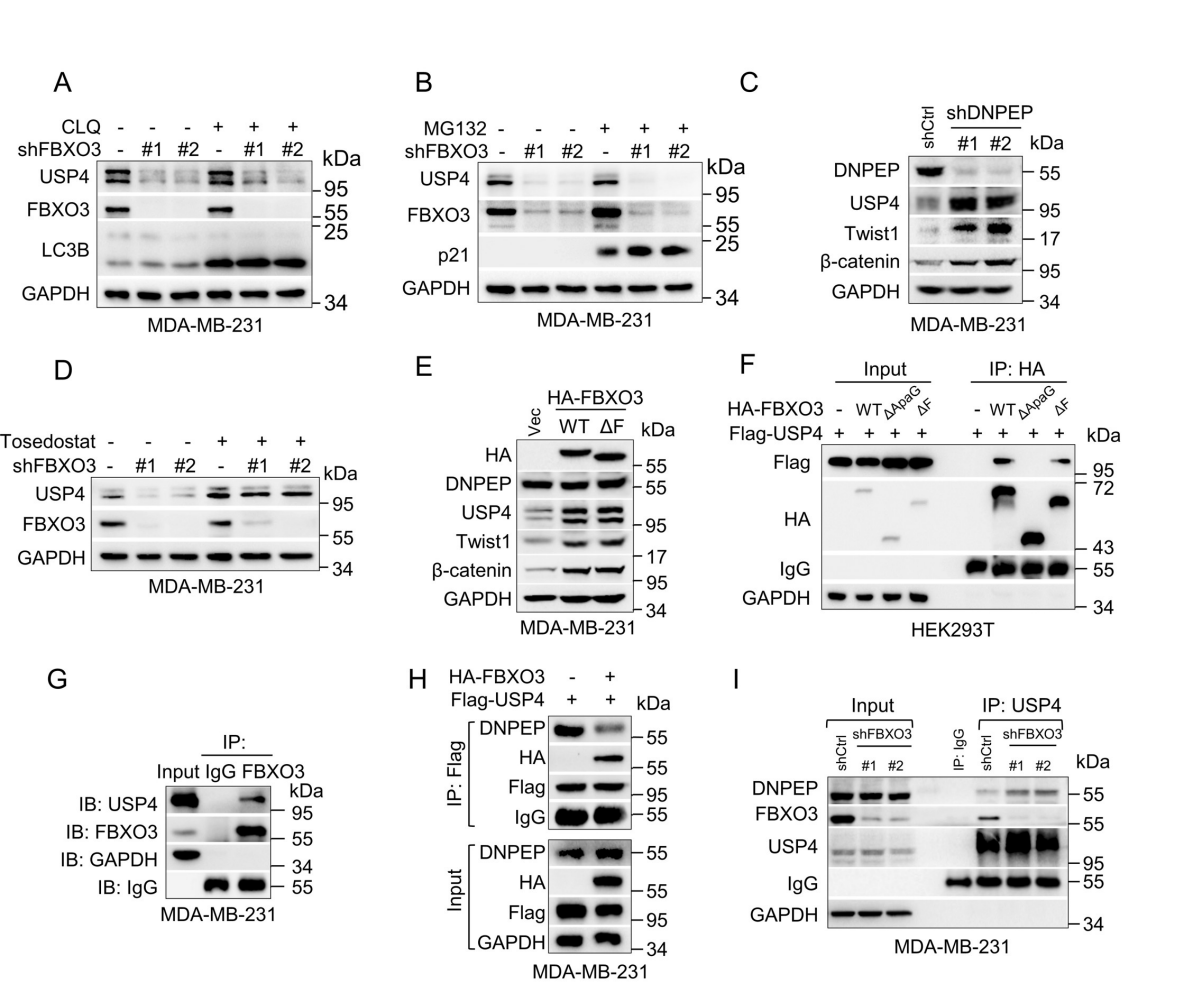
FBXO3 binds USP4 to disrupt the interaction between USP4 and DNPEP, leading to stabilization of USP4 and Twist1. (A, B) MDA-MB-231 cells stably silencing of FBXO3 were treated with proteasome inhibitor MG132 (10 μm) for 8 h or lysosomal inhibitor chloroquine (CLQ, 45 μm) for 36 h prior to western blot analyses. (C) MDA-MB-231 cells stably silencing of DNPEP were subjected to western blot analyses. (D) MDA-MB-231 cells stably silencing of FBXO3 were treated with aminopeptidase inhibitor Tosedostat (0.5 μm) for 16 h prior to western blot analyses. (E) MDA-MB-231 cells stably expressing HA-FBXO3, HA-FBXO3^ΔF^, or a vector control (Vec) were subjected to western blot analyses. (F) HEK293T cells were co-transfected with expressing plasmids encoding Flag-USP4, HA-FBXO3, HA-FBXO3^ΔApaG^, HA-FBXO3^ΔF^, or a vector control (-) for 36 h, and cells were then treated with 0.5 μm Tosedostat for 16 h prior to IP-western assays. (G) MDA-MB-231 cells were subjected to IP-western assays. (H) MDA-MB-231 cells stably expressing Flag-USP4 were infected with a recombinant lentivirus expressing HA-FBXO3 or a vector control (-). Cells were then treated with 0.5 μm Tosedostat for 16 h prior to IP-western assays. (I) MDA-MB-231 cells stably silencing of FBXO3 were treated with 0.5 μm Tosedostat for 16 h and were then subjected to IP-western assays. USP4, ubiquitin-specific protease 4.

We further investigated the molecular basis by which FBXO3 up-regulates USP4 expression. As shown in S3A and S3B Fig, ectopic expression of FBXO3 or silencing of FBXO3 had little effect on the steady-state USP4 mRNA levels. Furthermore, knockdown of FBXO3-induced down-regulation of USP4 could not be rescued by either lysosome inhibitor (chloroquine) or proteasome inhibitor (MG132) (Fig 4A and 4B), suggesting that knockdown of FBXO3-induced down-regulation of USP4 is unlikely to be at the transcriptional levels or at the levels of the proteasome-/lysosome-mediated protein stability.

It has been reported that aspartyl aminopeptidase (DNPEP) can bind to and degrade USP4 [38]. Our results showed that knockdown of DNPEP indeed prolonged the half-life of USP4 protein and increased USP4 and Twist1 expression (Figs 4C, S3C, and S3D). We therefore examined whether DNPEP is involved in FBXO3-induced USP4 up-regulation. As shown in Fig 4D, knockdown of FBXO3-mediated down-regulation of USP4 could be effectively rescued by treatment of Tosedostat, an aminopeptidase inhibitor, suggesting that FBXO3 may stabilize USP4 protein by preventing DNPEP-induced USP4 degradation. Notably, ectopic expression of FBXO3 had little effect on DNPEP expression (Fig 4E). Thus, we suspected that FBXO3 may act to block DNPEP interaction with USP4. Indeed, the co-immunoprecipitation assays showed that wild-type FBXO3 or FBXO3^ΔF^, but not FBXO3^ΔApaG^, could form stable complexes with USP4 (Fig 4F and 4G). Importantly, the elevation of FBXO3 expression significantly reduced complex formation between USP4 and DNPEP, whereas silencing of FBXO3 increased endogenous DNPEP binding to USP4 in MDA-MB-231 cells (Fig 4H and 4I). Together, these results indicate that FBXO3 binds USP4 to interfere with the interaction between USP4 and DNPEP, leading to stabilization of the USP4 protein.

### Activation of PI3K promotes phosphorylation and stabilization of FBXO3 in an ERK1-dependent manner

**Fig 5:**
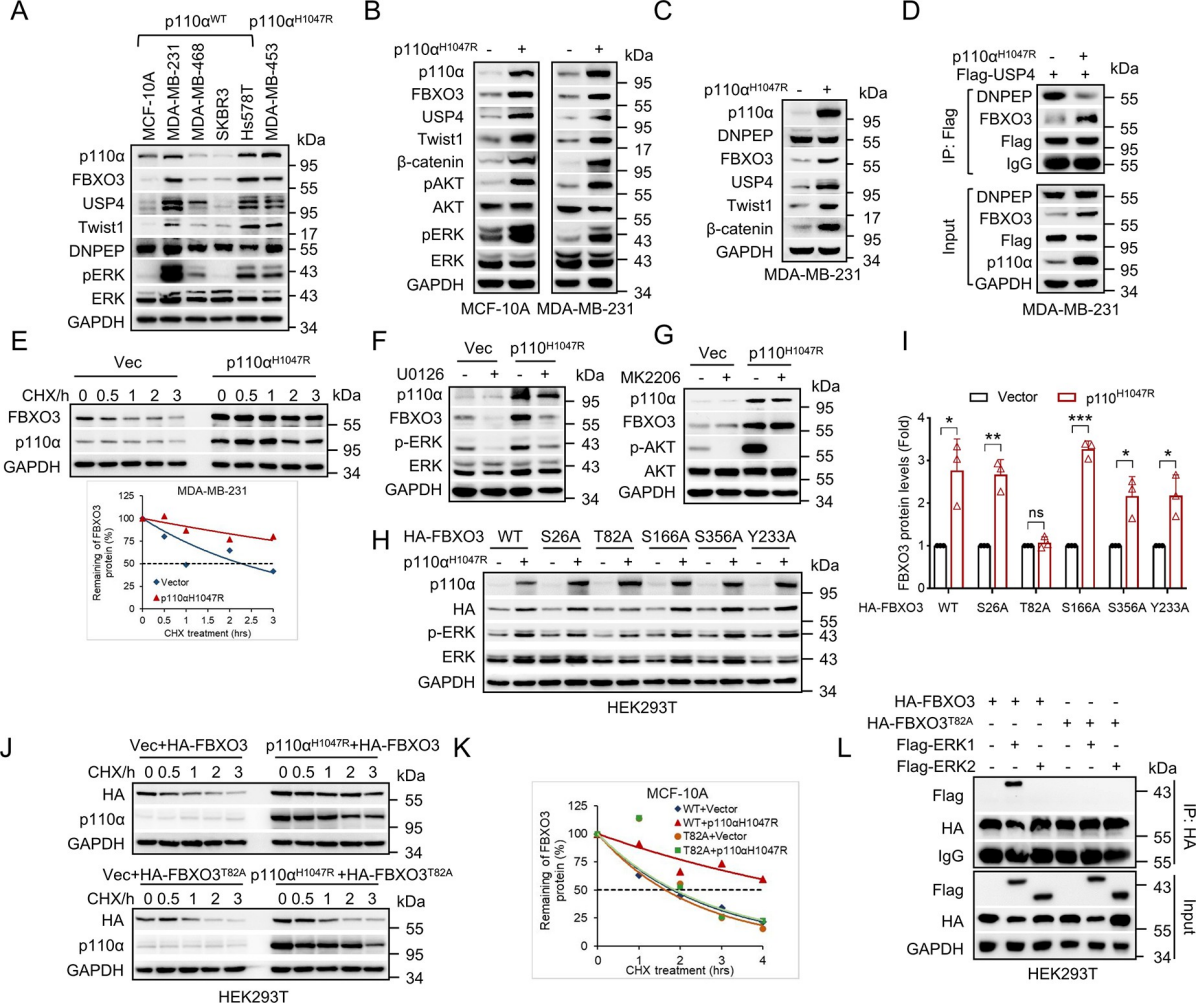
Activation of PI3K facilitates phosphorylation and stabilization of FBXO3 in an ERK1-dependent manner. (A) MCF-10A, MDA-MB-231, MDA-MB-468, SKBR3, Hs578T, or MDA-MB-453 (with p110α^H1047R^) cells were subjected to western blot analyses. (B) MCF-10A or MDA-MB-231 stable cells were subjected to western blot analyses. (C) MDA-MB-231 cells stably expressing p110α^H1047R^ or a vector control (-) were subjected to western blot analyses. (D) MDA-MB-231 stable cells were treated with 0.5 μm Tosedostat for 16 h and were then subjected to IP-western assays. (E) MDA-MB-231 cells stably expressing p110α^H1047R^ or a vector control (Vec) were treated with cycloheximide (CHX) for the indicted times, and the relative FBXO3 protein expression levels were quantitated by image J software. (F, G) MCF-10A stable cells were treated with 10 μm U0126 for 12 h or 8 μm MK2206 for 8 h prior to western blot analyses. (H, I) HEK293T cells were co-transfected with p110α^H1047R^ and either HA-FBXO3 WT or HA-FBXO3 mutant (S26A, T82A, S166A, S356A, or Y233A) expressing plasmids for 36 h, and then cells were subjected to western blot analyses. (J, K) HEK293T cells were co-transfected with p110α^H1047R^ and either HA-FBXO3 wild-type or HA-FBXO3^T82A^ mutant expressing plasmids, and were then treated with cycloheximide (CHX) for the indicted times prior to western blot analyses (J). The relative FBXO3 protein expression levels were quantitated by image J software (K). (L) HEK293T cells were co-transfected with the indicated expressing plasmids, and cells were then subjected to IP-western assays. The data underlying the graphs shown in the figure can be found in S1 Data. WT, wild-type.

We then explored the upstream signaling in the regulation of the FBXO3-USP4-Twist1 axis. Given the critical role of PI3K signaling in breast cancer metastasis [39], we evaluated the relationship between PI3K signaling and FBXO3. As shown in Figs 5A and S4A, activation of PI3K signaling (including both expression levels of the PI3K catalytic subunit p110α or the up-regulated ERK phosphorylation) was positively correlated with cell migration capacity as well as FBXO3/USP4/Twist1 protein levels in breast cancer cell lines examined. Notably, while ectopic expression of either p110α^H1047R^, p110α^E542K^, or p110α^E545K^, all of which are constitutive activation mutants of p110α associated with breast cancer [12,40,41], let to activation of AKT and ERK, it significantly increased the expression of FBXO3, accompanied with the up-regulated USP4, Twist1, and β-catenin protein expression (Figs 5B, S4B, and S4C). In addition, p110α^H1047R^ drastically promoted FBXO3 and USP4 in both nucleus and cytoplasm (S4D Fig). Furthermore, while p110α^H1047R^ had little effect on DNPEP expression, it significantly up-regulated and prolonged the half-life of FBXO3, leading to disruption of USP4 and DNPEP complex formation (Figs 5C–5E and S4E).

Further investigation showed that inhibition of ERK by a specific MEK inhibitor U0126, but not AKT inhibitor MK2206, could effectively rescue p110α^H1047R^-induced FBXO3 expression (Fig 5F and 5G), which promoted us to examine the effects of p110α^H1047R^ on ERK-mediated phosphorylation of FBXO3. In addition to the previously reported Erk phosphorylation sites on FBXO3, including Ser166 and Tyr233 (phosphoSitePlus PTM Database), the computer aided analyses (GPS 5.0 Software) reveal 3 potential ERK phosphorylation sites (Ser26, Thr82, and Ser356) on FBXO3. As shown in Figs 5H–5K and S4F–S4G, while p110α^H1047R^ led to protein stabilization of wild-type FBXO3 or other 4 FBXO3 mutant proteins bearing a point mutation, it failed to stabilize FBXO3^T82A^ mutant protein. Furthermore, co-immunoprecipitation assays showed that ERK1, but not ERK2, formed stable protein complexes with FBXO3 (Fig 5L). Taken together, these results indicate that p110α^H1047R^ can phosphorylate Thr82 of FBXO3 leading to its stabilization in an ERK1-dependent manner.

### The FBXO3-USP4-Twist1 axis plays a critical role in p110α^H1047R^-induced cell migration and tumor metastasis

**Fig 6:**
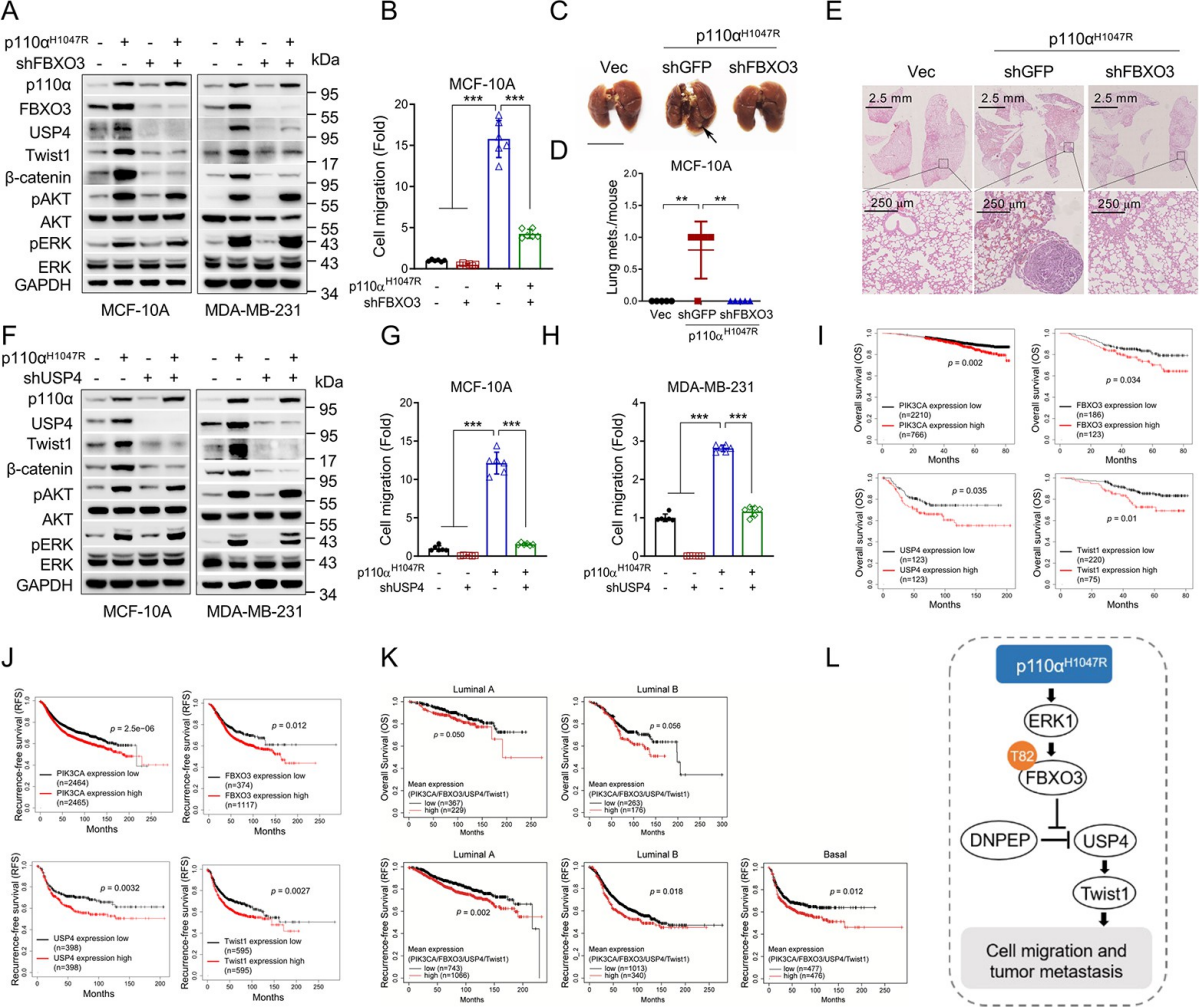
The FBXO3-USP4-Twist1 axis plays a critical role in p110α^H1047R^-induced cell migration and tumor metastasis. (A) MCF-10A and MDA-MB-231 cells stably expressing p110α^H1047R^ were infected with a recombinant lentivirus expressing specific shRNA targeting to FBXO3 or GFP (-). Cells were then subjected to western blot analyses. (B) MCF-10A stable cells were subjected to transwell analyses. Data from 3 independent experiments were presented as means ± SD. ***p < 0.001. (C–E) MCF-10A stable cells were subjected to tail vein injection in BALB/c nude mice (*n* = 5). Representative photos of the lung were presented and the observable nodules per mouse in the lung surface were presented (C, D) (scale bar = 1 cm). Paraffin-embedded lungs were sectioned and subjected to HE staining (E). Data were presented as means ± SEM. **p < 0.01. (F–H) MCF-10A and MDA-MB-231 stable cells were subjected to western blot analyses (F) or transwell analyses (G, H). Data from 3 independent experiments were presented as means ± SD. ***p < 0.001. (I, J) Kaplan–Meier plots of OS and progression-free survival (RFS) of human breast cancer patients were stratified by the PIK3CA, FBXO3, USP4, or Twist1 mRNA expression levels in the patient tumor samples. (K) Kaplan–Meier plots of OS and progression-free survival (RFS) of human breast cancer patients were stratified by the mean expression of a set of 4 genes (PIK3CA, FBXO3, USP4, and Twist1) in the patient tumor samples. (L) A working model depicts the regulatory function of FBXO3 on breast cancer metastasis and the role of the FBXO3-USP4-Twist1 axis in PI3K-induced tumor metastasis. The data underlying the graphs shown in the figure can be found in S1 Data. OS, overall survival; RFS, recurrence-free survival; USP4, ubiquitin-specific protease 4.

To investigate the causative role of FBXO3 in p110α^H1047R^-induced breast cancer cell migration and tumor metastasis, we performed rescue experiments. As shown in Figs 6A and 6B and S5A and S5B, silencing FBXO3 significantly rescued the p110α^H1047R^-induced up-regulation of Twist1, USP4, and β-catenin as well as revised the p110α^H1047R^-mediated increase of cell migration. Importantly, the p110α^H1047R^-induced tumor metastasis could also be rescued by silencing of FBXO3 in a mouse metastasis model (Fig 6C–6E). Furthermore, silencing of USP4 rescued p110α^H1047R^-mediated up-regulation of Twist1 and β-catenin, accompanied with reduced cell migration (Figs 6F–6H, S5C, and S5D). Moreover, Kaplan–Meier survival analysis showed that high expression of PIK3CA, FBXO3, USP4, or Twist1 expression was associated with poor overall survival (OS) and recurrence-free survival (RFS) (Fig 6I and 6J). Further analysis of the prognostic value of PI3KCA/FBXO3/USP4/Twist1 pathway showed that high-level expression of the gene set (PI3KCA; FBXO3; USP4; Twist1) was associated with poor OS and RFS (Fig 6K). Together, these results demonstrate that the FBXO3-USP4-Twist1 axis is critically important in p110α^H1047R^-induced cell migration and tumor metastasis, and that elevated expression of PIK3CA/FBXO3/USP4/Twist1 is associated with poor clinical outcomes of breast cancer patients.

## Discussion

The E3 ubiquitin ligase FBXO3 plays important roles in regulation of host autoimmune, antiviral innate immunity, and inflammatory responses [32–34]. FBXO3 protein consists of an F-box domain (aa 10–56) and an ApaG (Adenine tetraphosphate adenine G) domain (aa 278–408) [36,42]. The F-box domain of FBXO3 is indispensable for its E3 ubiquitin ligase activity by interacting with SKP1 to form the SCF complex (Skp1–Cullin–F-box-protein) [36]. The function of FBXO3-ApaG domain is much less unclear, yet it has been reported important in binding to and degrading FBXL2 [32]. In this study, we discovered a noncanonical function of FBXO3 in tumor metastasis. FBXO3 functions as a USP4 binding partner to protect USP4 from degradation, which in turn leads to stabilization of Twist1, thereby promoting cell migration and tumor metastasis.

Importantly, we found that the E3 ubiquitin ligase activity of FBXO3 is not required in the FBXO3-USP4-Twist1 axis. Rather, the ApaG domain of FBXO3 is indispensable for FBXO3 interaction with USP4. Notably, FBXO3 can affect cell migration in the MDA-MB-231 cells which do not express detectable ΔNp63α expression, indicating that FBXO3 can impact cell migration independent of ΔNp63α, a common inhibitory target in oncogene-induced tumor metastasis [17]. However, FBXO3^ΔF^ lacking of the E3 ligase activity can also induce cell migration but less potently than that of wild-type FBXO3 in MDA-MB-231 cells, implying that FBXO3 can impact cell migration independent of its E3 ubiquitin ligase activity and that FBXO3 may regulate other substrates to influence cell migration. For instance, FBXO3 may influence cell migration through FBXL2-mediated EGFR protein abundance, in which FBXL2 has been shown as a substrate of FBXO3 [32,43].

USP4 is critically involved in tumor growth and metastasis. USP4 protein stability is critically regulated. AKT can facilitate USP4 phosphorylation and stabilization [29]. USP4 can also be self-deubiquitylated to promote homologous recombination [44]. Notably, aspartyl aminopeptidase (DNPEP) can bind to, hydrolyze, and destabilize USP4 [38]. In this study, we demonstrate that USP4 is a key downstream effector in PI3K-induced breast cancer metastasis. Mechanistically, we found that the ApaG domain of FBXO3 binds to USP4 to prevent USP4 interaction with DNPEP thereby leading to USP4 protein stabilization. It is plausible that FBXO3 and DNPEP may compete to bind USP4, a notion that deserves further investigation.

FBXO3 can function as an E3 ubiquitin ligase to regulate various physiological and biochemical processes, yet how FBXO3 is regulated is elusive. It has been reported that lipopolysaccharide (LPS) exposure can up-regulate FBXO3 expression [45,46]. In addition, MiR-142-3p can reduce FBXO3 mRNA levels to attenuate oxygen/glucose deprivation-induced injury [47]. In this study, we demonstrate that activation of the PI3K signaling leads to FBXO3 phosphorylation at Thr82 and protein stabilization in an ERK1-dependent manner. Taken together, this study reveals the pivotal role of the FBXO3-USP4-Twist1 axis in PI3K-mediated breast tumor metastasis, adding another layer of regulation in PI3K-induced tumor metastasis. Thus, targeting FBXO3/USP4 may represent a new therapeutic strategy for breast cancer treatment.

## Materials and methods

### Ethics statement

All animal experiments in this study were performed in accordance with the institutional ethical guidelines for animal experiments and were approved by the Animal Ethics Committee of the College of Life Sciences, Sichuan University (Animal ethical license number: SCU220713001).

### Cell culture and drug treatment

Human embryonic kidney HEK293T cells, human breast cancer MDA-MB-231, MDA-MB-468 or Hs578T cells were purchased from ATCC and were cultured in DMEM (GIBCO, Rockville, Maryland, United States of America), supplemented with 10% fetal bovine serum (FBS; Hyclone, Logan, Utah, USA), 100 units/mL penicillin (GIBCO) and 100 μg/mL streptomycin (GIBCO). Among them, the medium of Hs578T cells was supplemented with 10 μg/mL insulin (Sigma, St. Louis, Missouri, USA). Human breast cancer MDA-MB-453 or SKBR3 cells were purchased from ATCC and were cultured in Leibovitz’s L-15 (GIBCO) medium or McCoy’s 5A medium (Basalmedia Technologies, Shanghai, China), respectively, both of which were supplemented with 10% FBS, 100 units/mL penicillin (GIBCO) and 100 μg/mL streptomycin (GIBCO). Human non-transformed mammary epithelial MCF-10A cells were purchased from ATCC and were grown in F12 (Invitrogen, Carlsbad, California, USA), supplemented with 5% horse serum, 100 ng/mL cholera toxin (Sigma, St. Louis, Missouri, USA), 10 μg/mL insulin (Sigma, St. Louis, Missouri, USA), 20 ng/mL epidermal growth factor (Invitrogen, Carlsbad, California, USA), and 500 ng/mL (95%) hydrocortisone (Sigma). All cells were incubated at 37°C and 5% CO_2_. Cells were treated with 10 μm U0126 (Selleck Chemicals, Houston, Texas, USA) for 12 h, 8 μm MK2206 (Selleck) for 8 h, 10 μm MG132 (Selleck) for 8 h, 45 μm chloroquine (Selleck) for 36 h or 0.5 μm aminopeptidase inhibitor Tosedostat (MedChemExpress (MCE), Shanghai, China) for 16 h at 70% to 85% confluence. Cells were treated with 20 or 50 μg/mL the protein synthesis inhibitor cycloheximide (CHX) (Sigma) and total protein was extracted at various time points. The expression of proteins was quantified by image J software.

### Plasmid DNA transfection, lentiviral infection, and RNA interference

Cells were transfected utilizing Lipofectamine 2000 (Invitrogen, Carlsbad, California, USA) according to the manufacturer’s protocols. Lentivirus were generated by transfecting HEK293T cells at 80% to 90% confluence with pMD2.G and psPAX2 packaging plasmids and one of lentiviral expression plasmids. Virus was collected at 72 h post-transfection and used for cell infection at 30% confluence with 10 μg/mL polybrene (Sigma). Lentivirus-mediated FBXO3 shRNAs #1 (5′-CCTGGGTTCTATGTGACACTA-3′), #2 (5′-AGGAAGATACATTGACCATTA-3′); USP4 shRNA (5′-CCCAACTGTAAGAAGCATCAA-3′); Twist1 shRNA (5′-GCTGAGCAAGATTCAGACC-3′) and control (5′-TGCGTTGCTAGTACCAAC-3′) were cloned into the pLKO.1-puro (Shanghai, GeneChem). Depletion efficiency was evaluated by western blot analysis.

### Co-immunoprecipitation and western blot analysis

For co-immunoprecipitation assays, cell lysates were centrifuged and the supernatant was incubated with an indicated antibody (anti-FBXO3 (SC-514625) or anti-USP4 (SC-376000) purchased from Santa Cruz Biotechnology) prior to incubation with Protein G Plus/Protein A Agarose beads (Calbiochem) or was incubated with FLAG/HA M2-conjugated agarose beads (Sigma Aldrich) at 4°C overnight. The beads were washed 3 times with cold wash buffer and the precipitated proteins were further analyzed by western blot analyses. For total protein isolation, cells were lysed in EBC250 lysis buffer (250 mM NaCl, 50 mM Tris-HCl 8.0, 0.5% Nonidet P-40, and supplemented with 0.5 mM Na_3_VO_4_, 50 mM NaF, 0.2 mM PMSF, 20 μg/mL aprotinin, and 10 μg/mL leupeptin). Western blotting analyses were performed as described [48]. Anti-GAPDH antibody (AB0036) was purchased from Abways (Shanghai, China). Anti-β-catenin (610154) antibody was purchased from BD Transduction (Lexington, Kentucky, USA). Anti-p110α (4249), anti-ERK (9102), anti-pERK (9101), anti-AKT (9272), anti-pAKT (4058), anti-HA (3724), anti-Snail (3895), and anti-Slug (9585) antibodies were purchased from Cell Signaling Technology (CST). Anti-Twist1 (AF5224) and anti-DNPEP (DF12954) antibodies were purchased from Affinity Biosciences. Anti-FBXO3 (SC-514625) antibody was purchased from Santa Cruz Biotechnology. Anti-USP4 (612819) was purchased from zenbioscience (Chengdu, China). Goat anti-rabbit IgG HRP (SC-2004) and anti-mouse (SC-2005) (Thermo Fisher) were used as secondary antibodies.

### RNA isolation, RT-PCR, and real-time PCR

Total RNA was isolated using RNeasy plus Mini Kit (macherey-nagel) (Düren, Germany) as per the manufacturer’s instruction; 1 to 2 μg of total RNA was reverse-transcribed with Superscript III reverse transcriptase (Invitrogen) and Oligo (dT) (Invitrogen). Real-time quantification of USP4 and Twist1 was performed using power SYBR Green PCR Master Mix (Applied Biosystems, Foster City, California, USA). GAPDH was used as the endogenous control. The following primers were used: USP4 (F: 5′-CTTATTGACAGCCGGTGGTT-3′, R: 5′- GTTTATTCCACGCCTCGGTA-3′); Twist1 (F: 5′-GTCCGCAGTCTTACGAGGAG-3′, R: 5′-GCTTGAGGGTCTGAATCTTGCT-3′); GAPDH (F: 5′-TGGACTCCACGACGTACTCA-3′, R: 5′-AATCCCATCACCATCTTCCA-3′). Each measurement was performed in CFX-96 Real time PCR System (Bio-Rad, Saint-Laurent, QC, Canada) with Bio-Rad SsoFast EvaGreen Supermix (Bio-Rad) and analyzed using Bio-Rad Software. Gene expression was determined using the threshold cycles (ΔΔCT) method. Data are always presented as mean ± SD, and technical triplicates were performed in all cases.

### Wound-healing and transwell assay

For wound-healing assays, cells were seeded into 6-well plates and grown to 90% to 100% confluency in growth media, and then wounded with a 1 milliliter pipette tip. Cells were washed twice with PBS and incubated in serum-free media. At indicated time intervals, migrated cells into the wound were photographed under a light microscope (Nikon Eclipse Ti-S/L 100). Transwell assays for migration were performed in transwell inserts with a 6.5-mm, 8.0-μm-pore polycarbonate membrane (BD Biosciences, San Jose, California, USA). Briefly, cells were suspended in serum-free media and seeded into the inner chamber (MCF-10A, 5 × 10^4^ cells per chamber; MDA-MB-231, 1 × 10^4^ cells per chamber; MDA-MB-468, 4 × 10^4^ cells per chamber; SKBR3, 4 × 10^4^ cells per chamber; Hs578T, 5 × 10^4^ cells per chamber). The outer chamber contained 600 μl complete media. Cells were incubated for 24 h (MCF-10A) or 12 h (MDA-MB-231, MDA-MB-468, SKBR3, and Hs578T) and then nonmigrating cells on the inside of the membrane were removed carefully with cotton swabs, while migrated cells on the outside of the membrane were fixed with 4% paraformaldehyde and stained with 0.1% Crystal violet in 70% methanol for 20 min, photographed under a light microscope, and analyzed by Image J.

### Immunofluorescent assay

For immunofluorescent assay, cells grown on coverslips were fixed with 4% polyformaldehyde and permeabilized with 0.3% Triton X-100 in PBS at room temperature. After blocked with 4% bovine serum albumin in PBS, the cells were then rinsed with PBS hybridized to an appropriate primary antibody, and FITC-conjugated secondary antibody (Jackson ImmunoResearch, Westgrove, Pennsylvania, USA), PE-conjugated secondary antibody (Jackson ImmunoResearch) and counter-staining using DAPI (Beyotime) for subsequent detection. Coverslips were mounted with ProLong Gold antifade reagent (Invitrogen). The cells were observed under the Leica TCS SP5 II system.

### In vivo metastasis assay using tail vein injection

MDA-MB-231 or MCF-10A stable cells were obtained and used in the tail vein injection in BALB/c nude mice. Briefly, 6.0 × 10^5^ MDA-MB-231 cells or 5.0 × 10^6^ MCF-10A cells in 100 μl PBS were injected into the lateral tail vein of 5-week-old female BALB/c nude mice (5 mice per group). Seven weeks after injection, the lungs of mice were dissected, fixed with 4% polyformaldehyde, and inspected for metastatic nodules under a dissecting microscope, then embedded in paraffin. Lung structure of each sample was observed and photographed after HE staining. All studies involving mice were approved by the Institutional Animal Care and Use Committee.

### Immunohistological chemistry (IHC) and tissue microarray

TMA slides of human breast cancer specimens (HBreD030PG03 and HBreD020PG01) were purchased (OUTDO, Shanghai, China). Antibodies specific against FBXO3 (SC-514625) antibody from Santa Cruz Biotechnology, Twist1 antibody (RT1635) from HuaBio, and USP4 antibody (612819) from zenbioscience were used for immunohistochemistry (IHC) staining. FBXO3, USP4, and Twist1 staining were analyzed by calculating the integrated optical density (IOD) using Image-Pro Plus 6.0 (Media Cybernetics, Massachusetts, USA), and the average optical density (AOD) was calculated using the formula: AOD = IOD/Area as described [49].

### Bioinformatics

The data were analyzed using Prism 8.0 (GraphPad). Paired two-tailed Student’s *t* tests were used to compare the means between groups. One-way or two-way ANOVA was used to assess changes in values for serial measurements over time. Significance was set at *p* < 0.05. Kaplan–Meier plots of OS and progression-free survival (RFS) of human breast cancer stratified by the PIK3CA, FBXO3, USP4, or Twist1 mRNA expression levels were analyzed using the Kaplan–Meier survival datasets. For multigene analysis of OS and RFS in the Kaplan–Meier survival datasets, the population was stratified by the mean mRNA expression levels of a set of 4 genes (PIK3CA, FBXO3, USP4, and Twist1).

## Supporting information

S1 Data Underlying numerical data and statistical analysis for Figs 1C, 1F, 1I, 2D, 2G, 2I, 3B, 3D, 3L, 3N, 3O, 5E, 5I, 5K, 6B, 6D, 6G, 6H, 6I, 6J and 6K and S2A, S2C, S3A, S3B, S3D, S4A, S4C, and S4E Figs. (XLSX)

S1 Raw Images Original images supporting all western blot results reported in Figs 1A, 1D, 1G, 2A, 2B, 2C, 3A, 3C, 3E, 3F, 3G, 3H, 3I, 3J, 3K, 4A, 4B, 4C, 4D, 4E, 4F, 4G, 4H, 4I, 5A, 5B, 5C, 5D, 5E, 5F, 5G, 5H, 5J, 5L, 6A and 6F and S1A, S2B, S2D, S3C, S4B, S4E, S4F and S4G Figs. The experimental samples, loading order, and molecular weight markers are indicated.
(PDF)

S1 Fig FBXO3 promotes cell migration in an E3 ubiquitin ligase-independent manner. (A) MCF-10A, MDA-MB-231, MDA-MB-468, SKBR3, or Hs578T cells were subjected to western blot analysis. (B) MDA-MB-231 stable cells expressing specific shRNA against FBXO3 (shFBXO3-#1 or shFBXO3-#2) or GFP (shCtrl) were subjected to wound-healing assays. Scale bar = 100 μm. (C, D) MDA-MB-231 or MCF-10A stable cells expressing HA-FBXO3^WT^, HA-FBXO3^ΔF^, or a vector control (Vec) were subjected to wound-healing assays. Scale bar = 100 μm.
(TIF)

S2 Fig FBXO3 has little effect on the steady-state mRNA levels of Twist1. (A) MDA-MB-231 stable cells were subjected to qRT-PCR assays. Data were derived from 3 independent experiments. (B, C) MCF-10A stably expressing HA-FBXO3 was treated with cycloheximide (CHX) for the indicted times prior to western blot analyses (B). The relative Twist1 protein expression levels were quantitated by image J software (C). (D) Hs578T cells stably expressing HA-FBXO3^ΔF^ were infected with a recombinant lentivirus expressing specific shRNA targeting to USP4 or a vector control (-) were subjected to western blot analyses. (E, F) MDA-MB-231 cells stably expressing HA-FBXO3^WT^, HA-FBXO3^ΔF^, or a vector control (Vec) were infected with a recombinant lentivirus expressing specific shRNA targeting to USP4 or GFP (-). Stable cells were then subjected to transwell assays. Scale bar = 100 μm. The data underlying the graphs shown in the figure can be found in S1 Data.
(TIF)

S3 Fig FBXO3 does not affect USP4 gene transcription. (A, B) MDA-MB-231 stable cells were subjected to qRT-PCR assays. Data were derived from 3 independent experiments. (C, D) MDA-MB-231 stable cells were treated with cycloheximide (CHX) for the indicted times prior to western blot analyses (C). The relative USP4 protein expression levels were quantitated by image J software (D). The data underlying the graphs shown in the figure can be found in S1 Data.
(TIF)

S4 Fig Ectopic expression of p110α^H1047R^ up-regulates FBXO3 and USP4 protein expression. (A) MCF-10A, MDA-MB-231, MDA-MB-468, SKBR3, Hs578T, or MDA-MB-453 cells were subjected to transwell assays. Cells were suspended in serum-free media, seeded into the transwell inner chamber (5 × 10^4^ cells per chamber) and incubated for 24 h. Scale bar = 100 μm. (B, C) MCF-10A stably expressing p110α, p110α^E542K^, p110α^E545K^, p110α^H1047R^ or a vector control (Vec) were subjected to western blot analyses (B) and transwell assays (C). Scale bar = 100 μm. (D) MDA-MB-231 cells stably expressing p110α^H1047R^ or a vector control (Vec) were subjected to Immunofluorescence staining for USP4 (green) and FBXO3 (red) and counterstained with DAPI. Scale bar = 50 μm. (E) MCF-10A cells stably expressing p110α^H1047R^ or a vector control (Vec) were treated with cycloheximide (CHX) for the indicted times, and the relative FBXO3 protein expression levels were quantified by image J software. (F, G) HEK293T cells were co-transfected with p110α^H1047R^ and either HA-FBXO3 wild-type (WT) or an HA-FBXO3 mutant (S26A, T82A, S166A, S356A, or Y233A) expressing plasmids for 36 h, and cells were then subjected to western blot analyses. The data underlying the graphs shown in the figure can be found in S1 Data.
(TIF)

S5 Fig Ectopic expression of p110α^H1047R^ induces cell migration by up-regulation of FBXO3 and USP4. (A, B) MCF-10A cells stably expressing p110α^H1047R^ were infected with a recombinant lentivirus expressing specific shRNA targeting to FBXO3 or GFP (shCtrl). Cells were then subjected to transwell assays (A) and wound-healing assays (B). Scale bar = 100 μm. (C, D) MDA-MB-231 or MCF-10A cells stably expressing p110α^H1047R^ were infected with a recombinant lentivirus expressing specific shRNA targeting to USP4 or GFP (-). Cells were then subjected to transwell analyses. Scale bar = 100 μm.
(TIF)

## Abbreviations

AIRE: autoimmune regulator
AOD: average optical density
EMT: epithelial–mesenchymal transition
FBS: fetal bovine serum
HIF1α: hypoxia-inducible factor 1α
IHC: immunohistochemistry
IOD: integrated optical density
LPS: lipopolysaccharide
MAPK: mitogen-activated protein kinase
MMP: matrix metalloproteinase
OS: overall survival
RFS: recurrence-free survival
TGF: transforming growth factor
TMA: tissue microarray
TRAF: tumor necrosis factor receptor-associated factor
USP4: ubiquitin-specific protease 4
VEGF: vascular endothelial growth factor

## References

[1] DeSantis CE, Ma J, Gaudet MM, Newman LA, Miller KD, Goding Sauer A, et al. Breast cancer statistics, 2019. CA Cancer J Clin. 2019;69:438–451. doi: 10.3322/caac.21583 31577379

[2] Sandoo A, Kitas GD, Carmichael AR. Breast cancer therapy and cardiovascular risk: focus on trastuzumab. Vasc Health Risk Manag. 2015;11:223–228. doi: 10.2147/VHRM.S69641 25897242 PMC4397929

[3] Harbeck N, Penault-Llorca F, Cortes J, Gnant M, Houssami N, Poortmans P, et al. Breast cancer. Nat Rev Dis Primers. 2019;5:66. doi: 10.1038/s41572-019-0111-2 31548545

[4] Gupta P, Adkins C, Lockman P, Srivastava SK. Metastasis of Breast Tumor Cells to Brain Is Suppressed by Phenethyl Isothiocyanate in a Novel In Vivo Metastasis Model. PLoS ONE. 2013;8:e67278. doi: 10.1371/journal.pone.0067278 23826254 PMC3695065

[5] Miricescu D, Totan A, Stanescu S II, Badoiu SC, Stefani C, Greabu M. PI3K/AKT/mTOR Signaling Pathway in Breast Cancer: From Molecular Landscape to Clinical Aspects. Int J Mol Sci. 2020;22. 33375317 10.3390/ijms22010173 PMC7796017

[6] Jin Y, Chen W, Yang H, Yan Z, Lai Z, Feng J, et al. Don inhibits migration and invasion of colorectal cancer cells via suppression of PI3K/AKT and TGF-beta/Smad signaling pathways. Exp Ther Med. 2017;14:5527–5534. 29285087 10.3892/etm.2017.5242 PMC5740531

[7] Reddy KB, Nabha SM, Atanaskova N. Role of MAP kinase in tumor progression and invasion. Cancer Metastasis Rev. 2003;22:395–403. doi: 10.1023/a:1023781114568 12884914

[8] Hao Y, Baker D, Ten Dijke P. TGF-beta-Mediated Epithelial-Mesenchymal Transition and Cancer Metastasis. Int J Mol Sci. 2019;20. 31195692 10.3390/ijms20112767 PMC6600375

[9] Ellis H, Ma CX. PI3K Inhibitors in Breast Cancer Therapy. Curr Oncol Rep. 2019;21:110. doi: 10.1007/s11912-019-0846-7 31828441

[10] Thorpe LM, Yuzugullu H, Zhao JJ. PI3K in cancer: divergent roles of isoforms, modes of activation and therapeutic targeting. Nat Rev Cancer. 2015;15:7–24. doi: 10.1038/nrc3860 25533673 PMC4384662

[11] Wan G, Pehlke C, Pepermans R, Cannon JL, Lidke D, Rajput A. The H1047R point mutation in p110 alpha changes the morphology of human colon HCT116 cancer cells. Cell Death Dis. 2015;1:15044. doi: 10.1038/cddiscovery.2015.44 27551473 PMC4979441

[12] Ross RL, Askham JM, Knowles MA. PIK3CA mutation spectrum in urothelial carcinoma reflects cell context-dependent signaling and phenotypic outputs. Oncogene. 2013;32:768–776. doi: 10.1038/onc.2012.87 22430209

[13] Fruman DA, Rommel C. PI3K and cancer: lessons, challenges and opportunities. Nat Rev Drug Discov. 2014;13:140–156. doi: 10.1038/nrd4204 24481312 PMC3994981

[14] Ebi H, Costa C, Faber AC, Nishtala M, Kotani H, Juric D, et al. PI3K regulates MEK/ERK signaling in breast cancer via the Rac-GEF, P-Rex1. Proc Natl Acad Sci U S A. 2013;110:21124–21129. doi: 10.1073/pnas.1314124110 24327733 PMC3876254

[15] Fattahi S, Amjadi-Moheb F, Tabaripour R, Ashrafi GH, Akhavan-Niaki H. PI3K/AKT/mTOR signaling in gastric cancer: Epigenetics and beyond. Life Sci. 2020;262:118513. doi: 10.1016/j.lfs.2020.118513 33011222

[16] Jiang N, Dai Q, Su X, Fu J, Feng X, Peng J. Role of PI3K/AKT pathway in cancer: the framework of malignant behavior. Mol Biol Rep. 2020;47:4587–4629. doi: 10.1007/s11033-020-05435-1 32333246 PMC7295848

[17] Hu L, Liang S, Chen H, Lv T, Wu J, Chen D, et al. DeltaNp63alpha is a common inhibitory target in oncogenic PI3K/Ras/Her2-induced cell motility and tumor metastasis. Proc Natl Acad Sci U S A. 2017;114:E3964–E3973. 28468801 10.1073/pnas.1617816114 PMC5441775

[18] Mittal V. Epithelial Mesenchymal Transition in Tumor Metastasis. Annu Rev Pathol. 2018;13:395–412. doi: 10.1146/annurev-pathol-020117-043854 29414248

[19] De Craene B, Berx G. Regulatory networks defining EMT during cancer initiation and progression. Nat Rev Cancer. 2013;13:97–110. doi: 10.1038/nrc3447 23344542

[20] Qin Q, Xu Y, He T, Qin C, Xu J. Normal and disease-related biological functions of Twist1 and underlying molecular mechanisms. Cell Res. 2012;22:90–106. doi: 10.1038/cr.2011.144 21876555 PMC3351934

[21] Yang MH, Wu MZ, Chiou SH, Chen PM, Chang SY, Liu CJ, et al. Direct regulation of TWIST by HIF-1alpha promotes metastasis. Nat Cell Biol. 2008;10:295–305. doi: 10.1038/ncb1691 18297062

[22] Hong J, Zhou J, Fu J, He T, Qin J, Wang L, et al. Phosphorylation of serine 68 of Twist1 by MAPKs stabilizes Twist1 protein and promotes breast cancer cell invasiveness. Cancer Res. 2011;71:3980–3990. doi: 10.1158/0008-5472.CAN-10-2914 21502402 PMC3107354

[23] Li F, Hu Q, He T, Xu J, Yi Y, Xie S, et al. The Deubiquitinase USP4 Stabilizes Twist1 Protein to Promote Lung Cancer Cell Stemness. Cancers (Basel). 2020;12. 32549341 10.3390/cancers12061582 PMC7352958

[24] Zhou F, Xie F, Jin K, Zhang Z, Clerici M, Gao R, et al. USP4 inhibits SMAD4 monoubiquitination and promotes activin and BMP signaling. EMBO J. 2017;36:1623–1639. doi: 10.15252/embj.201695372 28468752 PMC5452037

[25] Zhou F, Zhang X, van Dam H, Ten Dijke P, Huang H, Zhang L. Ubiquitin-specific protease 4 mitigates Toll-like/interleukin-1 receptor signaling and regulates innate immune activation. J Biol Chem. 2012;287:11002–11010. doi: 10.1074/jbc.M111.328187 22262844 PMC3322833

[26] Hu B, Zhang D, Zhao K, Wang Y, Pei L, Fu Q, et al. Spotlight on USP4: Structure, Function, and Regulation, Front Cell. Dev Biol. 2021;9:595159. 10.3389/fcell.2021.595159 PMC7935551 33681193

[27] Wang Y, Zhou L, Lu J, Jiang B, Liu C, Guo J. USP4 function and multifaceted roles in cancer: a possible and potential therapeutic target. Cancer Cell Int. 2020;20:298. doi: 10.1186/s12935-020-01391-9 32669974 PMC7350758

[28] Cao WH, Liu XP, Meng SL, Gao YW, Wang Y, Ma ZL, et al. USP4 promotes invasion of breast cancer cells via Relaxin/TGF-beta1/Smad2/MMP-9 signal. Eur Rev Med Pharmacol Sci. 2016;20:1115–1122. 27049265

[29] Zhang L, Zhou F, Drabsch Y, Gao R, Snaar-Jagalska BE, Mickanin C, et al. USP4 is regulated by AKT phosphorylation and directly deubiquitylates TGF-beta type I receptor. Nat Cell Biol. 2012;14:717–726. 22706160 10.1038/ncb2522

[30] Xing C, Lu XX, Guo PD, Shen T, Zhang S, He XS, et al. Ubiquitin-Specific Protease 4-Mediated Deubiquitination and Stabilization of PRL-3 Is Required for Potentiating Colorectal Oncogenesis. Cancer Res. 2016;76:83–95. doi: 10.1158/0008-5472.CAN-14-3595 26669864

[31] Xiao N, Li H, Luo J, Wang R, Chen H, Chen J, et al. Ubiquitin-specific protease 4 (USP4) targets TRAF2 and TRAF6 for deubiquitination and inhibits TNFalpha-induced cancer cell migration. Biochem J. 2012;441:979–986. 22029577 10.1042/BJ20111358

[32] Chen BB, Coon TA, Glasser JR, McVerry BJ, Zhao J, Zhao Y, et al. A combinatorial F box protein directed pathway controls TRAF adaptor stability to regulate inflammation. Nat Immunol. 2013;14:470–479. doi: 10.1038/ni.2565 23542741 PMC3631463

[33] Li Z, Fan S, Wang J, Chen X, Liao Q, Liu X, et al. Zebrafish F-box Protein fbxo3 Negatively Regulates Antiviral Response through Promoting K27-Linked Polyubiquitination of the Transcription Factors irf3 and irf7. J Immunol. 2020;205:1897–1908. doi: 10.4049/jimmunol.2000305 32859728

[34] Shao W, Zumer K, Fujinaga K, Peterlin BM. FBXO3 Protein Promotes Ubiquitylation and Transcriptional Activity of AIRE (Autoimmune Regulator). J Biol Chem. 2016;291:17953–17963. doi: 10.1074/jbc.M116.724401 27365398 PMC5016183

[35] Kainulainen M, Habjan M, Hubel P, Busch L, Lau S, Colinge J, et al. Virulence factor NSs of rift valley fever virus recruits the F-box protein FBXO3 to degrade subunit p62 of general transcription factor TFIIH. J Virol. 2014;88:3464–3473. doi: 10.1128/JVI.02914-13 24403578 PMC3957945

[36] Zhang Z, Bao Z, Gao P, Yao J, Wang P, Chai D. Diverse Roles of F-BoxProtein3 in Regulation of Various Cellular Functions. Front Cell Dev Biol. 2021;9:802204. doi: 10.3389/fcell.2021.802204 35127719 PMC8807484

[37] Pan J, Fang S, Tian H, Zhou C, Zhao X, Tian H, et al. lncRNA JPX/miR-33a-5p/Twist1 axis regulates tumorigenesis and metastasis of lung cancer by activating Wnt/beta-catenin signaling. Mol Cancer. 2020;19:9. 31941509 10.1186/s12943-020-1133-9 PMC6961326

[38] Geng N, Li Y, Zhang W, Wang F, Wang X, Jin Z, et al. A PAK5-DNPEP-USP4 axis dictates breast cancer growth and metastasis. Int J Cancer. 2020;146:1139–1151. doi: 10.1002/ijc.32523 31219614

[39] Lien EC, Dibble CC, Toker A. PI3K signaling in cancer: beyond AKT. Curr Opin Cell Biol. 2017;45:62–71. doi: 10.1016/j.ceb.2017.02.007 28343126 PMC5482768

[40] Samuels Y, Diaz LA Jr, Schmidt-Kittler O, Cummins JM, Delong L, Cheong I, et al. Mutant PIK3CA promotes cell growth and invasion of human cancer cells. Cancer Cell. 2005;7:561–573. doi: 10.1016/j.ccr.2005.05.014 15950905

[41] She QB, Chandarlapaty S, Ye Q, Lobo J, Haskell KM, Leander KR, et al. Breast tumor cells with PI3K mutation or HER2 amplification are selectively addicted to Akt signaling. PLoS ONE. 2008;3:e3065. doi: 10.1371/journal.pone.0003065 18725974 PMC2516933

[42] Mallampalli RK, Coon TA, Glasser JR, Wang C, Dunn SR, Weathington NM, et al. Targeting F box protein Fbxo3 to control cytokine-driven inflammation. J Immunol. 2013;191:5247–5255. doi: 10.4049/jimmunol.1300456 24123678 PMC3845358

[43] Niu M, Xu J, Liu Y, Li Y, He T, Ding L, et al. FBXL2 counteracts Grp94 to destabilize EGFR and inhibit EGFR-driven NSCLC growth. Nat Commun. 2021;12:5919. doi: 10.1038/s41467-021-26222-x 34635651 PMC8505509

[44] Wijnhoven P, Konietzny R, Blackford AN, Travers J, Kessler BM, Nishi R, et al. USP4 Auto-Deubiquitylation Promotes Homologous Recombination. Mol Cell. 2015;60:362–373. doi: 10.1016/j.molcel.2015.09.019 26455393 PMC4643306

[45] Han S, Lear TB, Jerome JA, Rajbhandari S, Snavely CA, Gulick DL, et al. Lipopolysaccharide Primes the NALP3 Inflammasome by Inhibiting Its Ubiquitination and Degradation Mediated by the SCFFBXL2 E3 Ligase. J Biol Chem. 2015;290:18124–18133. doi: 10.1074/jbc.M115.645549 26037928 PMC4505057

[46] Weathington NM, Alvarez D, Sembrat J, Radder J, Cardenes N, Noda K, et al. Ex vivo lung perfusion as a human platform for preclinical small molecule testing. JCI Insight. 2018;3. doi: 10.1172/jci.insight.95515 30282819 PMC6237445

[47] Li J, Ma L. MiR-142-3p Attenuates Oxygen Glucose Deprivation/Reoxygenation-Induced Injury by Targeting FBXO3 in Human Neuroblastoma SH-SY5Y Cells. World Neurosurg. 2020;136:e149–e157. doi: 10.1016/j.wneu.2019.12.064 31863884

[48] Sun S, Chen H, Sun L, Wang M, Wu X, Xiao ZJ. Hotspot mutant p53-R273H inhibits KLF6 expression to promote cell migration and tumor metastasis. Cell Death Dis. 2020;11:595. doi: 10.1038/s41419-020-02814-1 32733026 PMC7393383

[49] Tilley WD, Lim-Tio SS, Horsfall DJ, Aspinall JO, Marshall VR, Skinner JM. Detection of discrete androgen receptor epitopes in prostate cancer by immunostaining: measurement by color video image analysis. Cancer Res. 1994;54:4096–4102. 7518349

