# Supplementary material for: FBXO3 stabilizes USP4 and Twist1 to promote PI3K-mediated breast cancer metastasis
Source: PLoS Biol. 2023 Dec 22;21(12):e3002446. doi: 10.1371/journal.pbio.3002446 (PMC10745200; doi:10.1371/journal.pbio.3002446)
Supplement: S1 Raw Images — The experimental samples, loading order, and molecular weight markers are indicated. (PDF) [file pbio.3002446.s002.PDF]

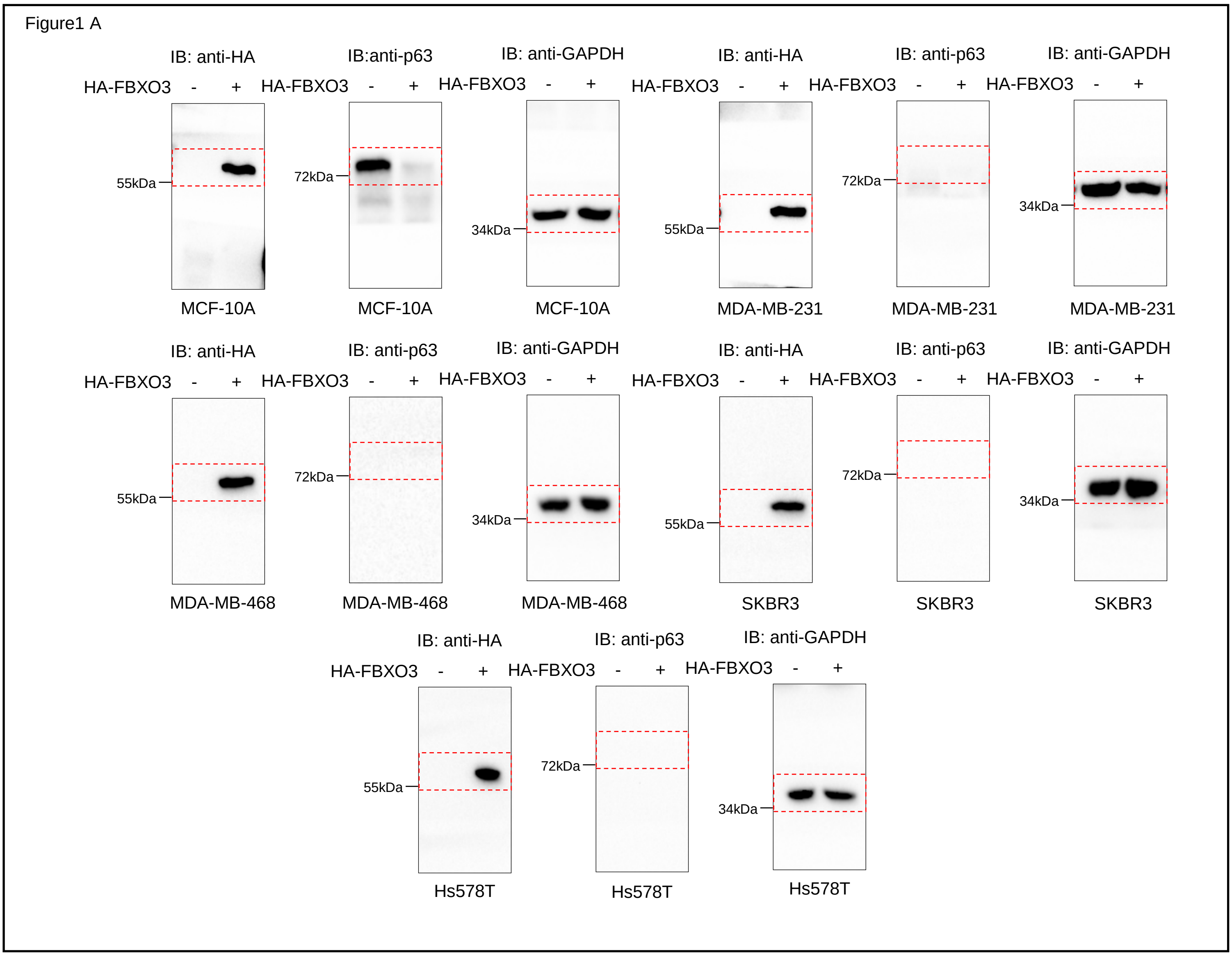
Figure1 A
IB: anti-HA
IB:anti-p63
IB: anti-GAPDH
IB: anti-HA
IB: anti-p63
IB: anti-GAPDH
HA-FBXO3 - +
55kDa
72kDa
34kDa
MCF-10A
MDA-MB-231
MDA-MB-468
SKBR3
Hs578T

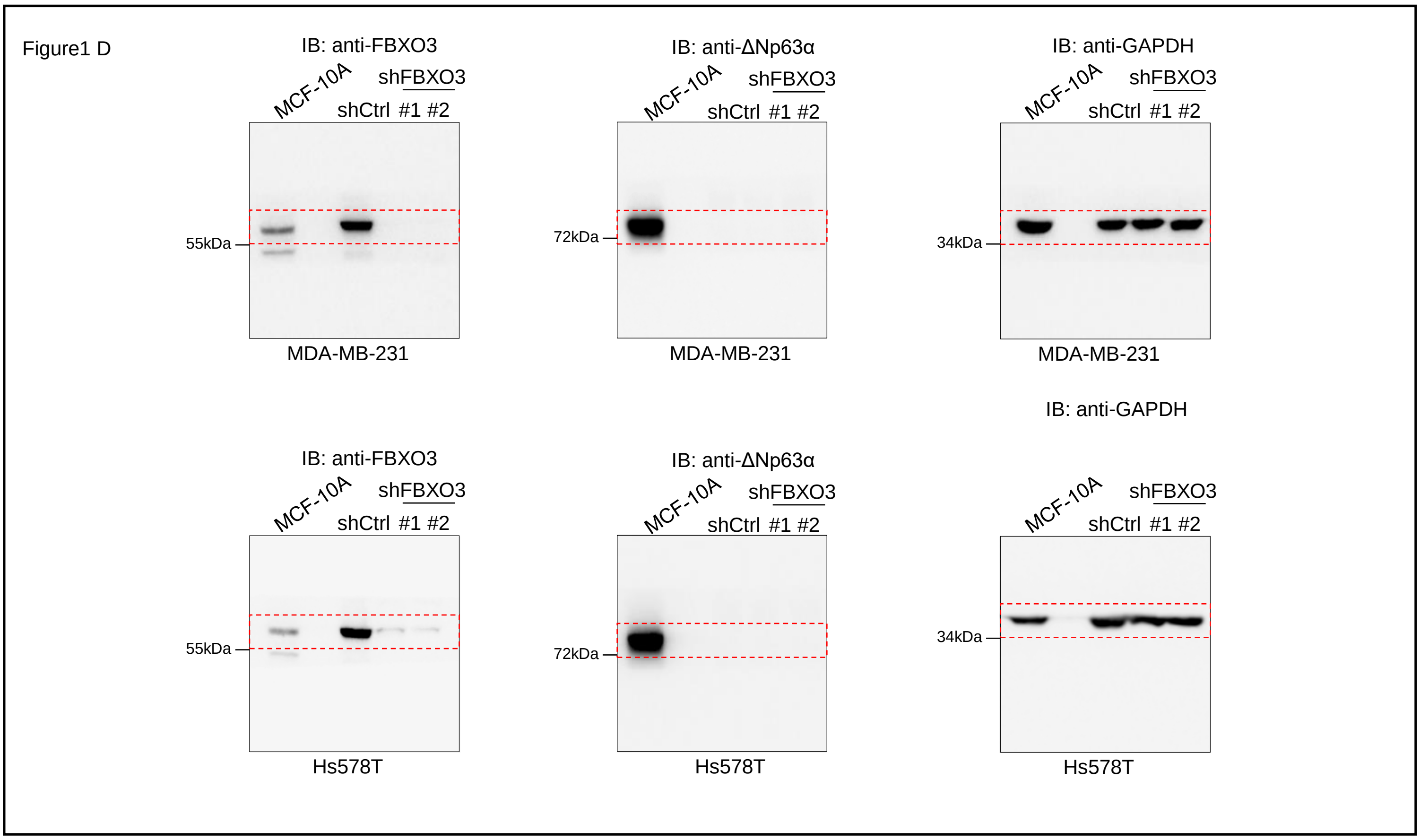
Figure1 D
IB: anti-FBXO3
IB: anti-ΔNp63α
IB: anti-GAPDH
MCF-10A
shFBXO3
shCtrl #1 #2
55kDa
72kDa
34kDa
MDA-MB-231
Hs578T

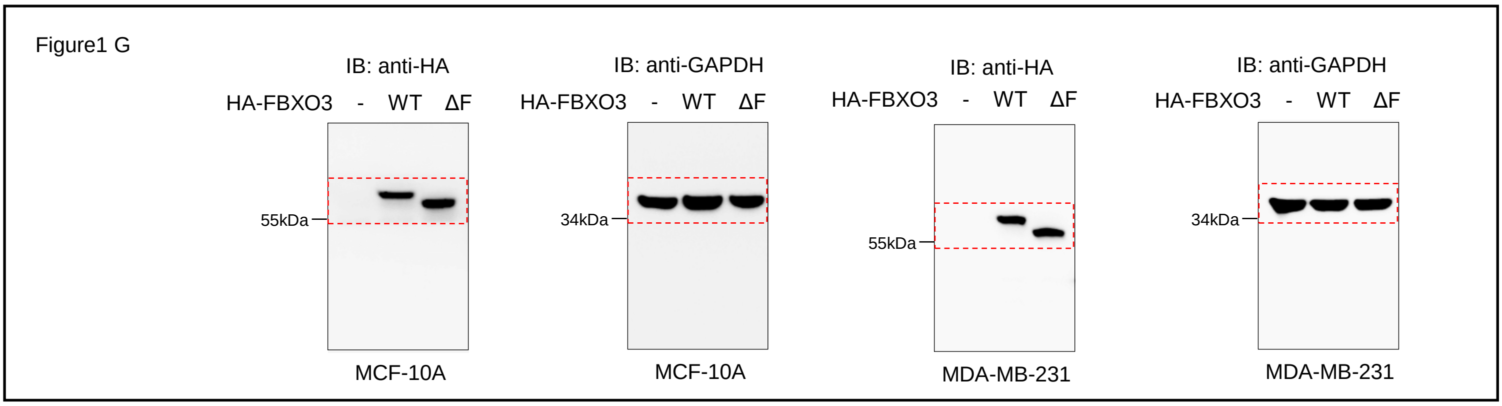
Figure1 G
IB: anti-HA
HA-FBXO3 - WT ΔF
55kDa
MCF-10A
IB: anti-GAPDH
HA-FBXO3 - WT ΔF
34kDa
MCF-10A
IB: anti-HA
HA-FBXO3 - WT ΔF
55kDa
MDA-MB-231
IB: anti-GAPDH
HA-FBXO3 - WT ΔF
34kDa
MDA-MB-231

Figure2 A

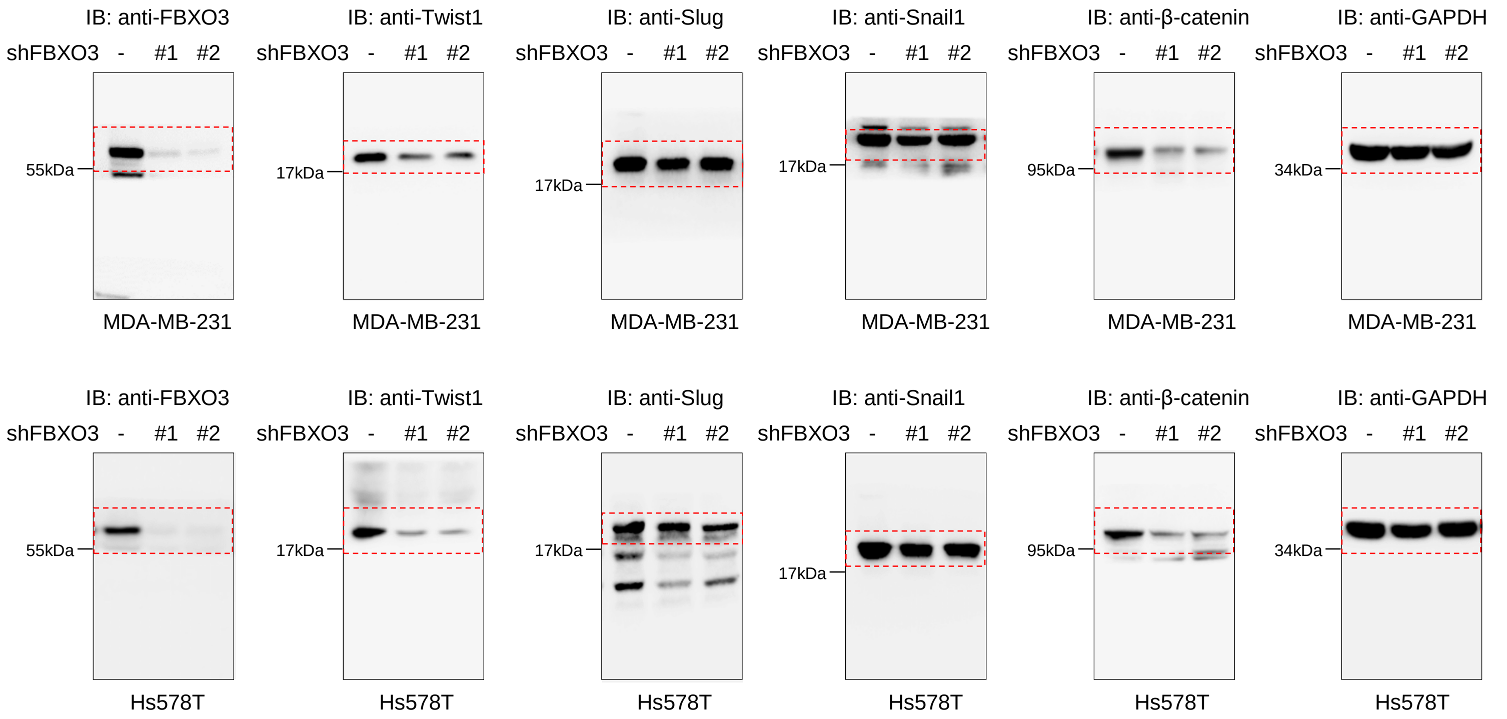

Figure2 B

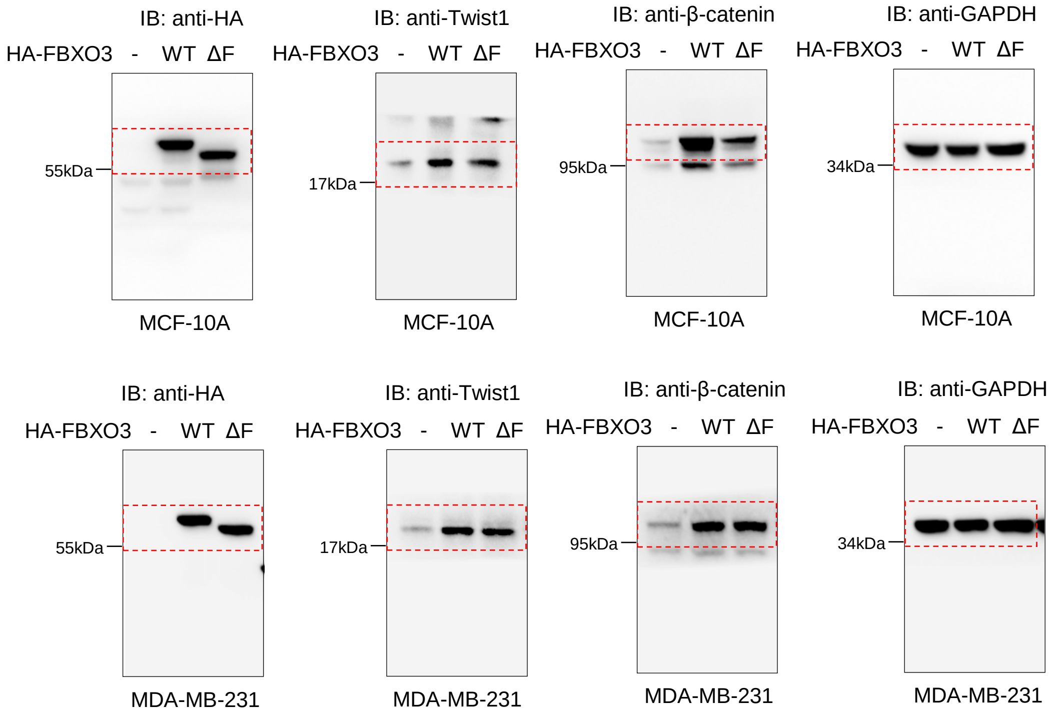

Figure2 C

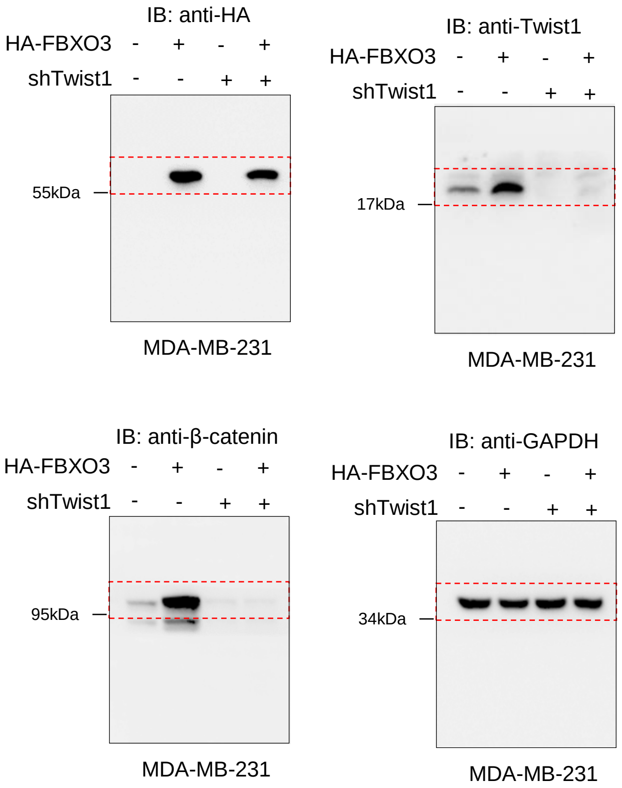
IB: anti-HA
HA-FBXO3 - + - +
shTwist1 - - + +
55kDa
MDA-MB-231
IB: anti-Twist1
HA-FBXO3 - + - +
shTwist1 - - + +
17kDa
MDA-MB-231
IB: anti-β-catenin
HA-FBXO3 - + - +
shTwist1 - - + +
95kDa
MDA-MB-231
IB: anti-GAPDH
HA-FBXO3 - + - +
shTwist1 - - + +
34kDa
MDA-MB-231

Figure3 A

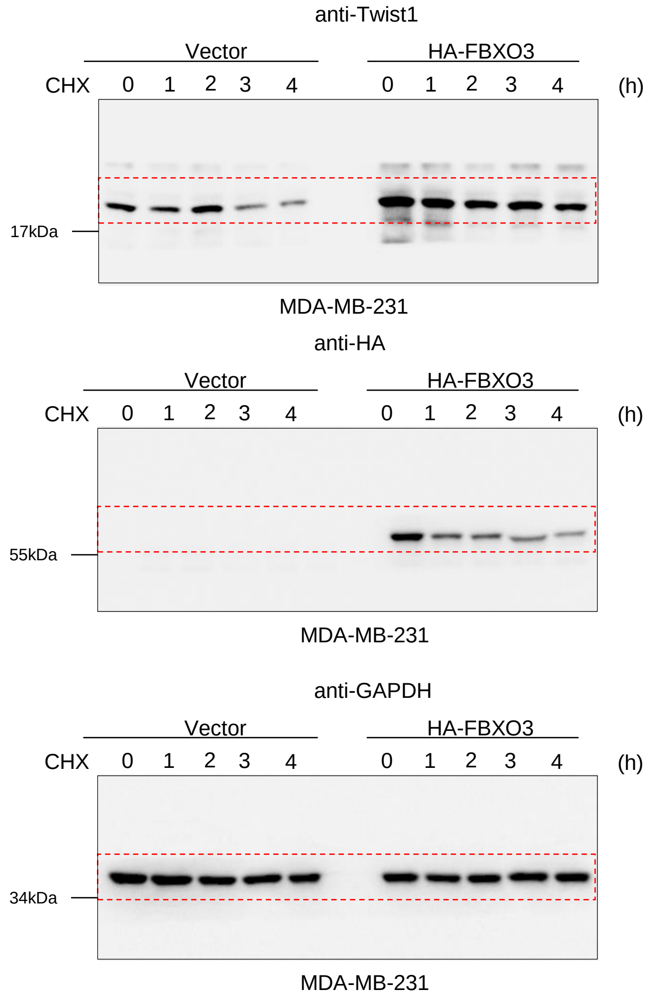

Figure3 C

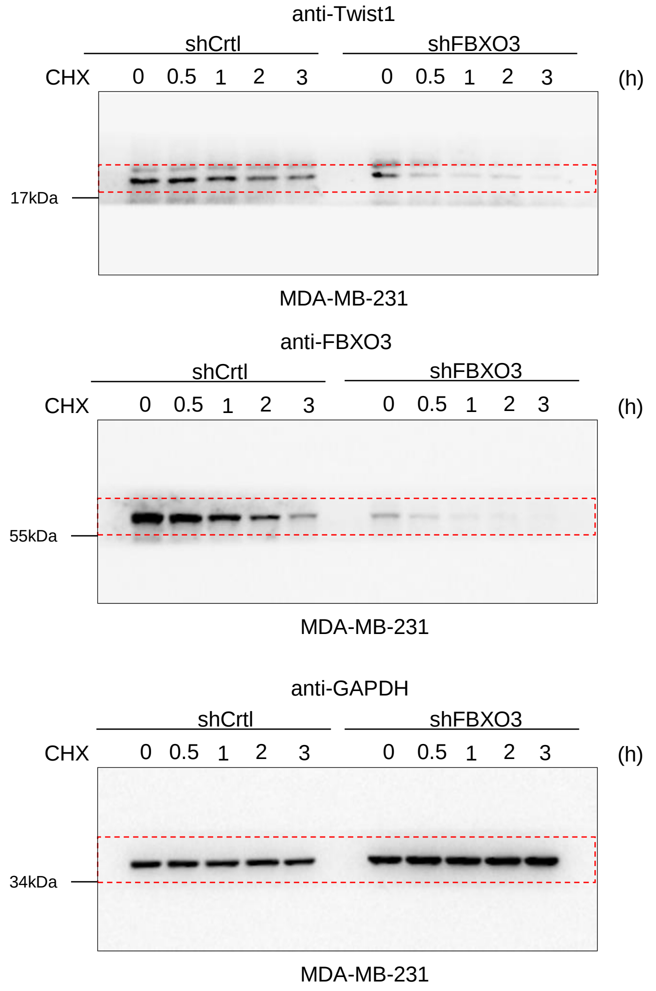

Figure3 E

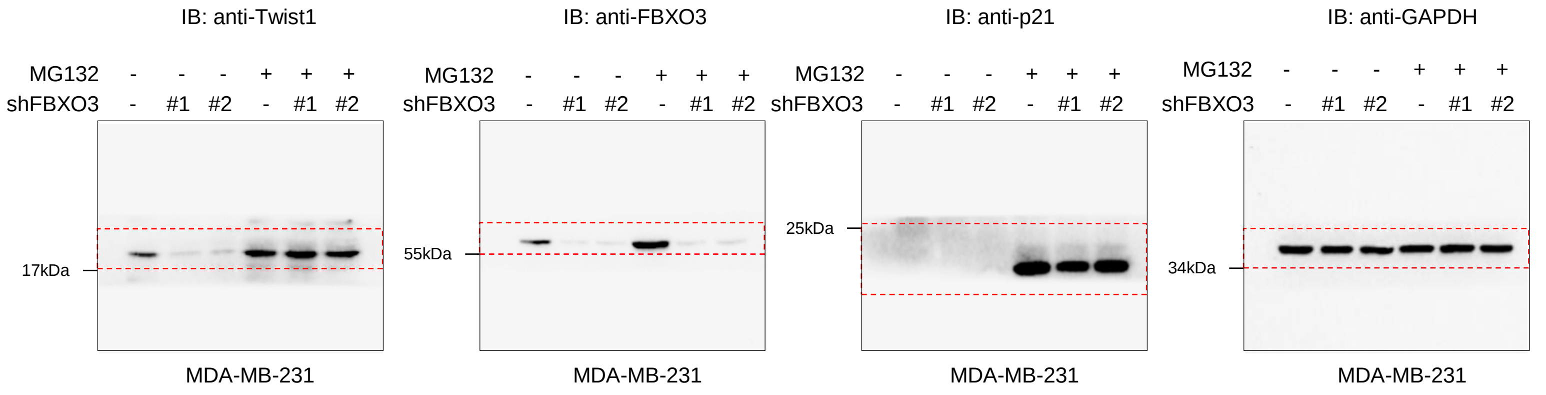

Figure3 F

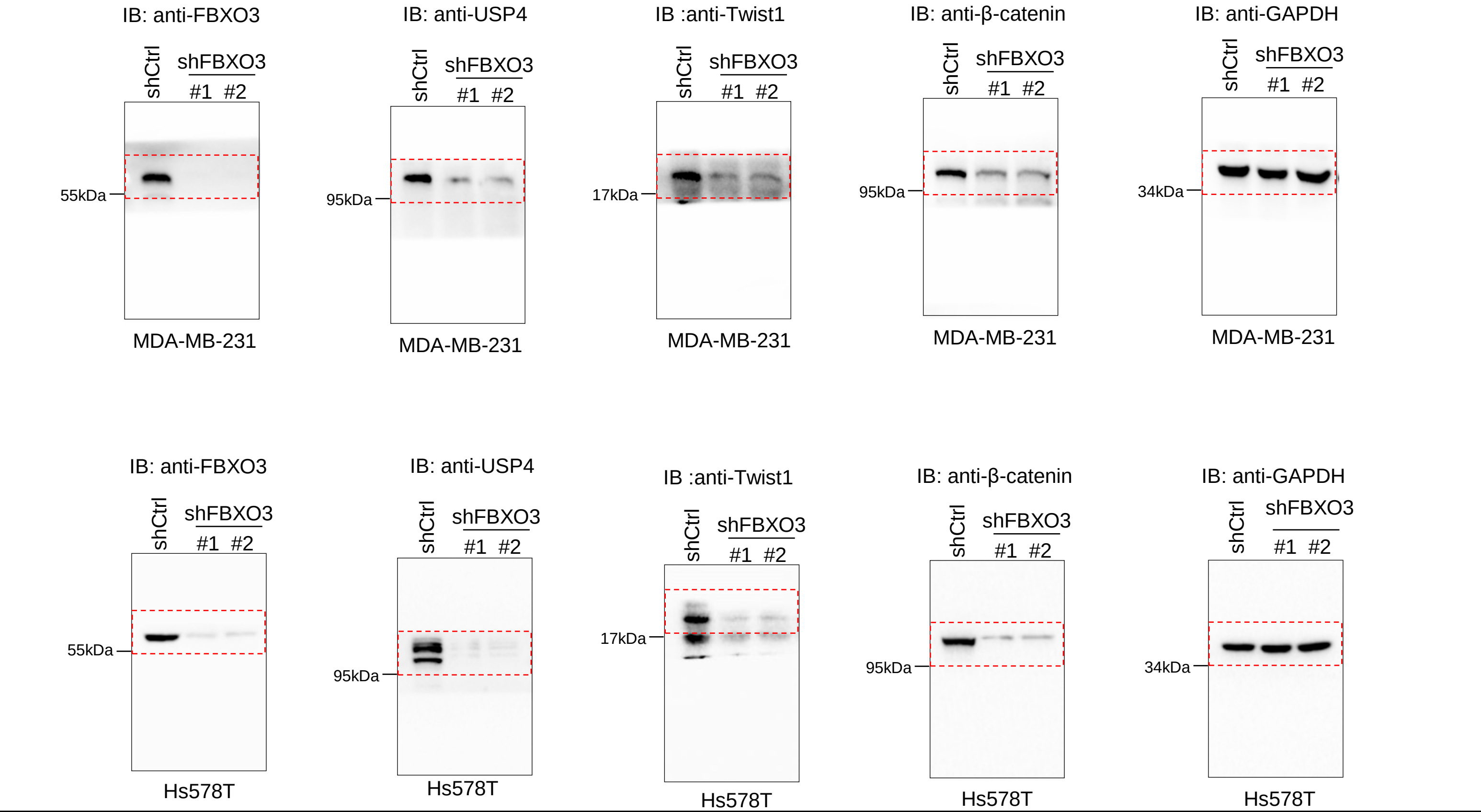

Figure3 G

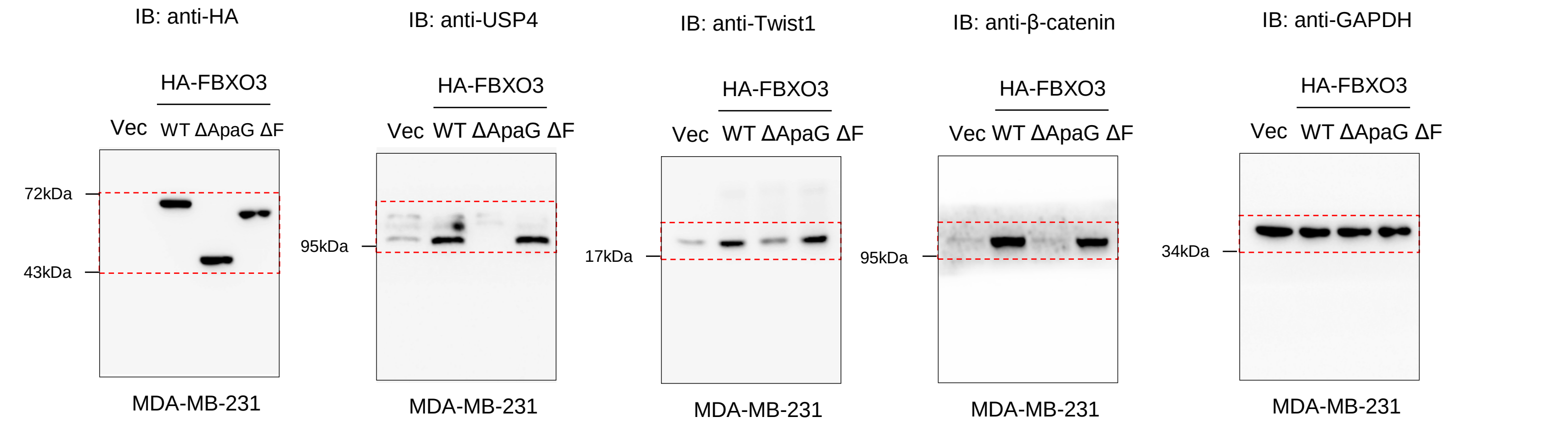

Figure3 H

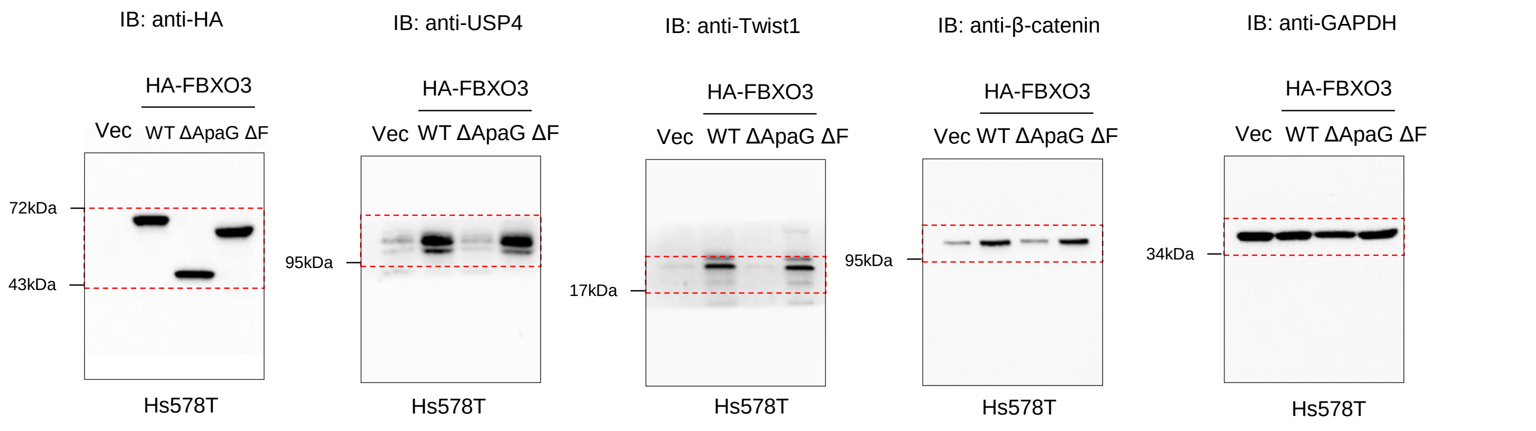

Figure3 I

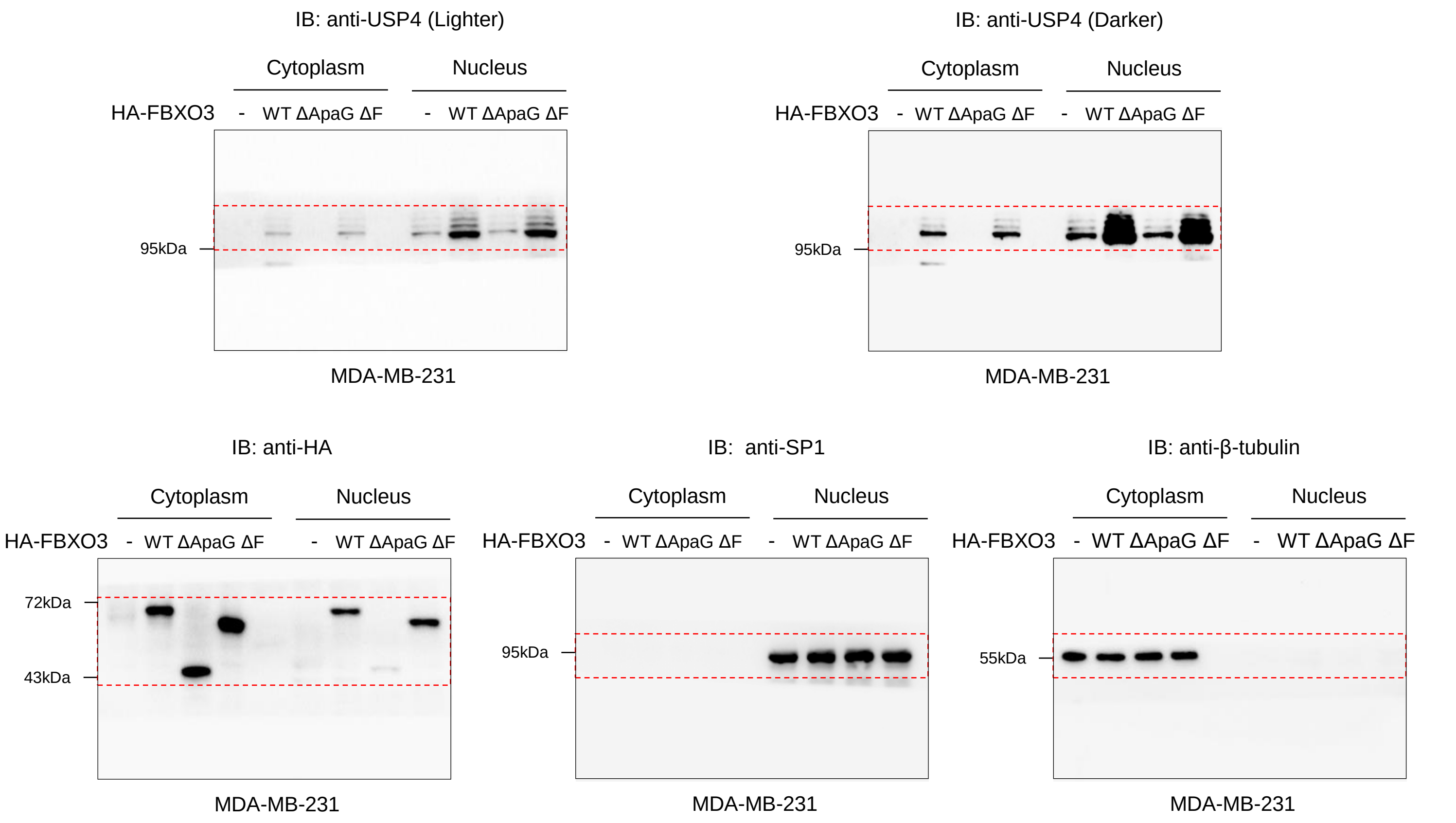

Figure3 J

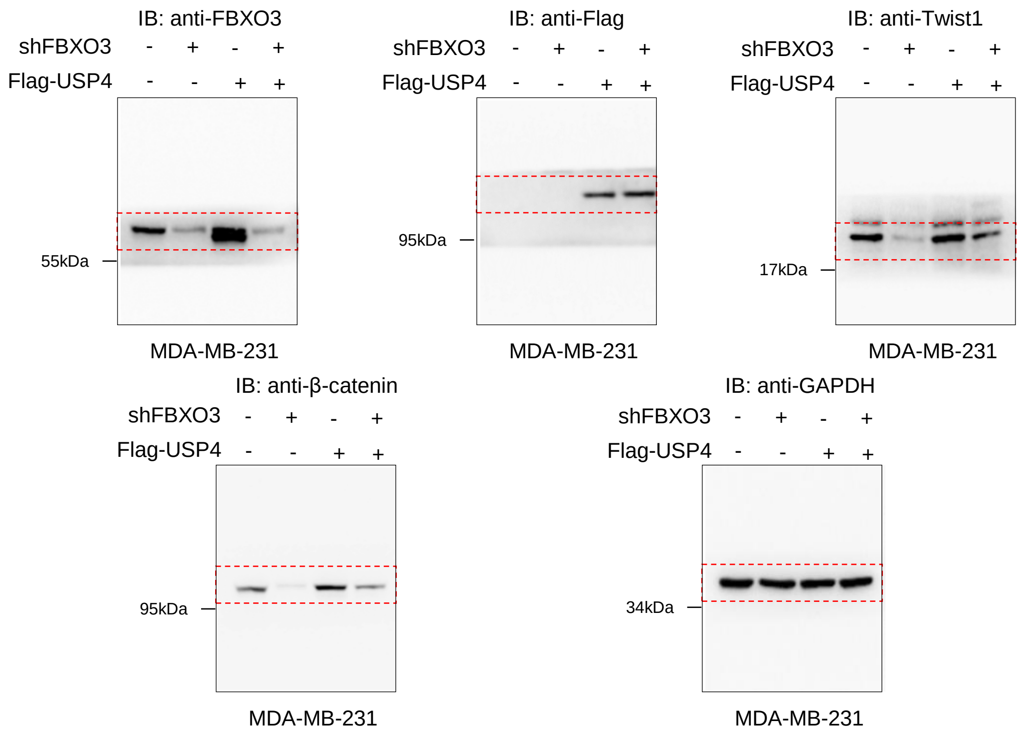

Figure3 K

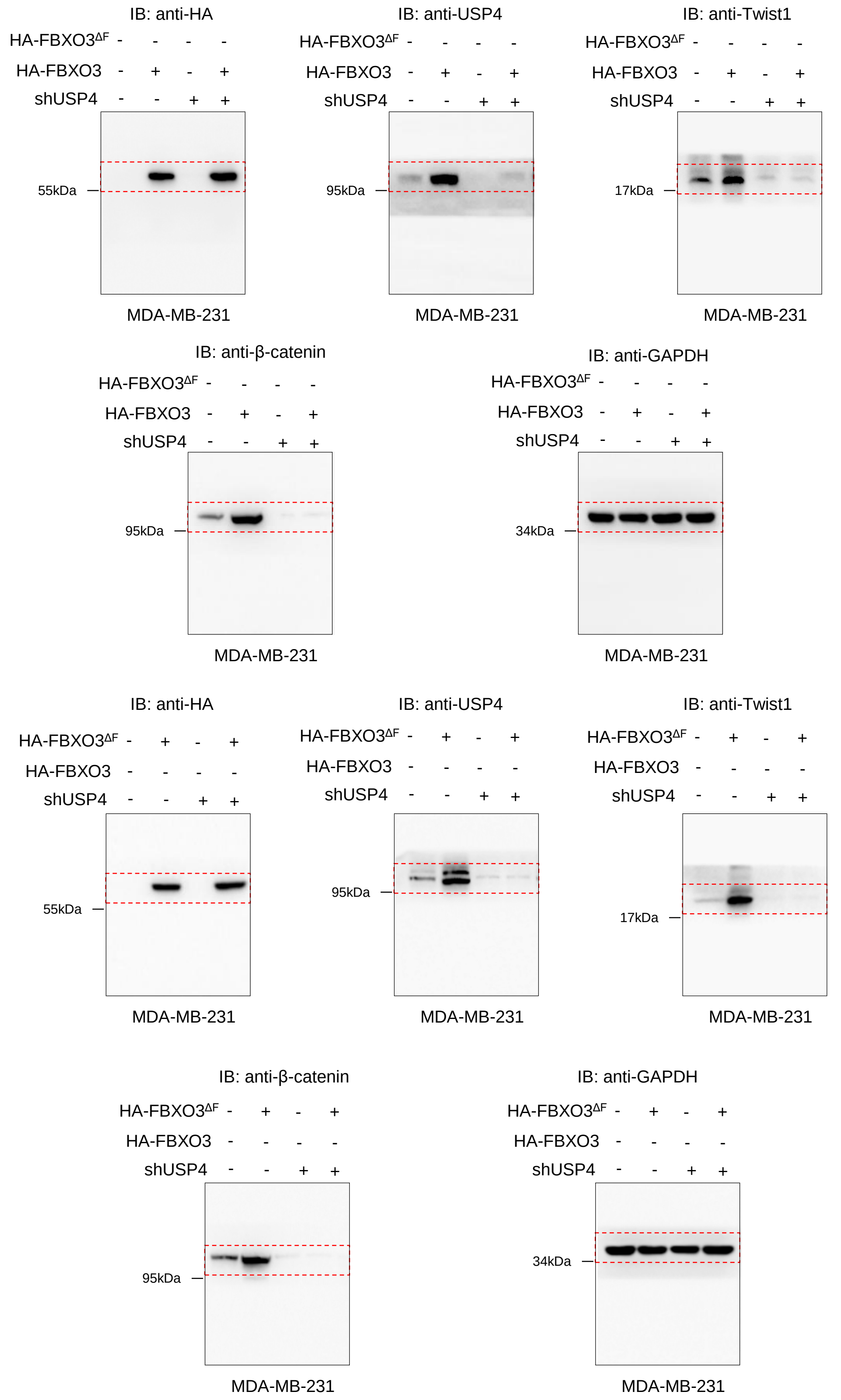

IB: anti-HA
HA-FBXO3ΔF - - - -
HA-FBXO3 - + - +
shUSP4 - - + +
55kDa
MDA-MB-231
IB: anti-USP4
HA-FBXO3ΔF - - - -
HA-FBXO3 - + - +
shUSP4 - - + +
95kDa
MDA-MB-231
IB: anti-Twist1
HA-FBXO3ΔF - - - -
HA-FBXO3 - + - +
shUSP4 - - + +
17kDa
MDA-MB-231
IB: anti-β-catenin
HA-FBXO3ΔF - - - -
HA-FBXO3 - + - +
shUSP4 - - + +
95kDa
MDA-MB-231
IB: anti-GAPDH
HA-FBXO3ΔF - - - -
HA-FBXO3 - + - +
shUSP4 - - + +
34kDa
MDA-MB-231
IB: anti-HA
HA-FBXO3ΔF - + - +
HA-FBXO3 - - - -
shUSP4 - - + +
55kDa
MDA-MB-231
IB: anti-USP4
HA-FBXO3ΔF - + - +
HA-FBXO3 - - - -
shUSP4 - - + +
95kDa
MDA-MB-231
IB: anti-Twist1
HA-FBXO3ΔF - + - +
HA-FBXO3 - - - -
shUSP4 - - + +
17kDa
MDA-MB-231
IB: anti-β-catenin
HA-FBXO3ΔF - + - +
HA-FBXO3 - - - -
shUSP4 - - + +
95kDa
MDA-MB-231
IB: anti-GAPDH
HA-FBXO3ΔF - + - +
HA-FBXO3 - - - -
shUSP4 - - + +
34kDa
MDA-MB-231

Figure4 A

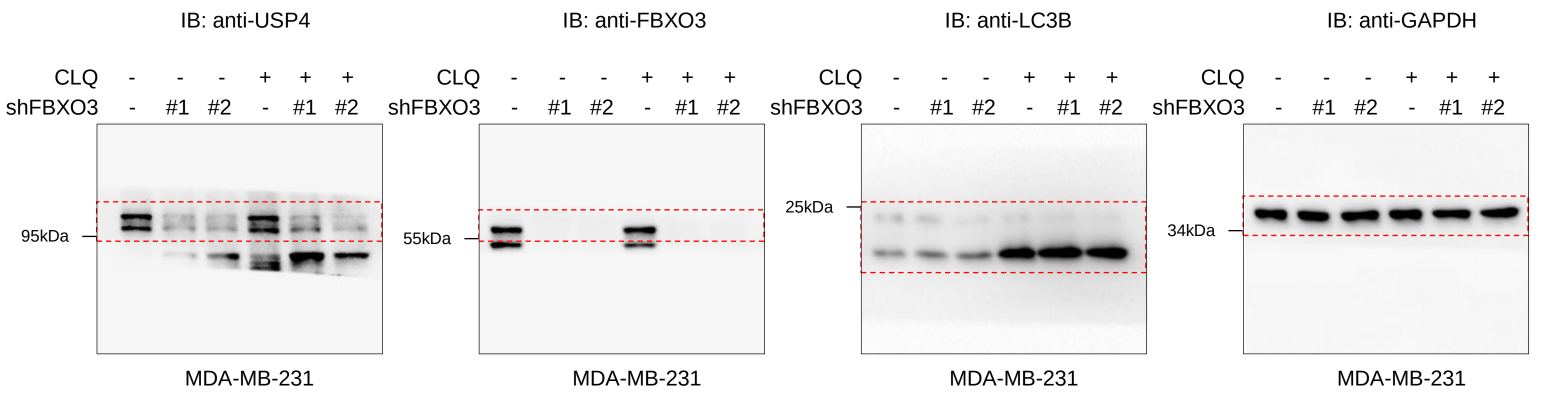

Figure4 B

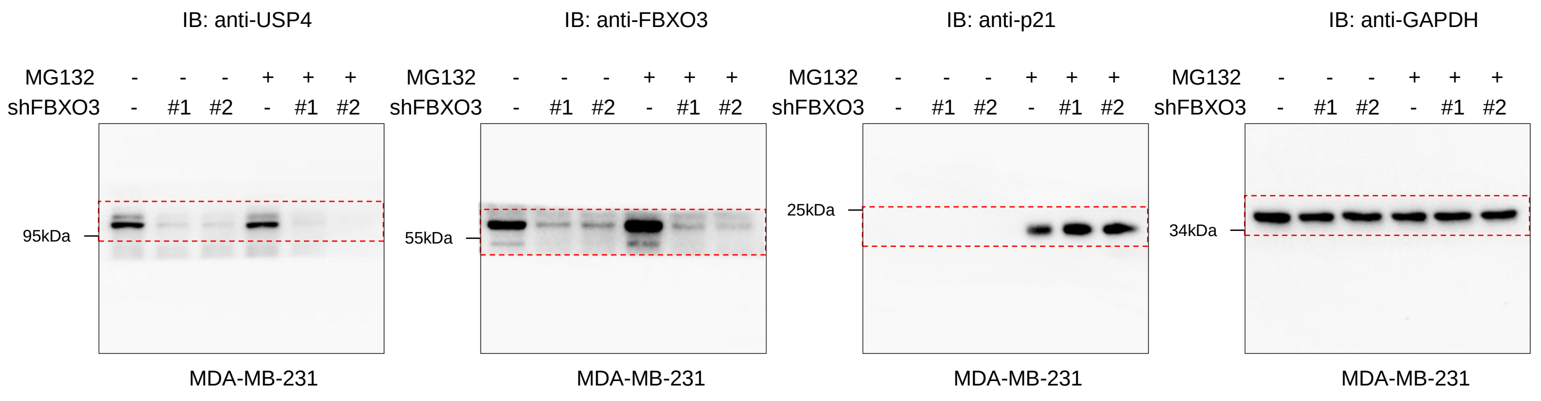

Figure4 C

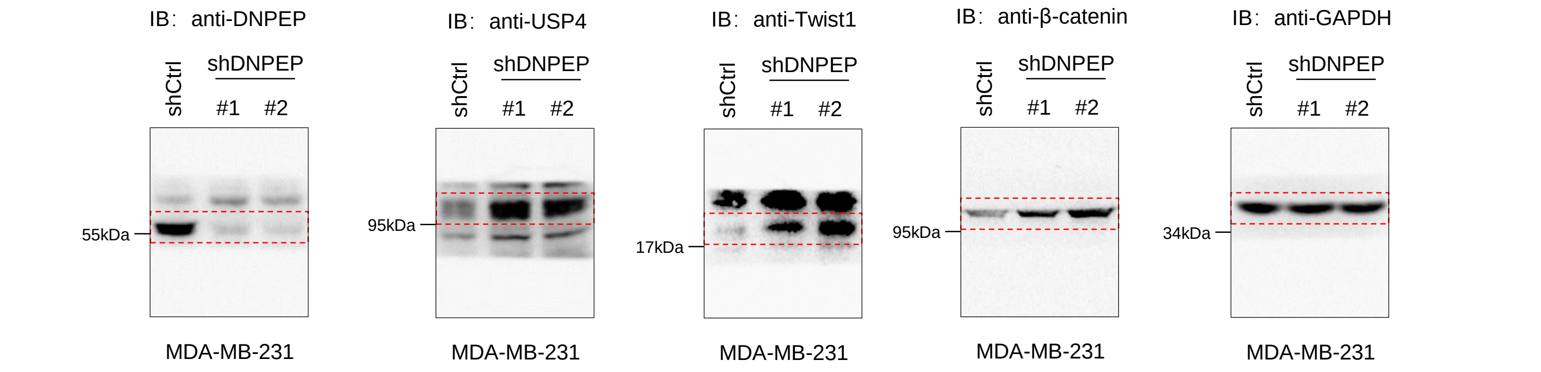

Figure4 D

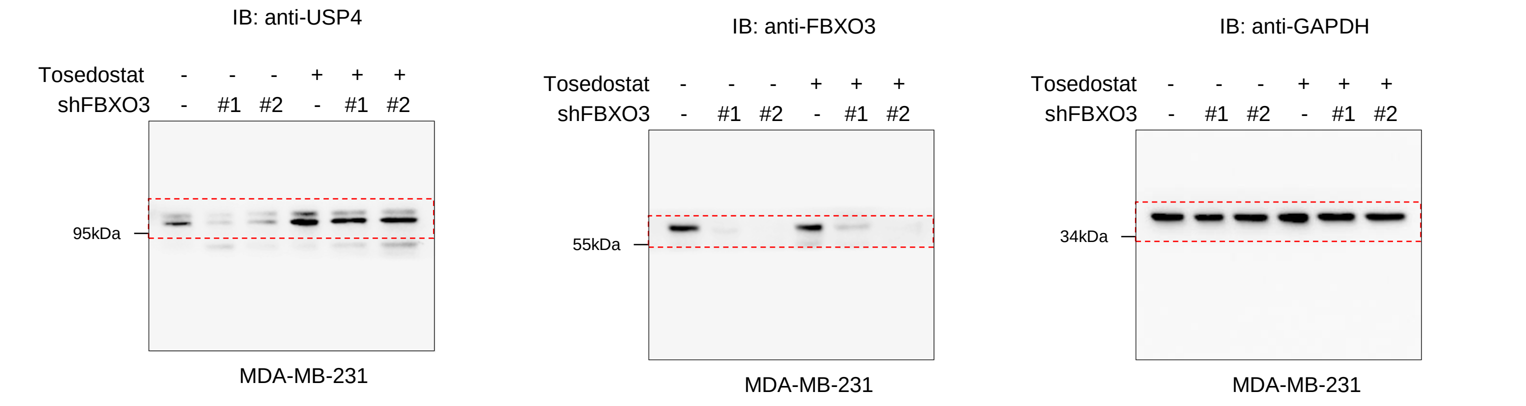

Figure4 E

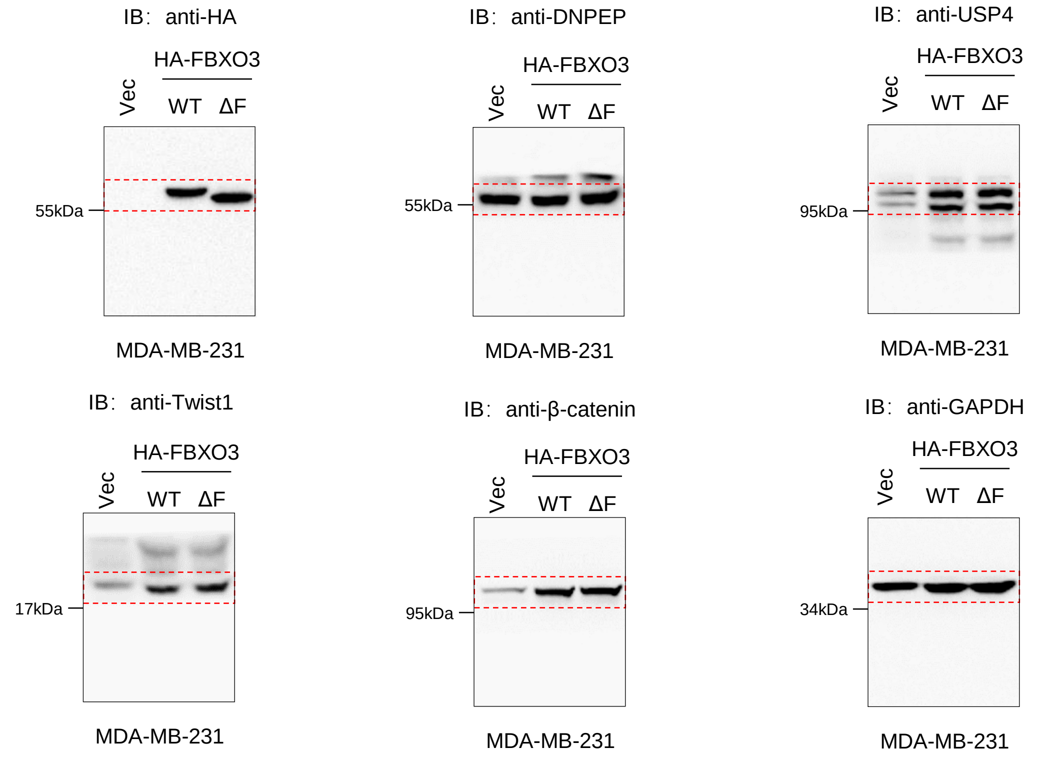

Figure4 F

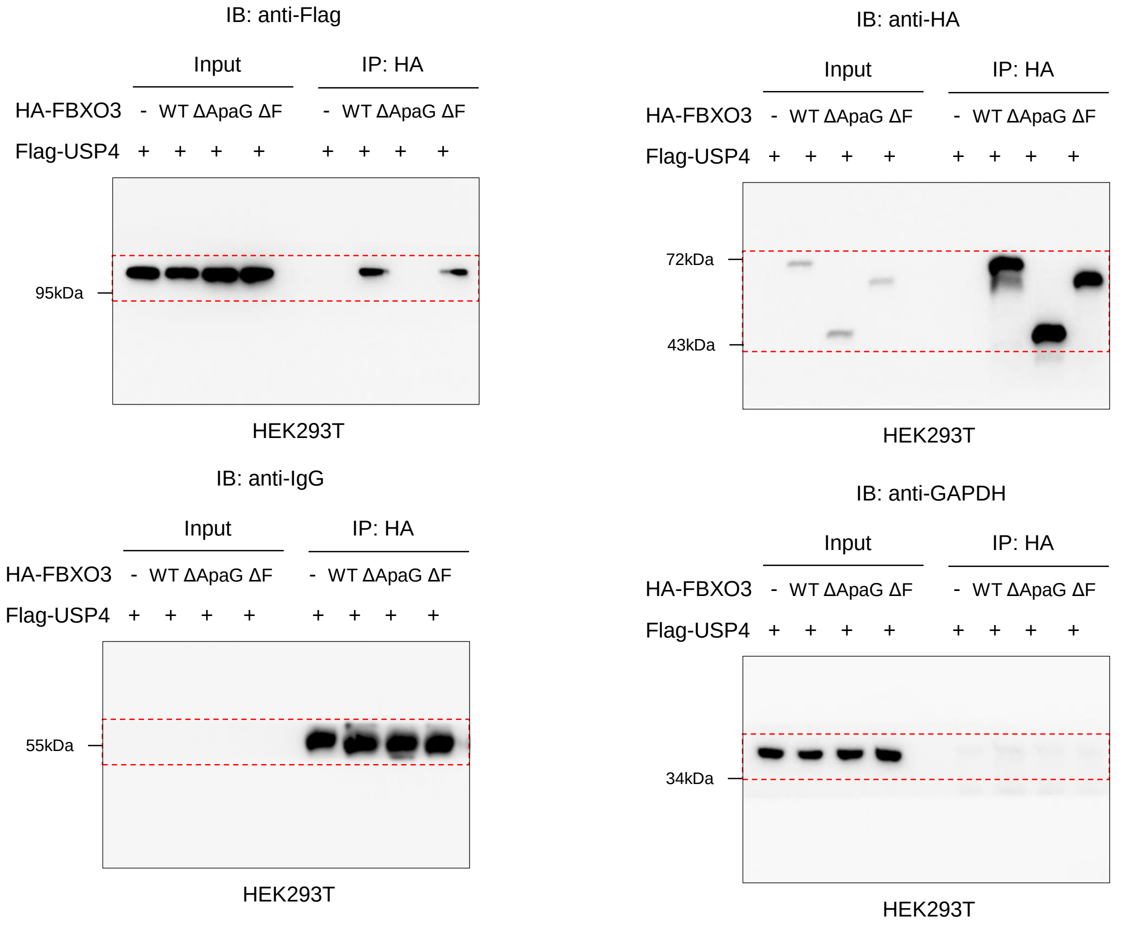

Figure4 G

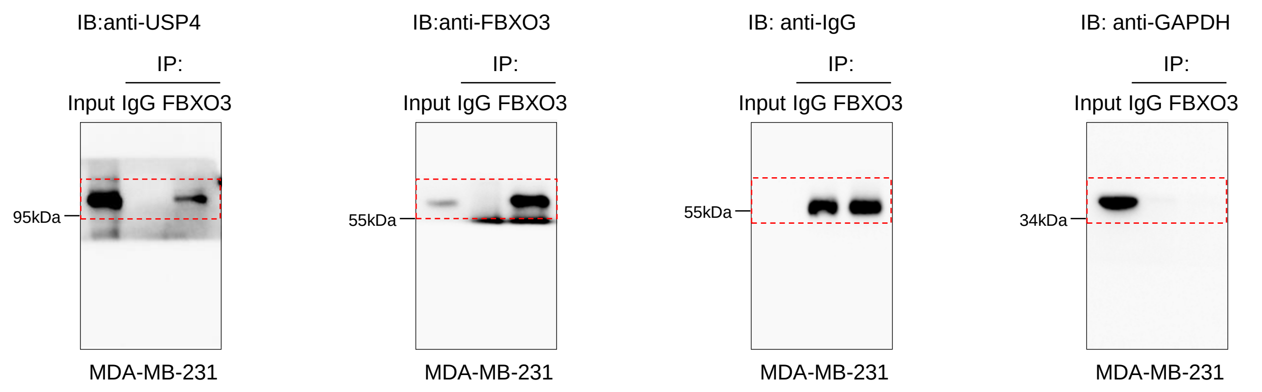

Figure4 H

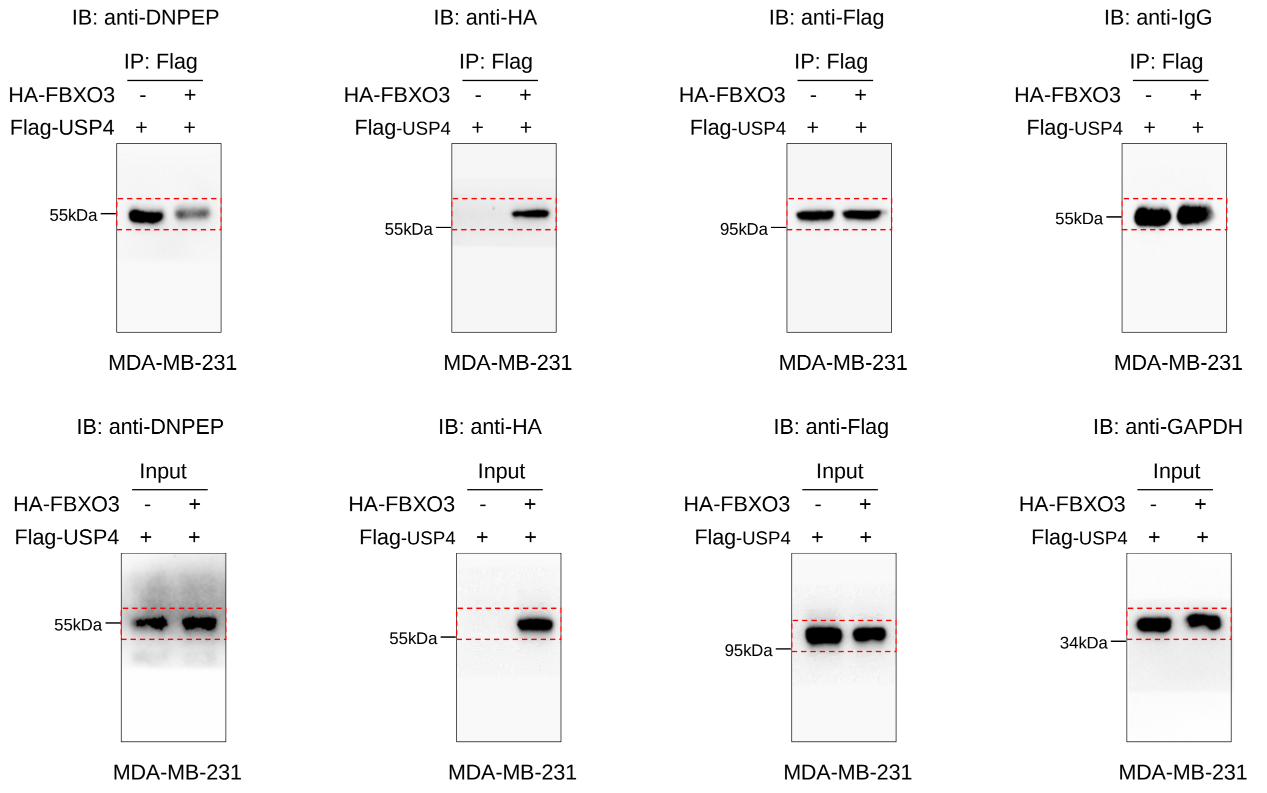

Figure4 I

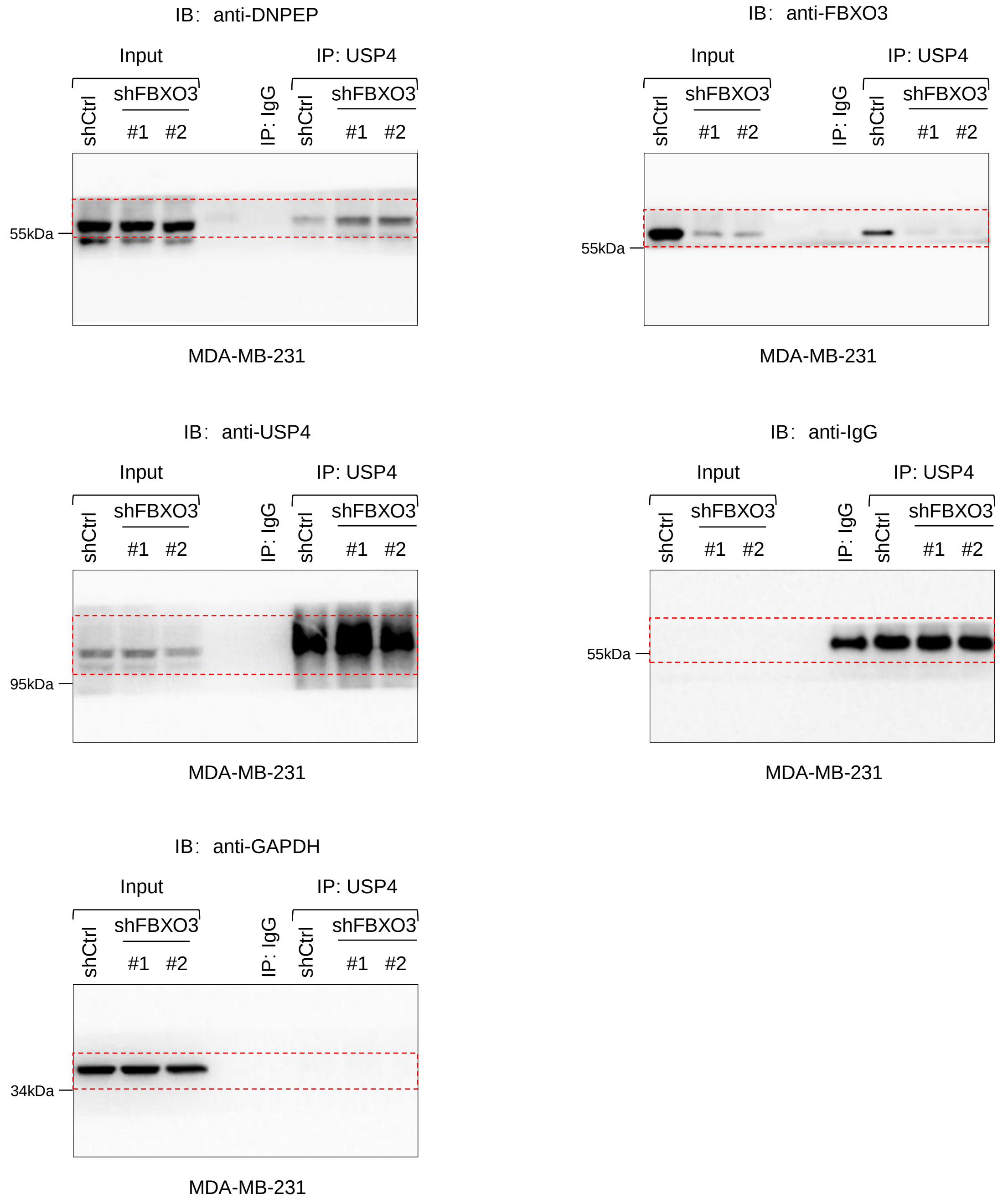
IB: anti-DNPEP
Input
IP: USP4
shFBXO3
shCtrl
#1
#2
IP: IgG
shCtrl
#1
#2
55kDa
MDA-MB-231
IB: anti-FBXO3
55kDa
MDA-MB-231
IB: anti-USP4
95kDa
MDA-MB-231
IB: anti-IgG
55kDa
MDA-MB-231
IB: anti-GAPDH
34kDa
MDA-MB-231

Figure5 A

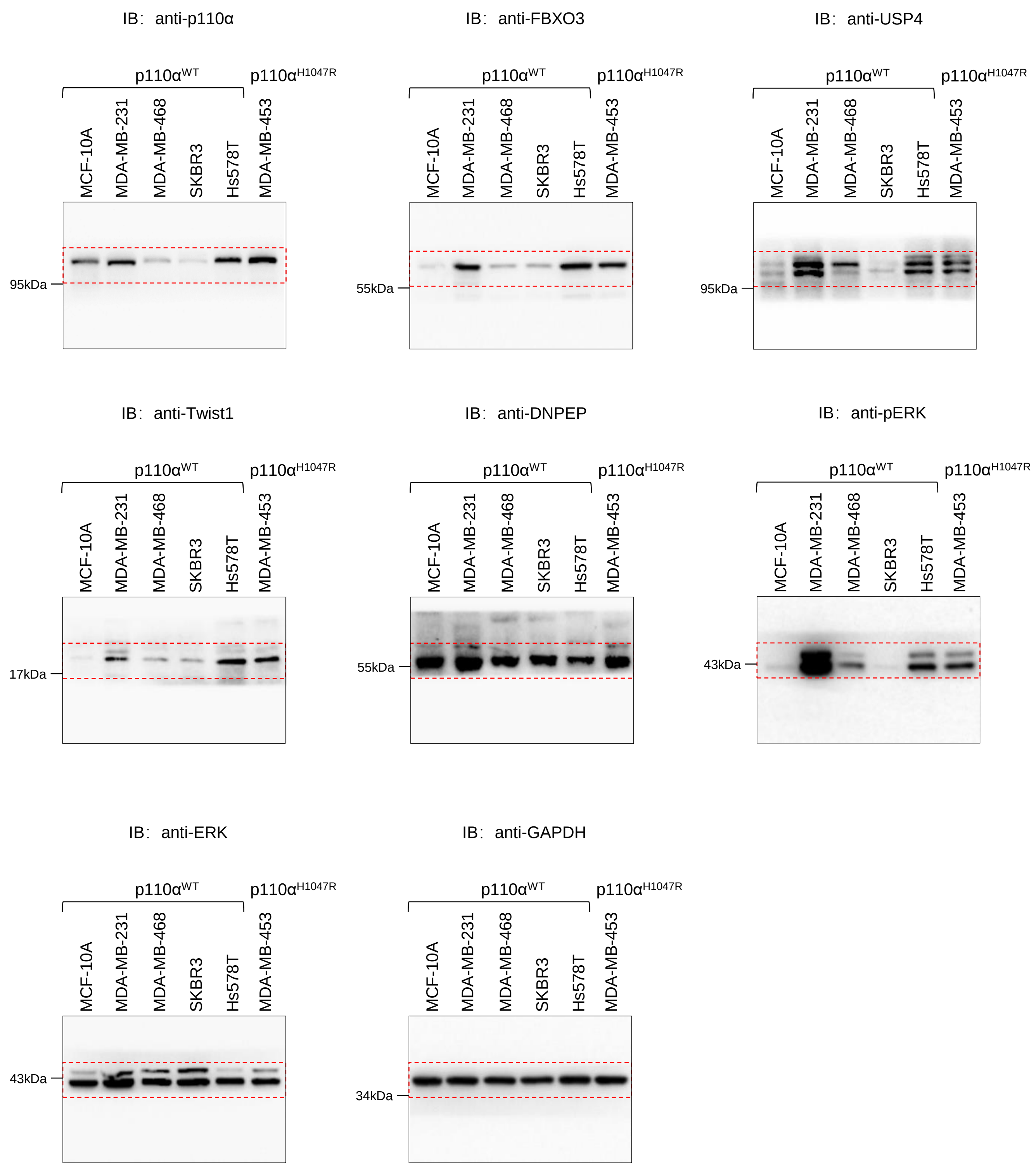
IB: anti-p110α
p110α^WT
p110α^H1047R
MCF-10A
MDA-MB-231
MDA-MB-468
SKBR3
Hs578T
MDA-MB-453
95kDa
IB: anti-FBXO3
55kDa
IB: anti-USP4
95kDa
IB: anti-Twist1
17kDa
IB: anti-DNPEP
55kDa
IB: anti-pERK
43kDa
IB: anti-ERK
43kDa
IB: anti-GAPDH
34kDa

Figure5 B

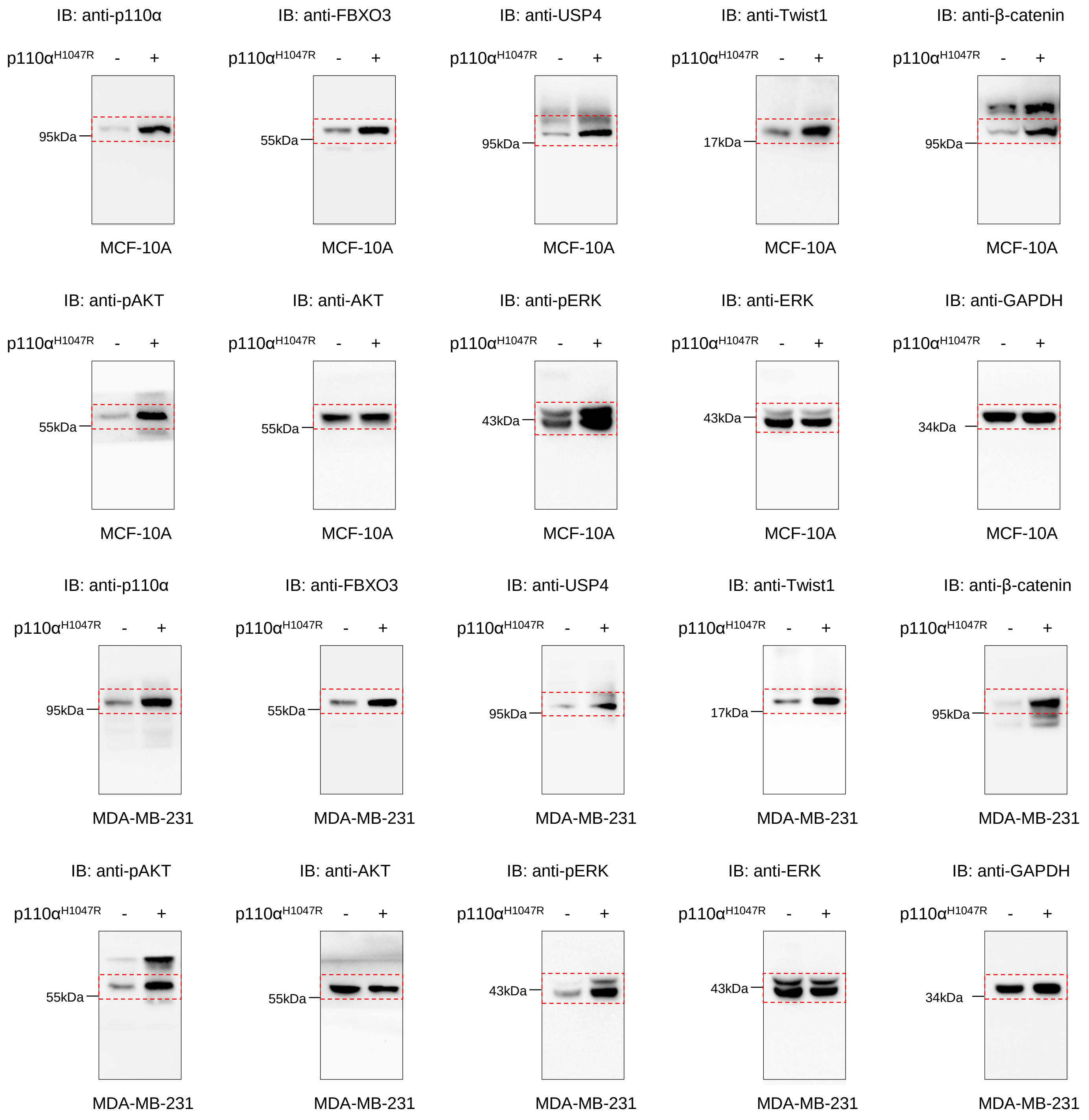

IB: anti-p110α
IB: anti-FBXO3
IB: anti-USP4
IB: anti-Twist1
IB: anti-β-catenin
p110α^{H1047R} - +
95kDa
55kDa
95kDa
17kDa
95kDa
MCF-10A
IB: anti-pAKT
IB: anti-AKT
IB: anti-pERK
IB: anti-ERK
IB: anti-GAPDH
55kDa
55kDa
43kDa
43kDa
34kDa
MCF-10A
IB: anti-p110α
IB: anti-FBXO3
IB: anti-USP4
IB: anti-Twist1
IB: anti-β-catenin
95kDa
55kDa
95kDa
17kDa
95kDa
MDA-MB-231
IB: anti-pAKT
IB: anti-AKT
IB: anti-pERK
IB: anti-ERK
IB: anti-GAPDH
55kDa
55kDa
43kDa
43kDa
34kDa
MDA-MB-231

Figure5 C

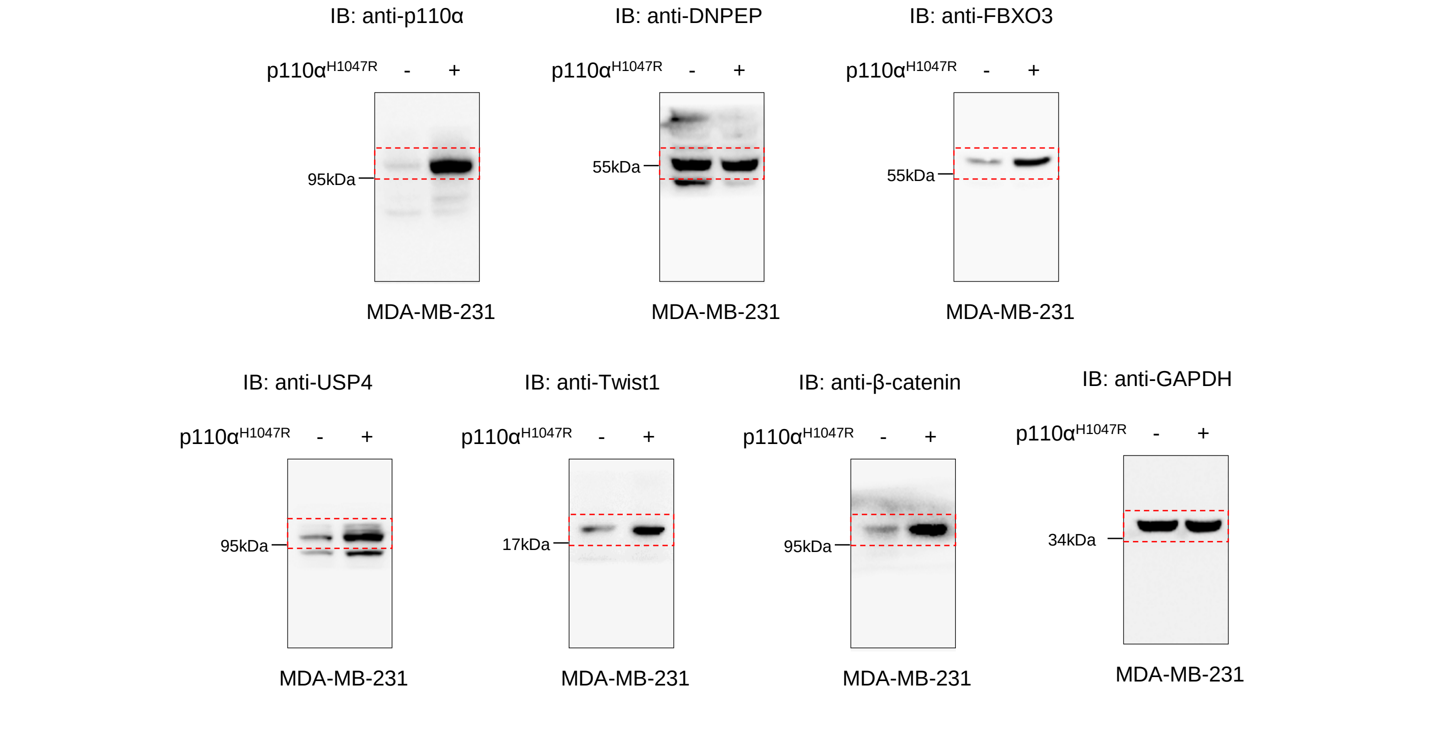

Figure5 D

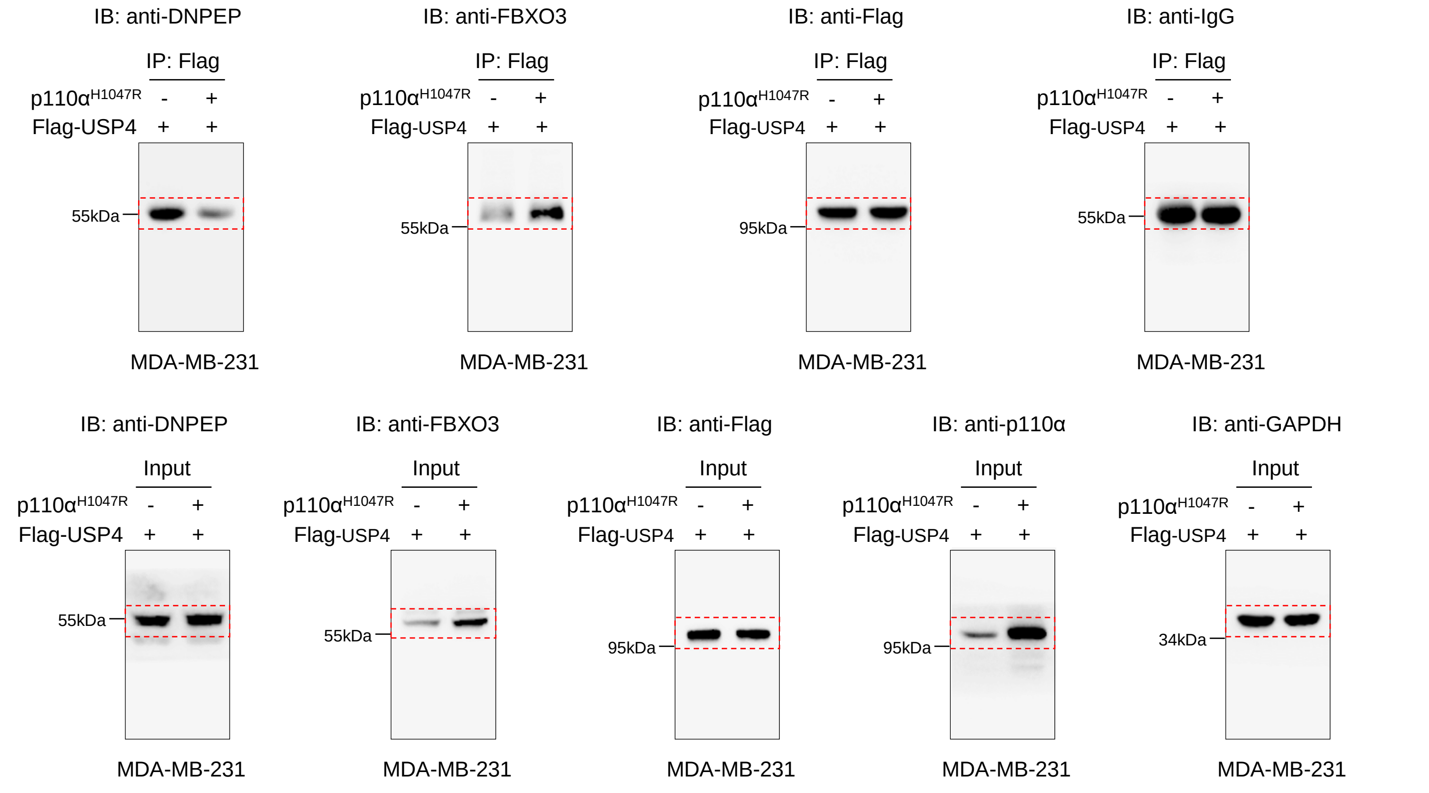

Figure5 E

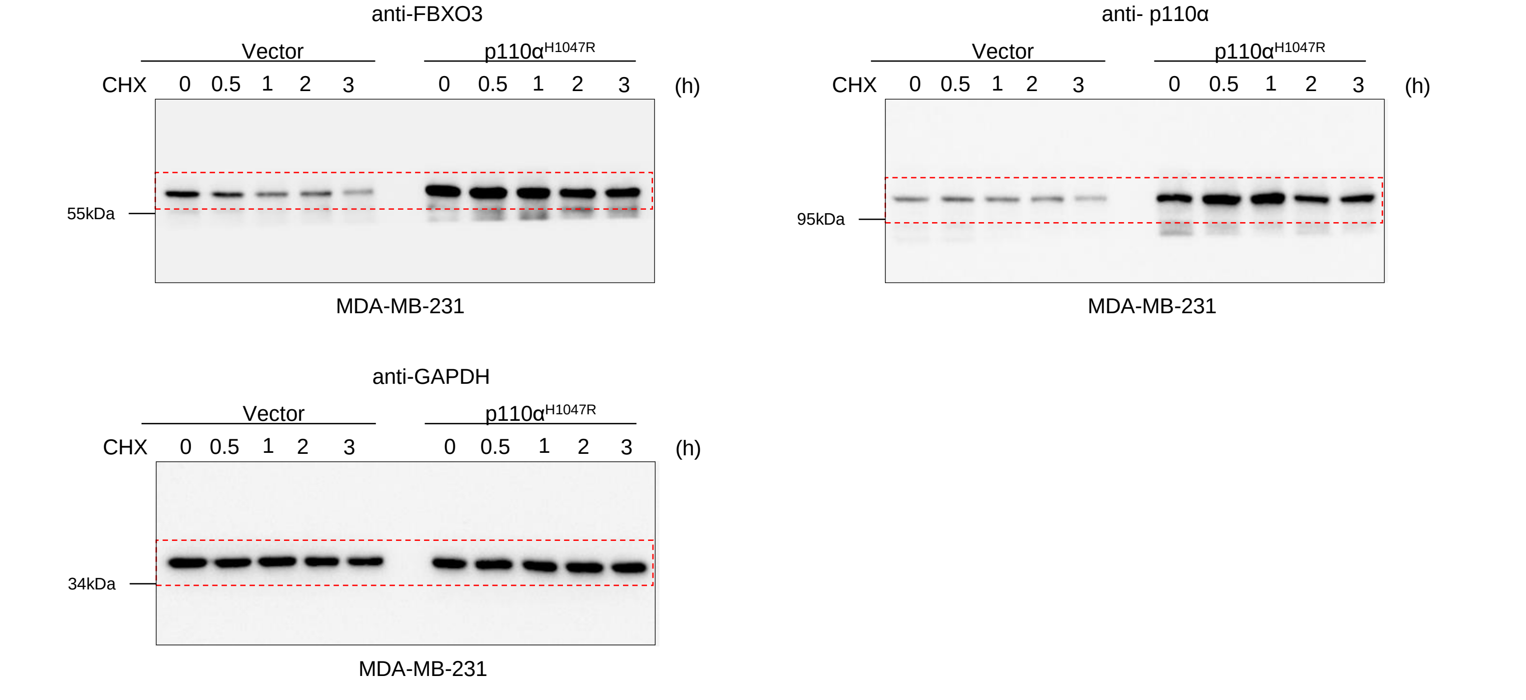

Figure5 F

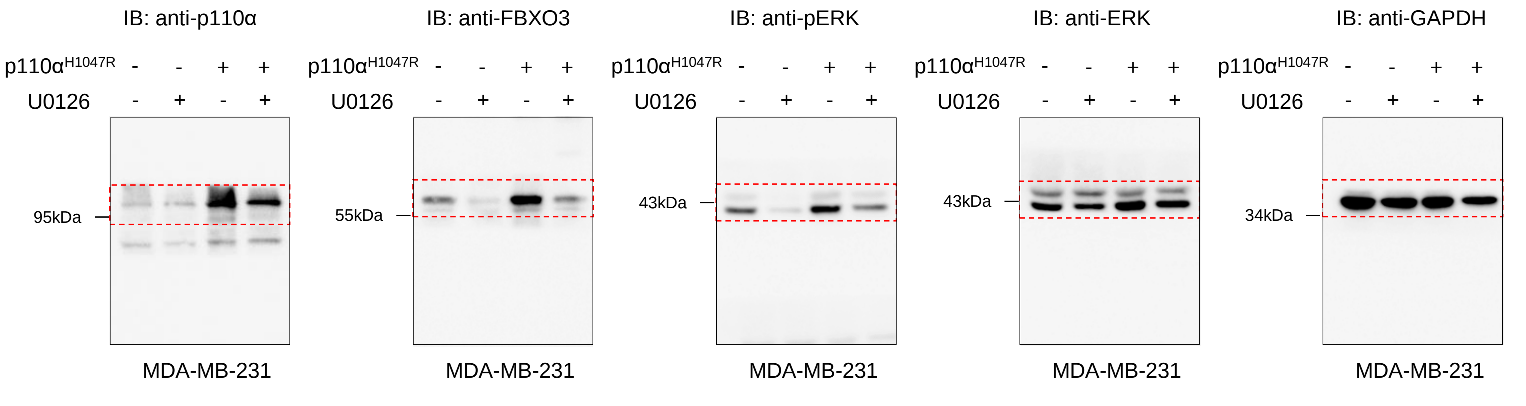

Figure5 G

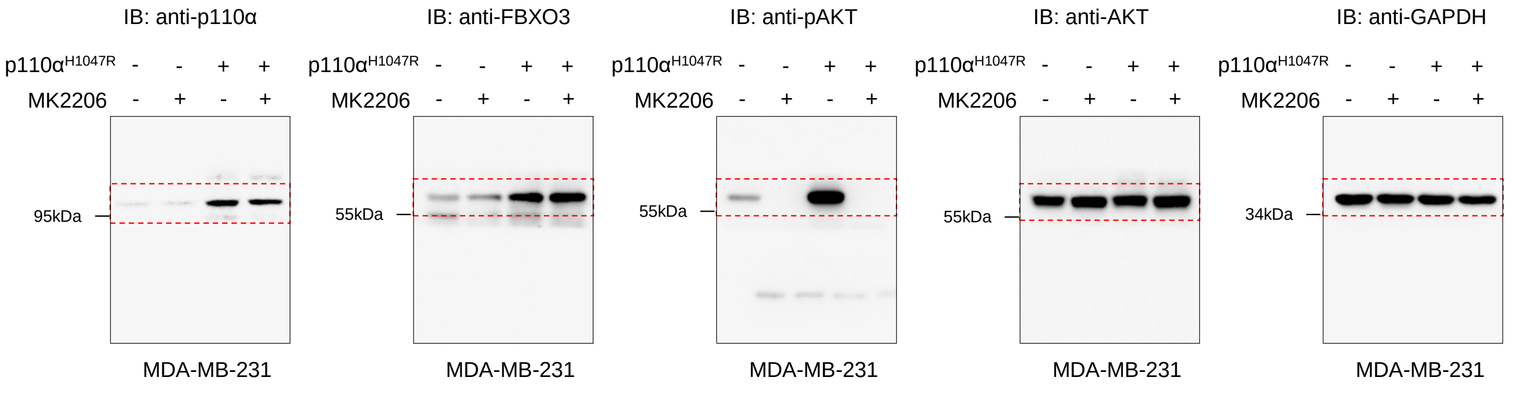

Figure5 H

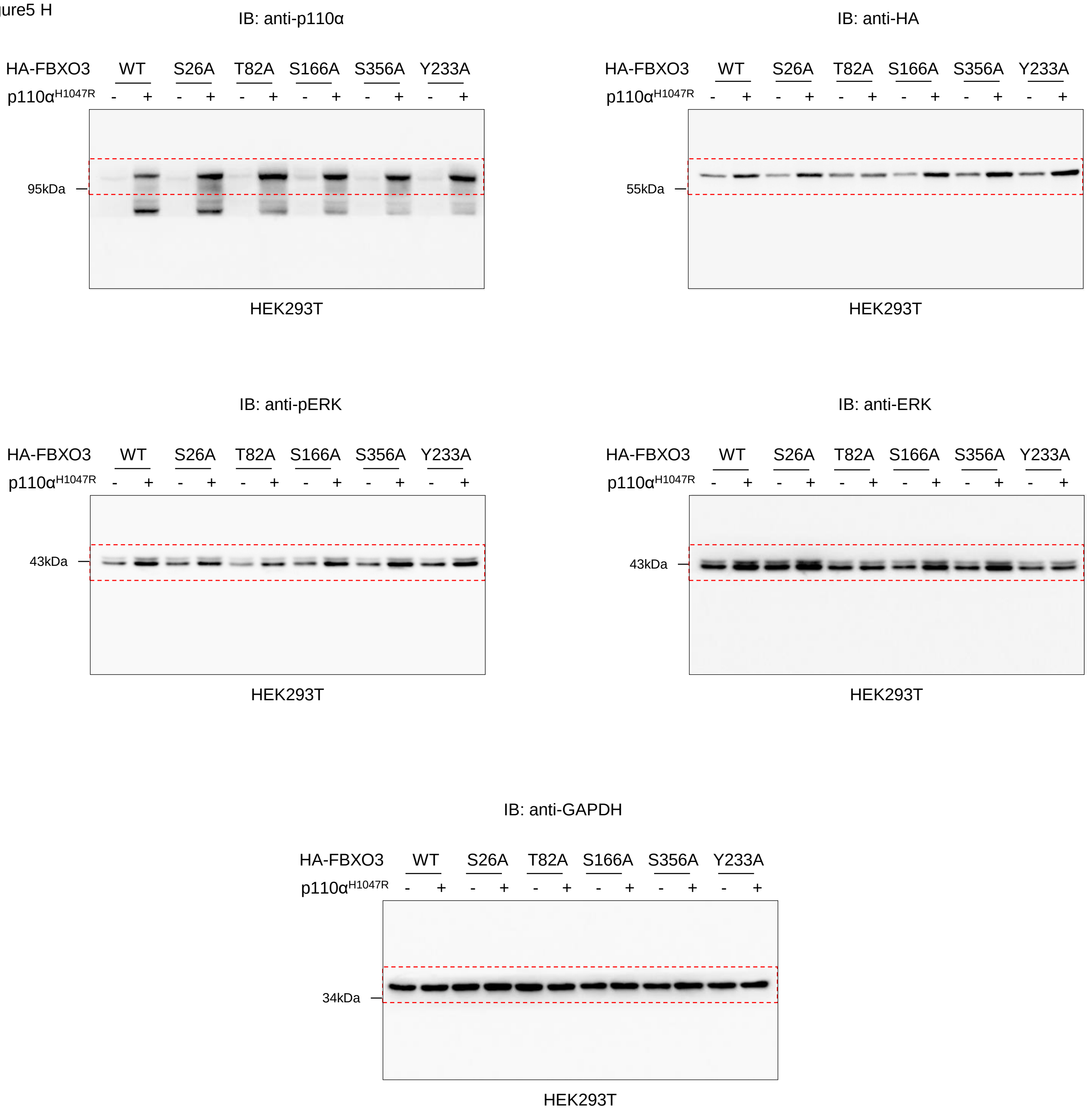
IB: anti-p110α
HA-FBXO3 WT S26A T82A S166A S356A Y233A
p110α H1047R - + - + - + - + - + - +
95kDa
HEK293T
IB: anti-HA
HA-FBXO3 WT S26A T82A S166A S356A Y233A
p110α H1047R - + - + - + - + - + - +
55kDa
HEK293T
IB: anti-pERK
HA-FBXO3 WT S26A T82A S166A S356A Y233A
p110α H1047R - + - + - + - + - + - +
43kDa
HEK293T
IB: anti-ERK
HA-FBXO3 WT S26A T82A S166A S356A Y233A
p110α H1047R - + - + - + - + - + - +
43kDa
HEK293T
IB: anti-GAPDH
HA-FBXO3 WT S26A T82A S166A S356A Y233A
p110α H1047R - + - + - + - + - + - +
34kDa
HEK293T

Figure5 J

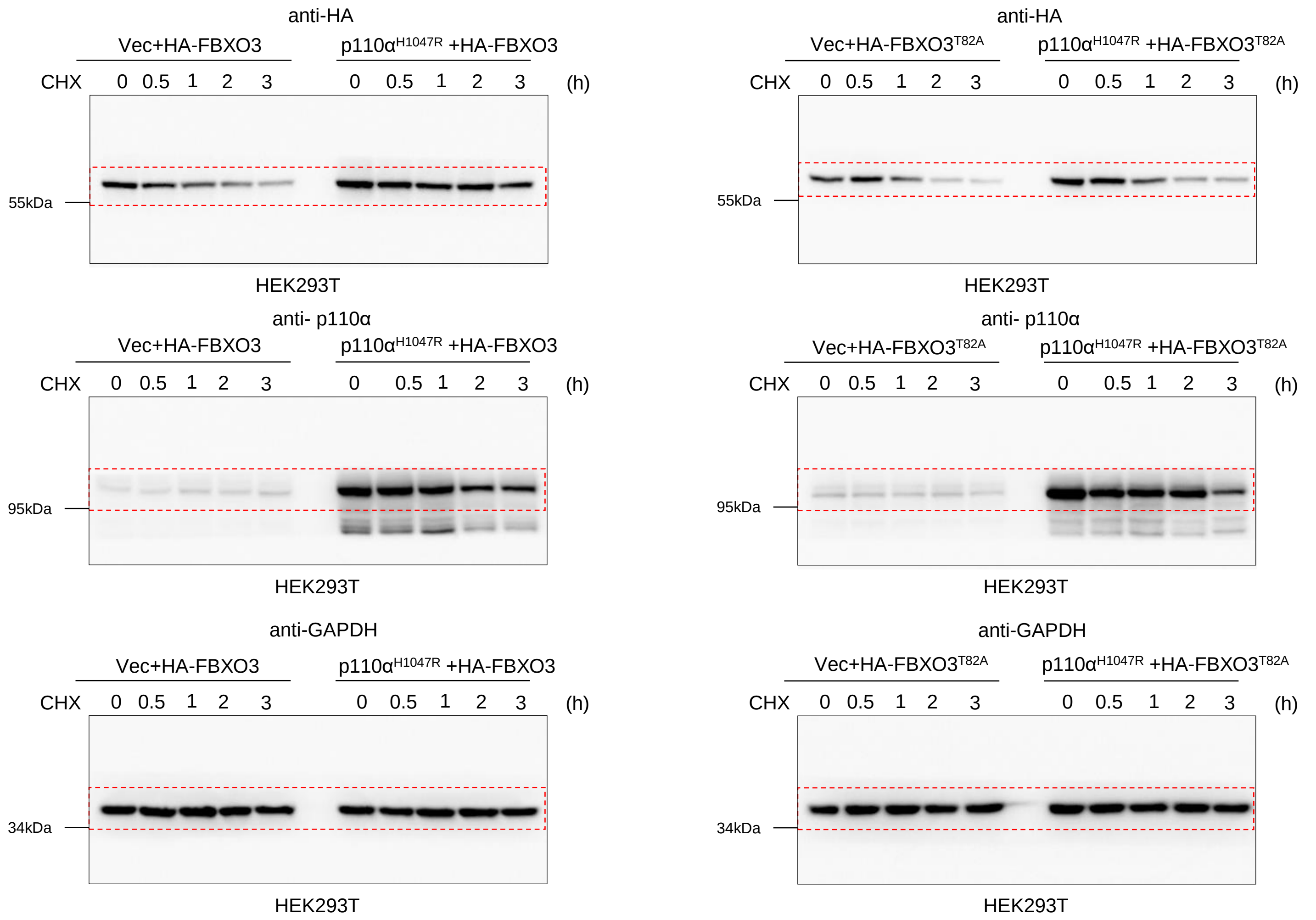

Figure5 L

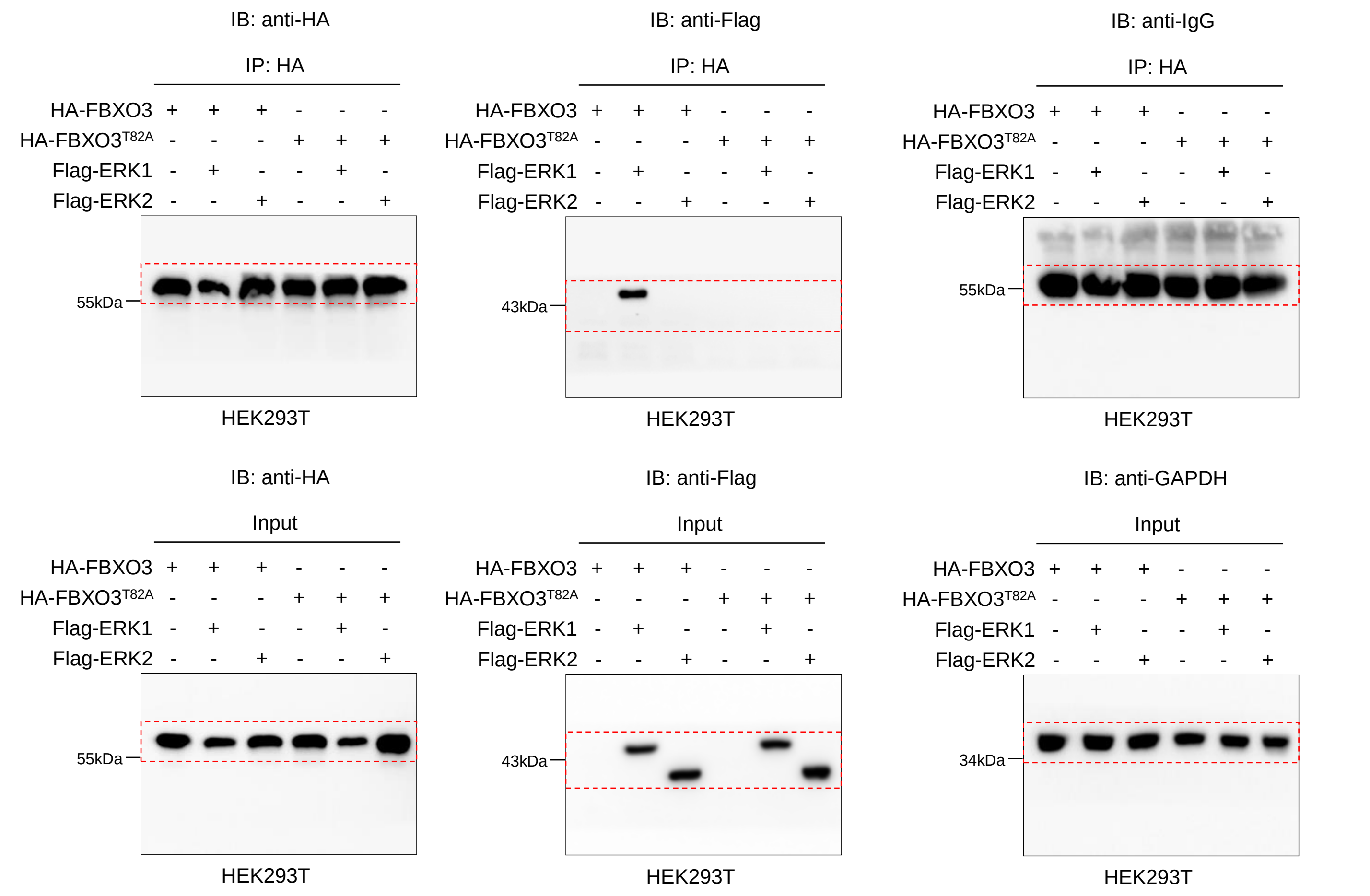

Figure6 A

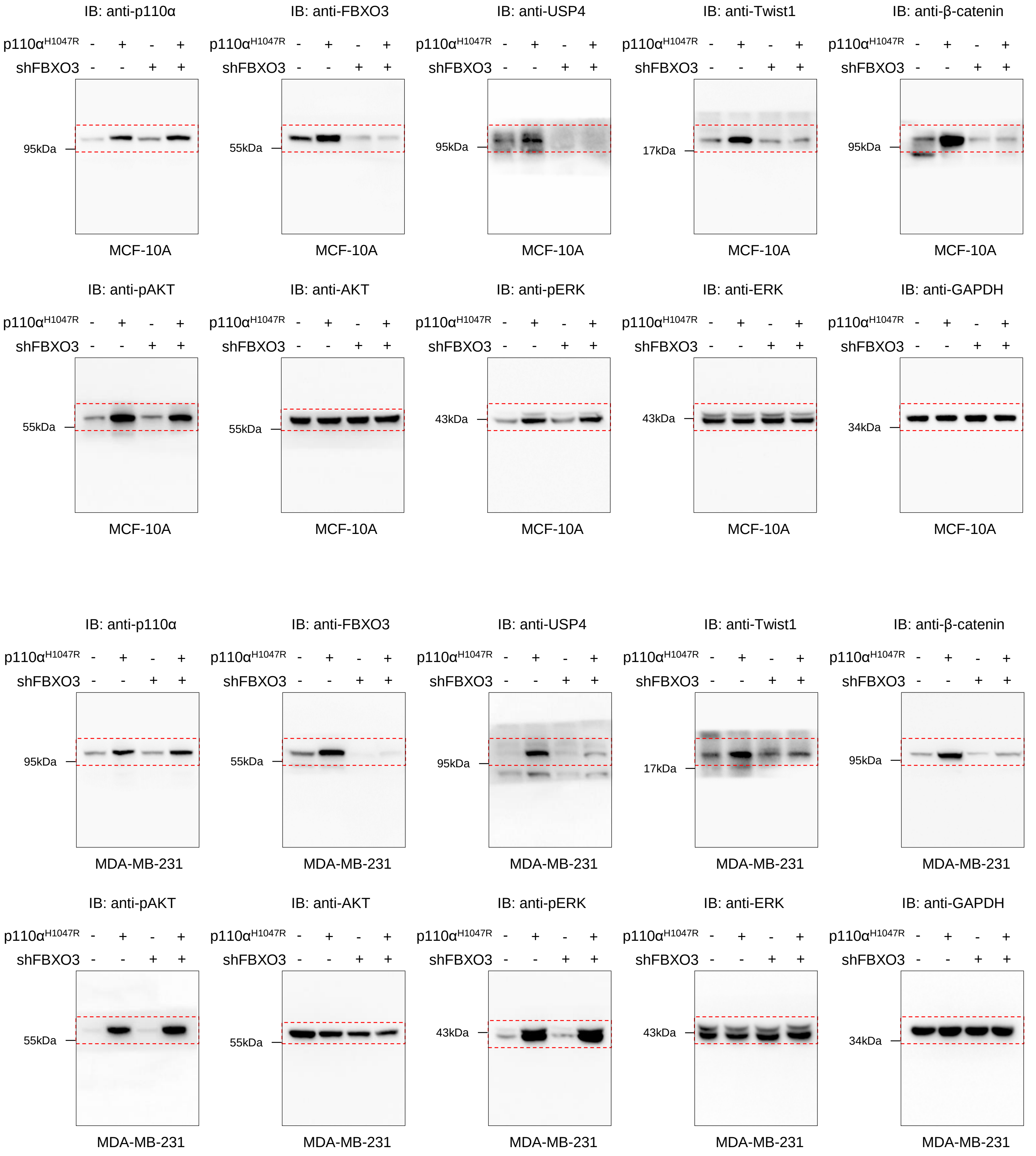
IB: anti-p110α
IB: anti-FBXO3
IB: anti-USP4
IB: anti-Twist1
IB: anti-β-catenin
p110α^{H1047R} - + - +
shFBXO3 - - + +
95kDa
55kDa
95kDa
17kDa
95kDa
MCF-10A
IB: anti-pAKT
IB: anti-AKT
IB: anti-pERK
IB: anti-ERK
IB: anti-GAPDH
p110α^{H1047R} - + - +
shFBXO3 - - + +
55kDa
55kDa
43kDa
43kDa
34kDa
MCF-10A
IB: anti-p110α
IB: anti-FBXO3
IB: anti-USP4
IB: anti-Twist1
IB: anti-β-catenin
p110α^{H1047R} - + - +
shFBXO3 - - + +
95kDa
55kDa
95kDa
17kDa
95kDa
MDA-MB-231
IB: anti-pAKT
IB: anti-AKT
IB: anti-pERK
IB: anti-ERK
IB: anti-GAPDH
p110α^{H1047R} - + - +
shFBXO3 - - + +
55kDa
55kDa
43kDa
43kDa
34kDa
MDA-MB-231

Figure6 F

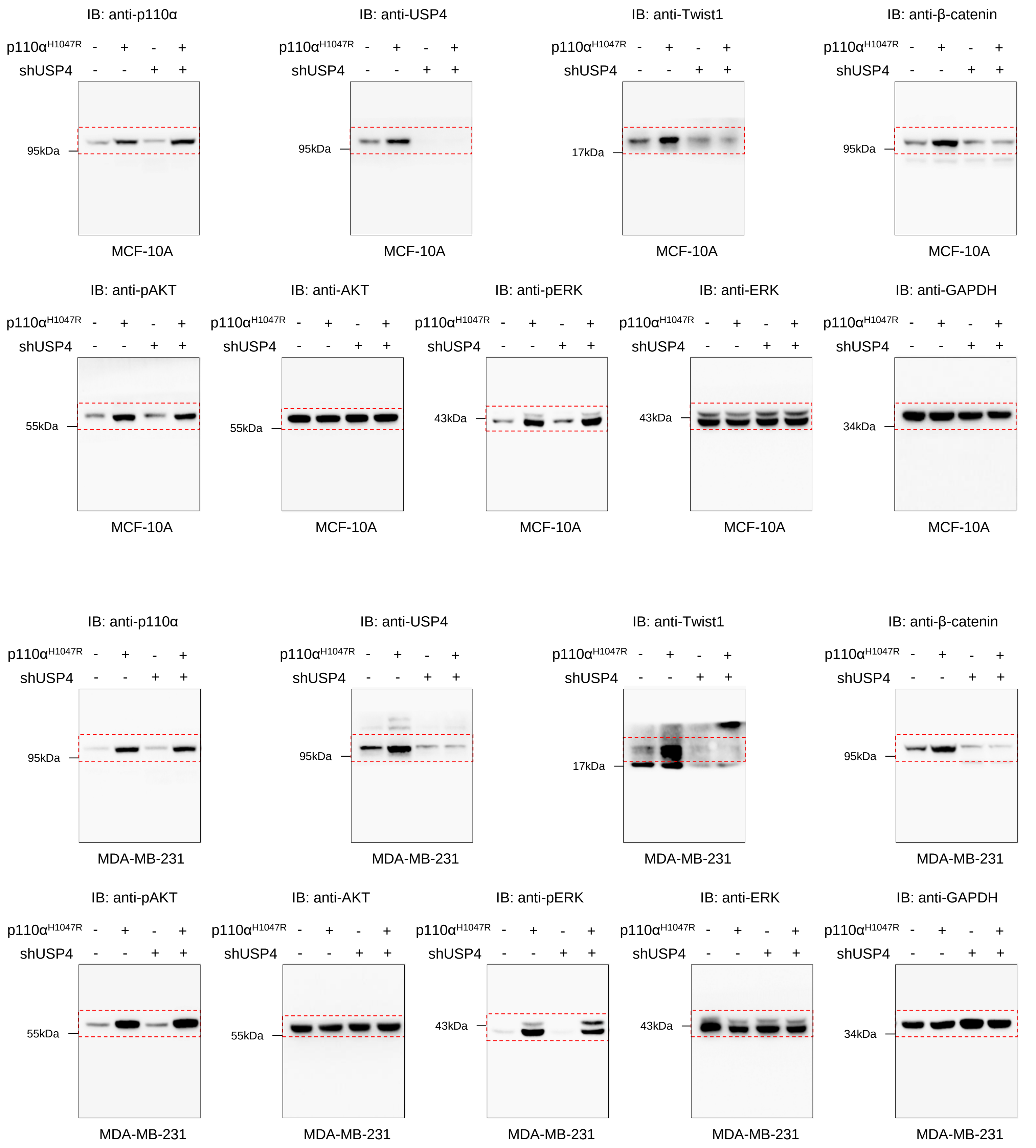
IB: anti-p110α
IB: anti-USP4
IB: anti-Twist1
IB: anti-β-catenin
p110α^{H1047R} - + - +
shUSP4 - - + +
95kDa
17kDa
MCF-10A
IB: anti-pAKT
IB: anti-AKT
IB: anti-pERK
IB: anti-ERK
IB: anti-GAPDH
55kDa
43kDa
34kDa
MCF-10A
IB: anti-p110α
IB: anti-USP4
IB: anti-Twist1
IB: anti-β-catenin
95kDa
17kDa
MDA-MB-231
IB: anti-pAKT
IB: anti-AKT
IB: anti-pERK
IB: anti-ERK
IB: anti-GAPDH
55kDa
43kDa
34kDa
MDA-MB-231

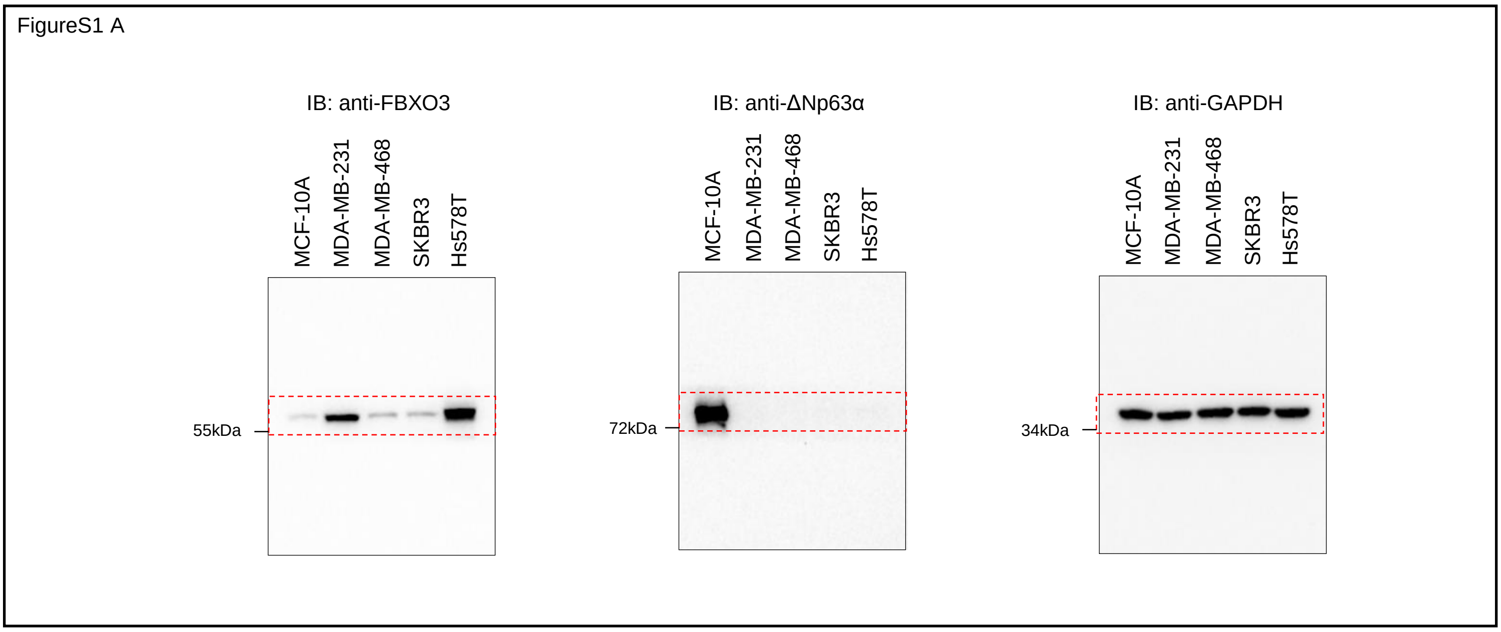
FigureS1 A
IB: anti-FBXO3
IB: anti-ΔNp63α
IB: anti-GAPDH
MCF-10A
MDA-MB-231
MDA-MB-468
SKBR3
Hs578T
55kDa
72kDa
34kDa

FigureS2 B

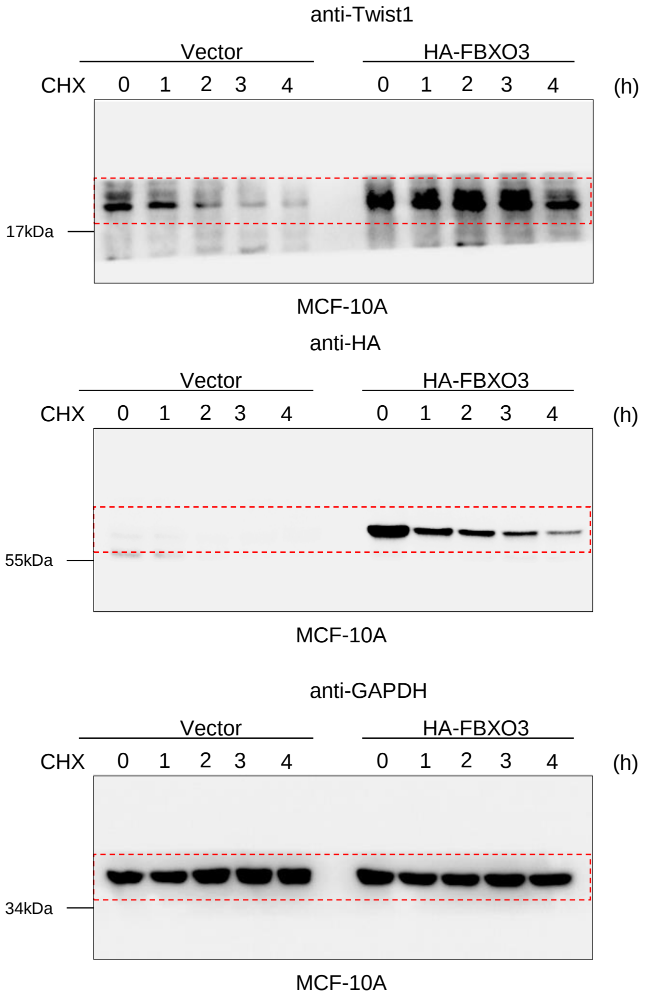

FigureS2 D

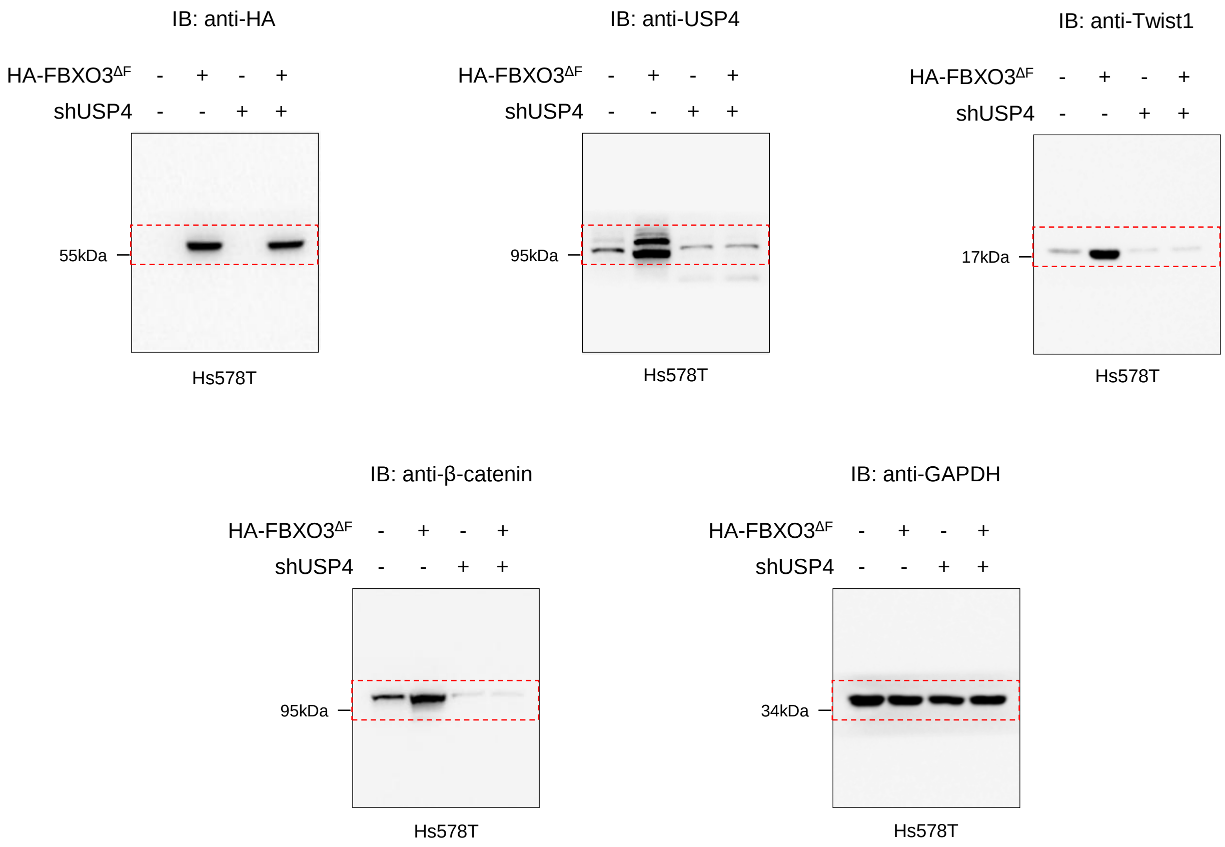

FigureS3 C

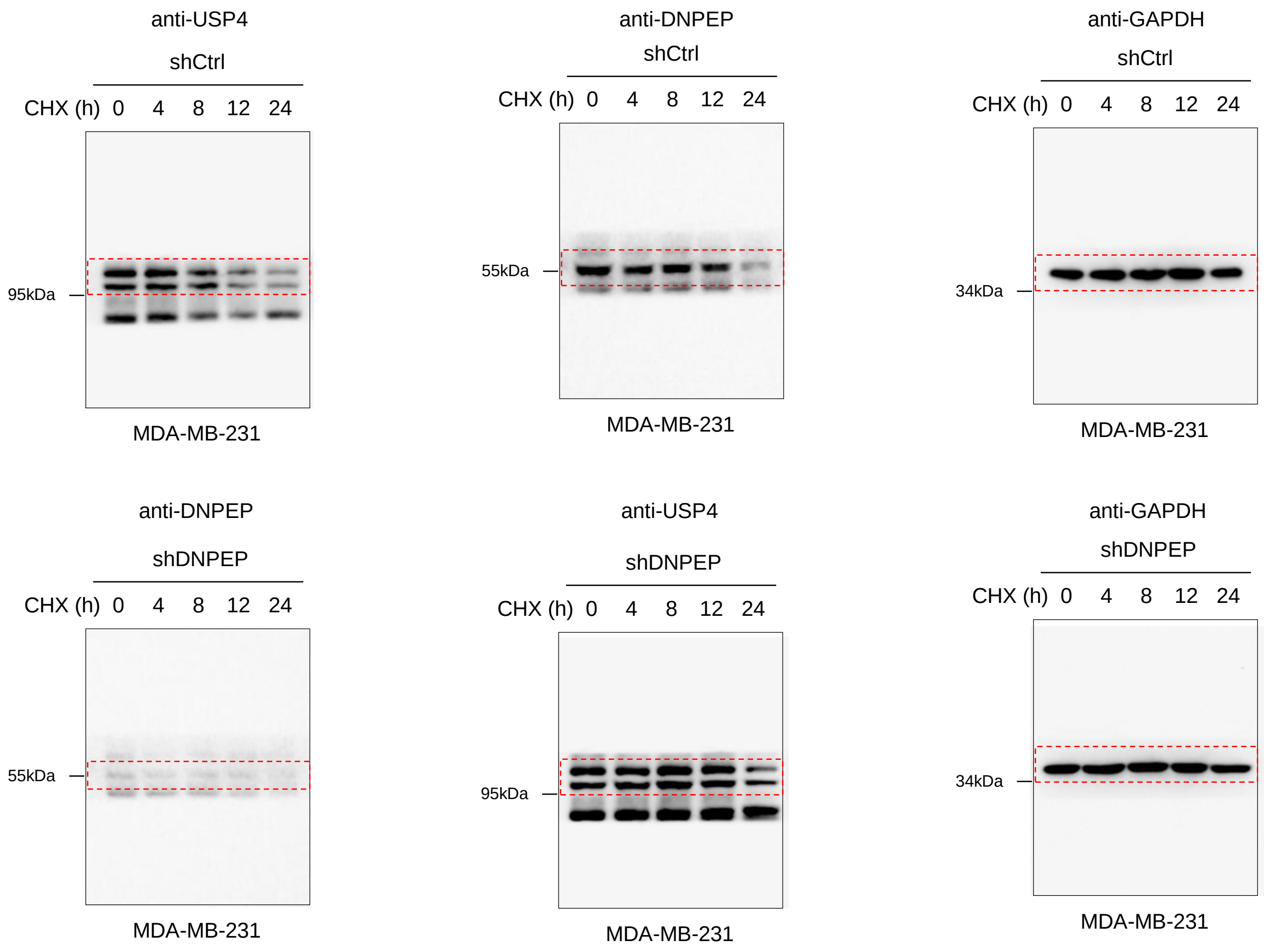
anti-USP4
shCtrl
CHX (h) 0 4 8 12 24
95kDa
MDA-MB-231
anti-DNPEP
shCtrl
CHX (h) 0 4 8 12 24
55kDa
MDA-MB-231
anti-GAPDH
shCtrl
CHX (h) 0 4 8 12 24
34kDa
MDA-MB-231
anti-DNPEP
shDNPEP
CHX (h) 0 4 8 12 24
55kDa
MDA-MB-231
anti-USP4
shDNPEP
CHX (h) 0 4 8 12 24
95kDa
MDA-MB-231
anti-GAPDH
shDNPEP
CHX (h) 0 4 8 12 24
34kDa
MDA-MB-231

FigureS4 B

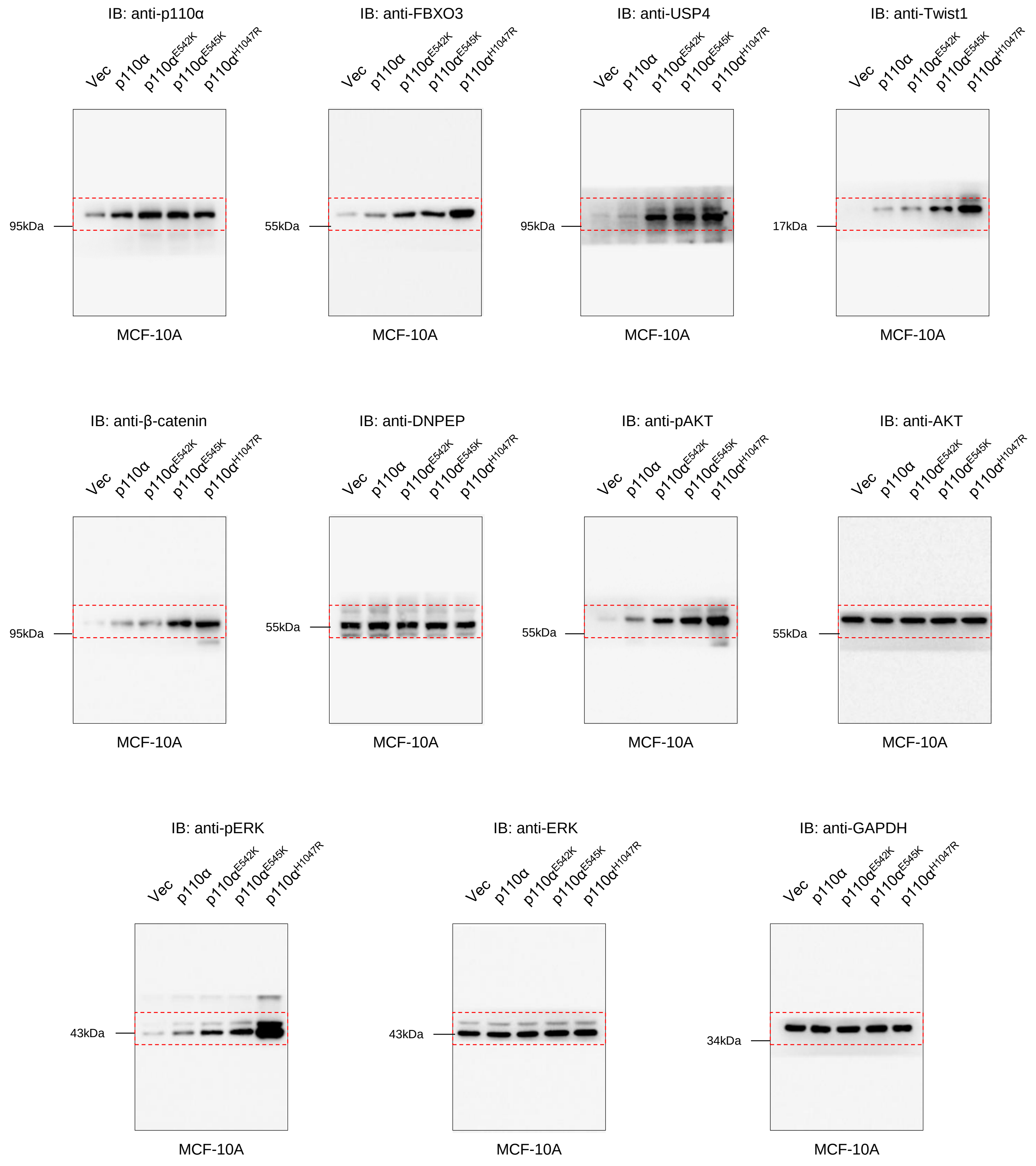
IB: anti-p110α
IB: anti-FBXO3
IB: anti-USP4
IB: anti-Twist1
IB: anti-β-catenin
IB: anti-DNPEP
IB: anti-pAKT
IB: anti-AKT
IB: anti-pERK
IB: anti-ERK
IB: anti-GAPDH
Vec
p110α
p110α^{E542K}
p110α^{E545K}
p110α^{H1047R}
95kDa
55kDa
17kDa
43kDa
34kDa
MCF-10A

FigureS4 E

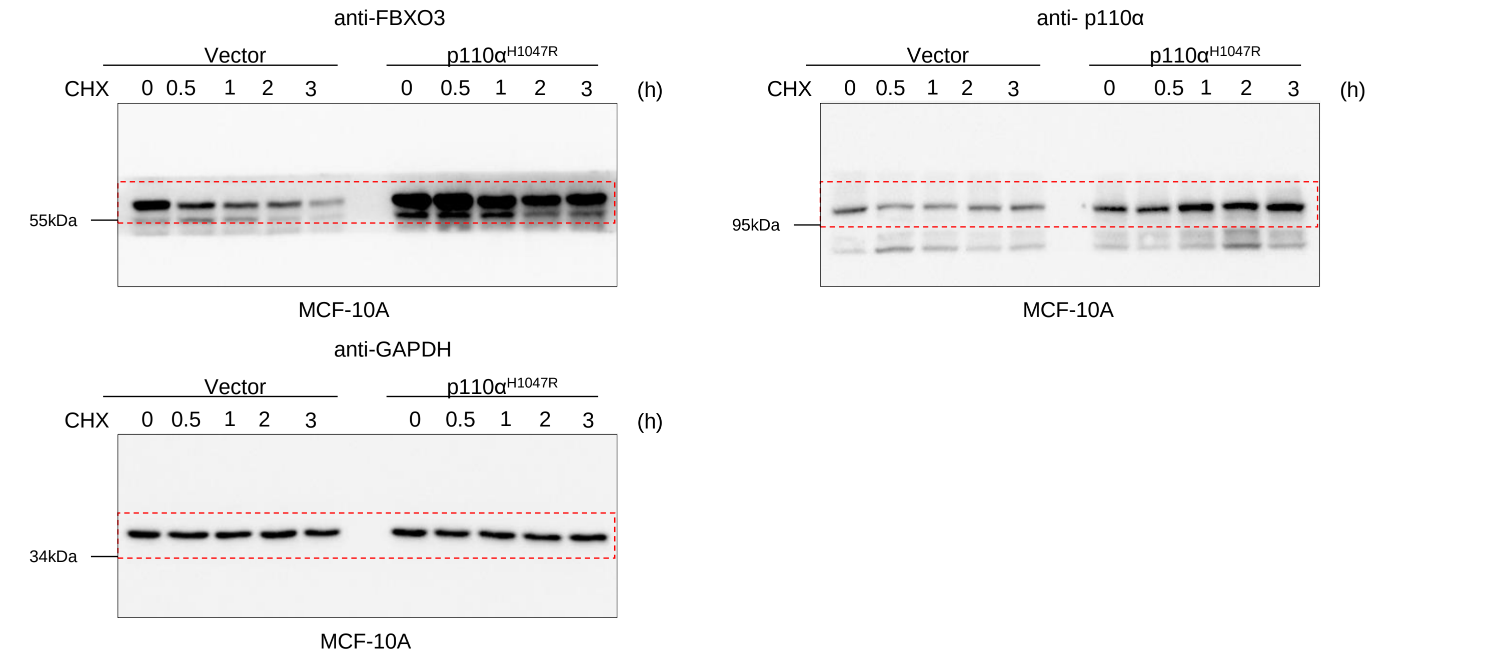

FigureS4 F

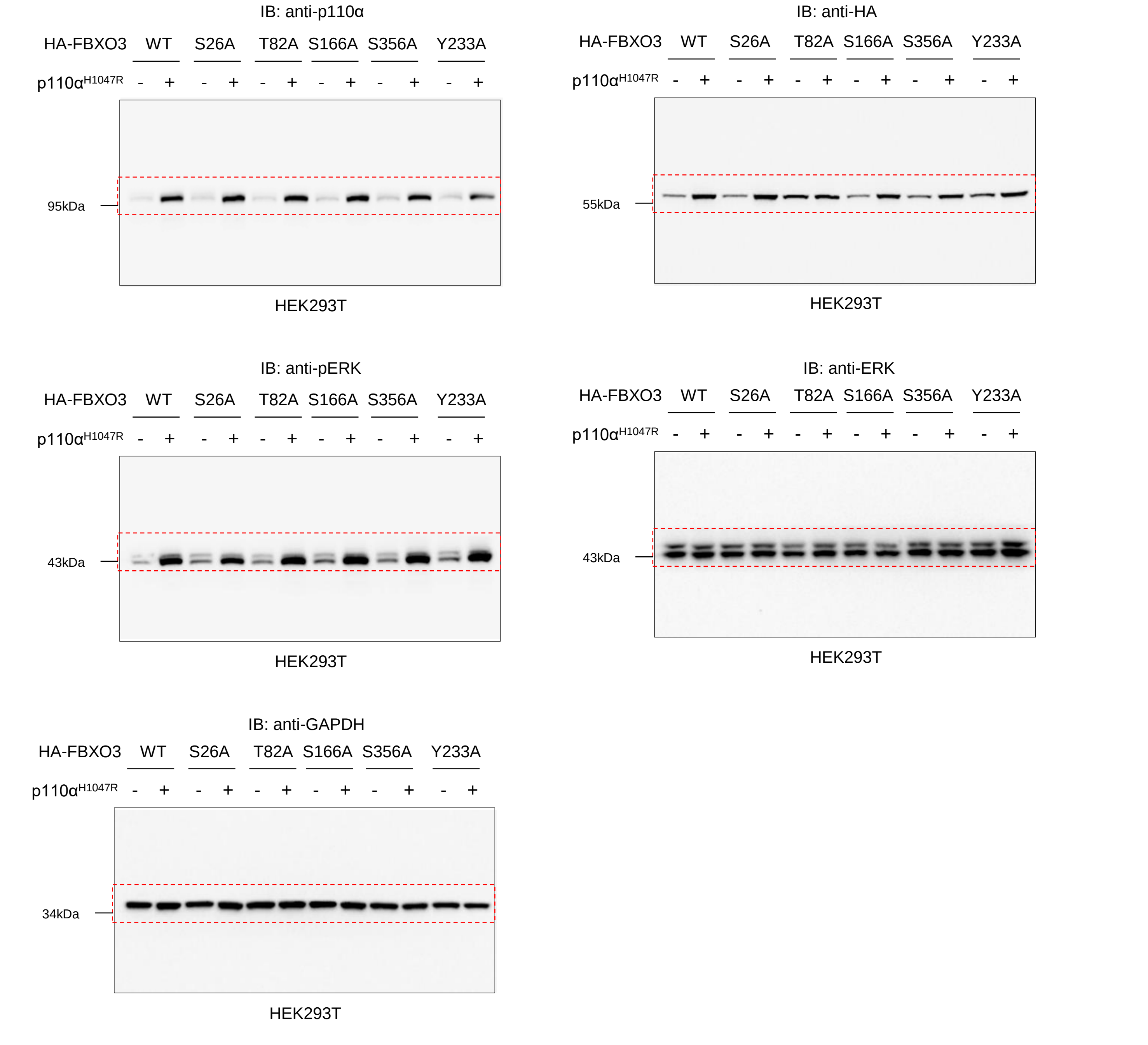

FigureS4 G

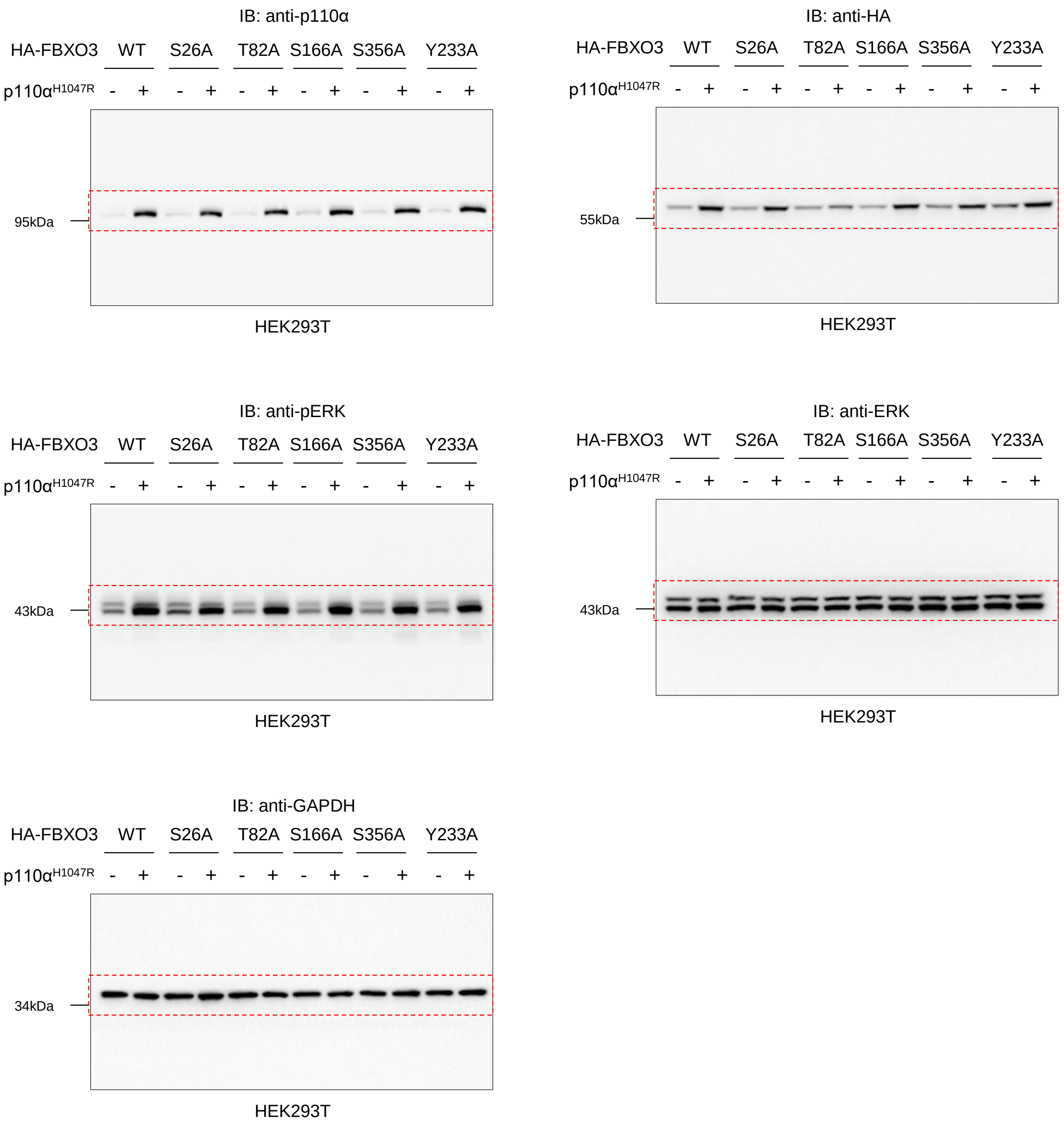
IB: anti-p110α
HA-FBXO3 WT S26A T82A S166A S356A Y233A
p110α H1047R - + - + - + - + - + - +
95kDa
HEK293T
IB: anti-HA
HA-FBXO3 WT S26A T82A S166A S356A Y233A
p110α H1047R - + - + - + - + - + - +
55kDa
HEK293T
IB: anti-pERK
HA-FBXO3 WT S26A T82A S166A S356A Y233A
p110α H1047R - + - + - + - + - + - +
43kDa
HEK293T
IB: anti-ERK
HA-FBXO3 WT S26A T82A S166A S356A Y233A
p110α H1047R - + - + - + - + - + - +
43kDa
HEK293T
IB: anti-GAPDH
HA-FBXO3 WT S26A T82A S166A S356A Y233A
p110α H1047R - + - + - + - + - + - +
34kDa
HEK293T
