# Supplementary figures and images for: FBXO3 stabilizes USP4 and Twist1 to promote PI3K-mediated breast cancer metastasis
Source: PLoS Biol. 2023 Dec 22;21(12):e3002446. doi: 10.1371/journal.pbio.3002446 (PMC10745200; doi:10.1371/journal.pbio.3002446)

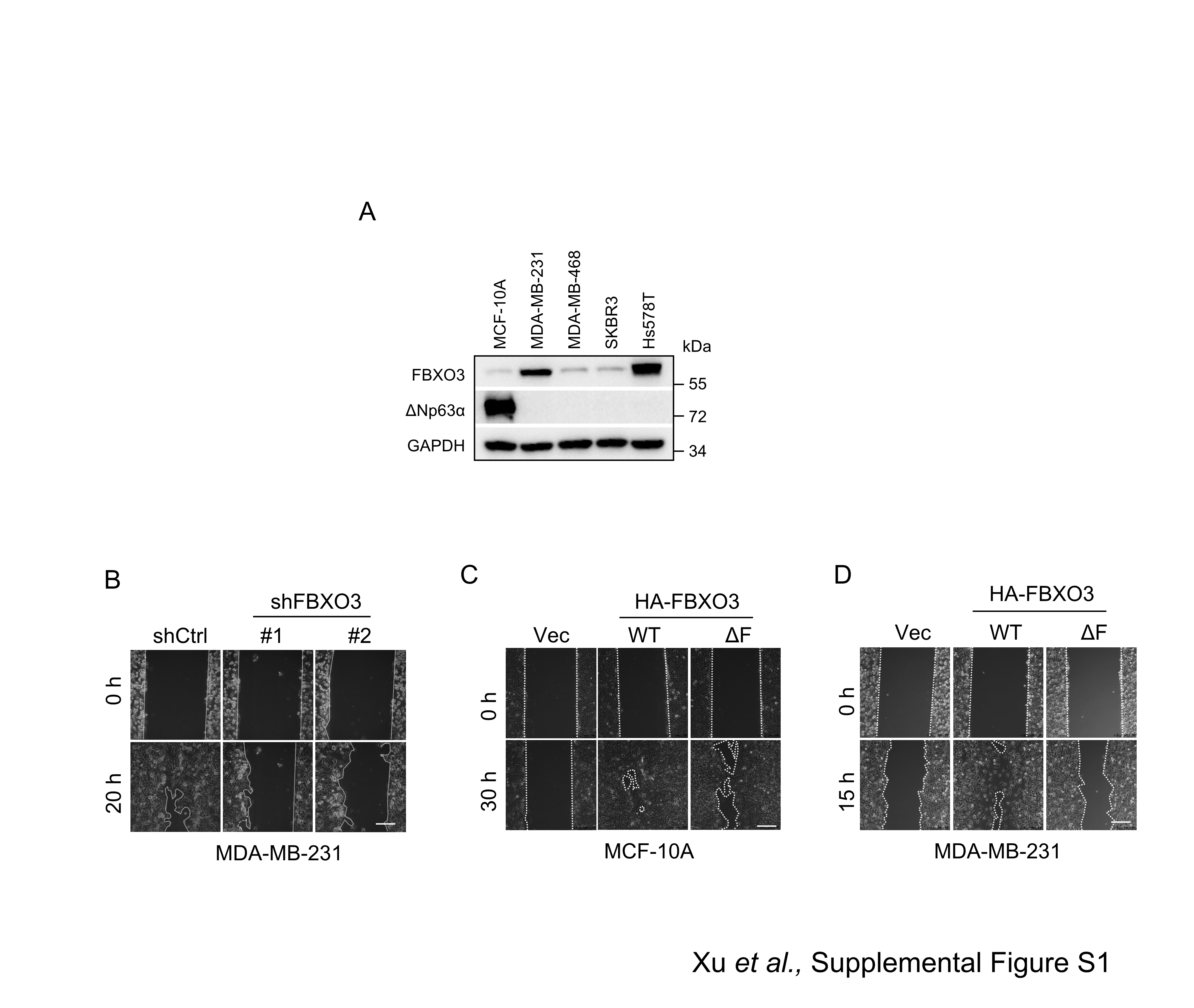

Supplement: S1 Fig — (A) MCF-10A, MDA-MB-231, MDA-MB-468, SKBR3, or Hs578T cells were subjected to western blot analysis. (B) MDA-MB-231 stable cells expressing specific shRNA against FBXO3 (shFBXO3-#1 or shFBXO3-#2) or GFP (shCtrl) were subjected to wound-healing assays. Scale bar = 100 μm. (C, D) MDA-MB-231 or MCF-10A stable cells expressing HA-FBXO3WT, HA-FBXO3ΔF, or a vector control (Vec) were subjected to wound-healing assays. Scale bar = 100 μm. (TIF) [file pbio.3002446.s003.tif]

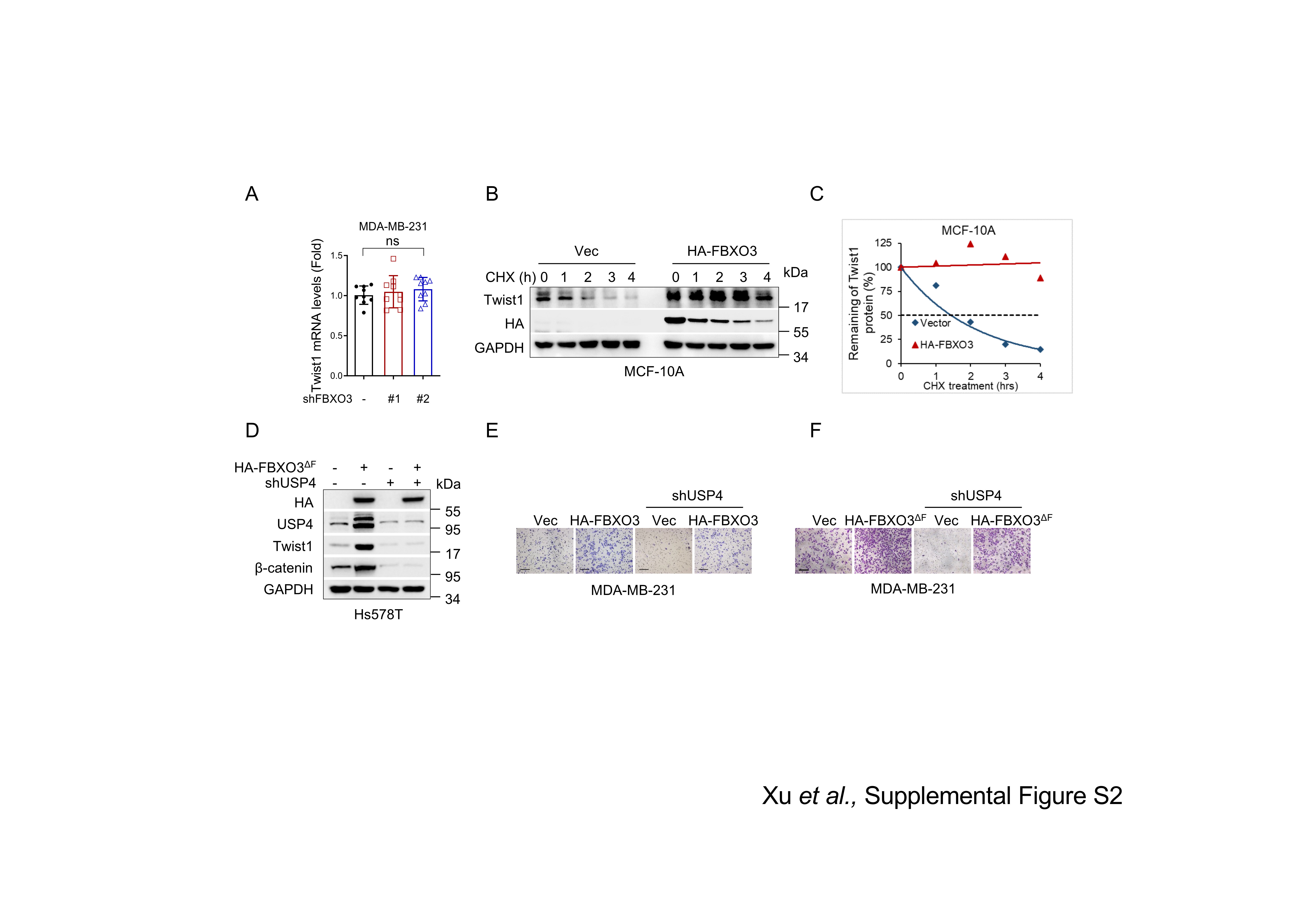

Supplement: S2 Fig — (A) MDA-MB-231 stable cells were subjected to qRT-PCR assays. Data were derived from 3 independent experiments. (B, C) MCF-10A stably expressing HA-FBXO3 was treated with cycloheximide (CHX) for the indicted times prior to western blot analyses (B). The relative Twist1 protein expression levels were quantitated by image J software (C). (D) Hs578T cells stably expressing HA-FBXO3ΔF were infected with a recombinant lentivirus expressing specific shRNA targeting to USP4 or a vector control (-) were subjected to western blot analyses. (E, F) MDA-MB-231 cells stably expressing HA-FBXO3WT, HA-FBXO3ΔF, or a vector control (Vec) were infected with a recombinant lentivirus expressing specific shRNA targeting to USP4 or GFP (-). Stable cells were then subjected to transwell assays. Scale bar = 100 μm. The data underlying the graphs shown in the figure can be found in S1 Data. (TIF) [file pbio.3002446.s004.tif]

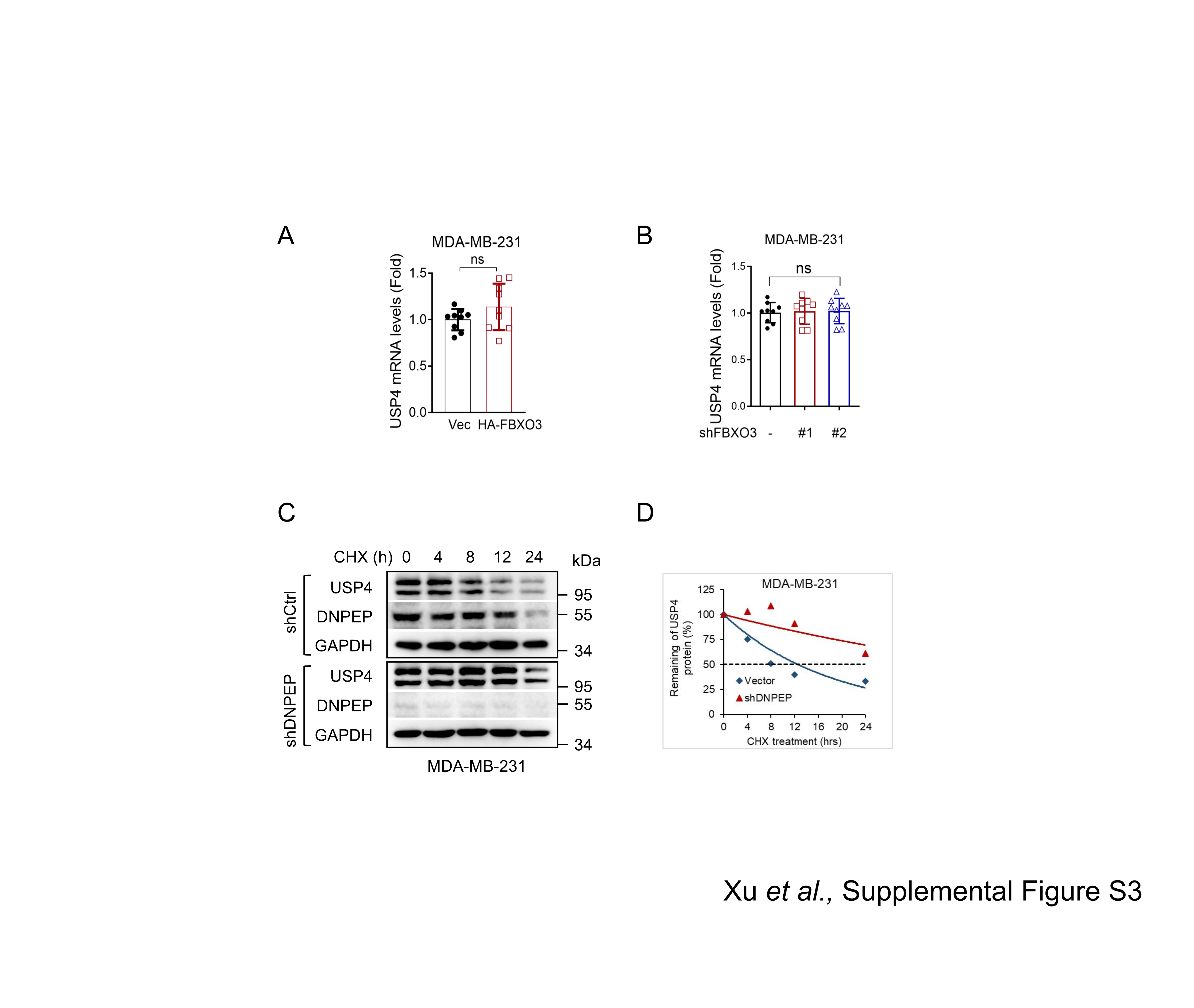

Supplement: S3 Fig — (A, B) MDA-MB-231 stable cells were subjected to qRT-PCR assays. Data were derived from 3 independent experiments. (C, D) MDA-MB-231 stable cells were treated with cycloheximide (CHX) for the indicted times prior to western blot analyses (C). The relative USP4 protein expression levels were quantitated by image J software (D). The data underlying the graphs shown in the figure can be found in S1 Data. (TIF) [file pbio.3002446.s005.tif]

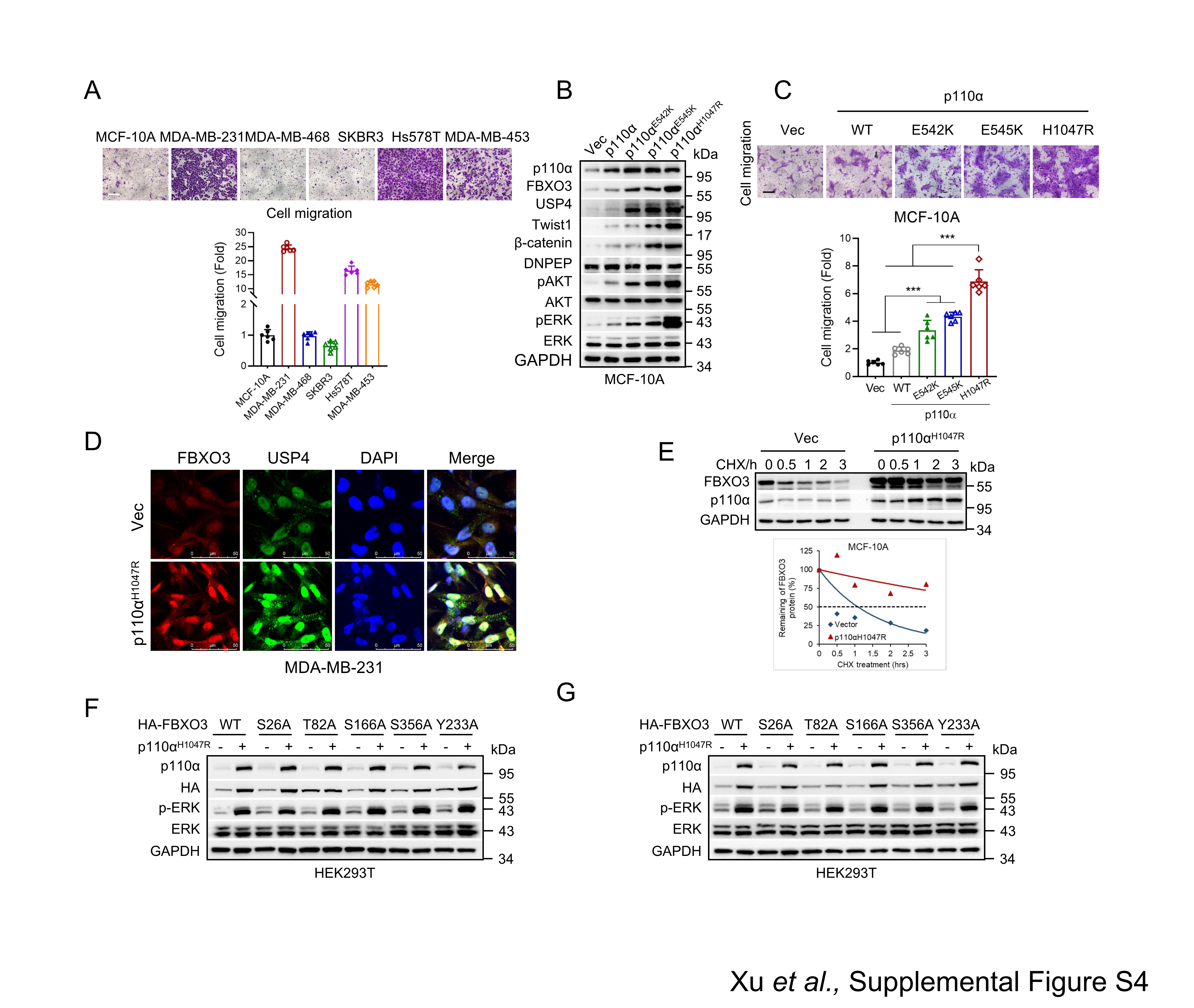

Supplement: S4 Fig — (A) MCF-10A, MDA-MB-231, MDA-MB-468, SKBR3, Hs578T, or MDA-MB-453 cells were subjected to transwell assays. Cells were suspended in serum-free media, seeded into the transwell inner chamber (5 × 10^4^ cells per chamber) and incubated for 24 h. Scale bar = 100 μm. (B, C) MCF-10A stably expressing p110α, p110αE542K, p110αE545K, p110αH1047R or a vector control (Vec) were subjected to western blot analyses (B) and transwell assays (C). Scale bar = 100 μm. (D) MDA-MB-231 cells stably expressing p110αH1047R or a vector control (Vec) were subjected to Immunofluorescence staining for USP4 (green) and FBXO3 (red) and counterstained with DAPI. Scale bar = 50 μm. (E) MCF-10A cells stably expressing p110αH1047R or a vector control (Vec) were treated with cycloheximide (CHX) for the indicted times, and the relative FBXO3 protein expression levels were quantified by image J software. (F, G) HEK293T cells were co-transfected with p110αH1047R and either HA-FBXO3 wild-type (WT) or an HA-FBXO3 mutant (S26A, T82A, S166A, S356A, or Y233A) expressing plasmids for 36 h, and cells were then subjected to western blot analyses. The data underlying the graphs shown in the figure can be found in S1 Data. (TIF) [file pbio.3002446.s006.tif]

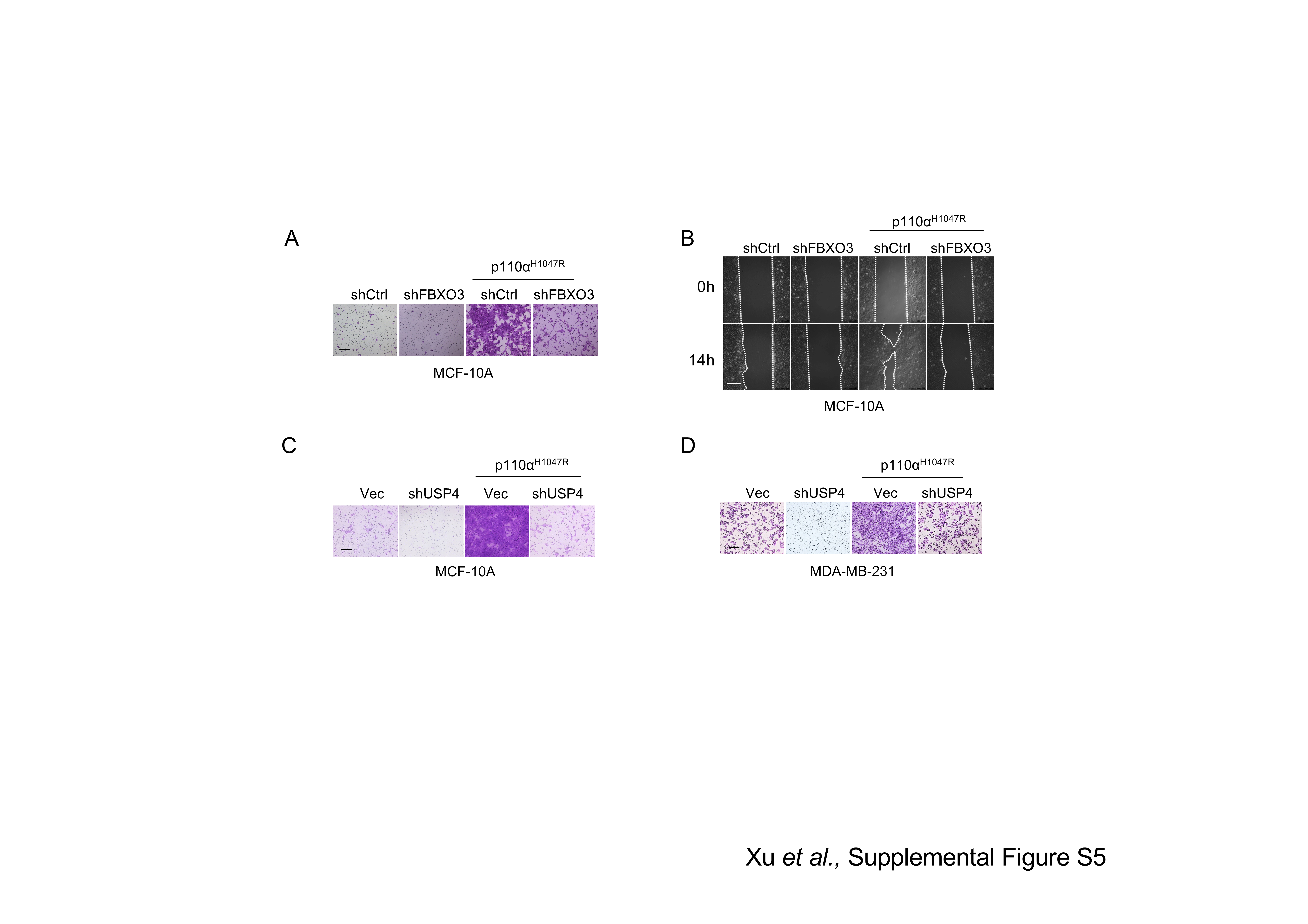

Supplement: S5 Fig — (A, B) MCF-10A cells stably expressing p110αH1047R were infected with a recombinant lentivirus expressing specific shRNA targeting to FBXO3 or GFP (shCtrl). Cells were then subjected to transwell assays (A) and wound-healing assays (B). Scale bar = 100 μm. (C, D) MDA-MB-231 or MCF-10A cells stably expressing p110αH1047R were infected with a recombinant lentivirus expressing specific shRNA targeting to USP4 or GFP (-). Cells were then subjected to transwell analyses. Scale bar = 100 μm. (TIF) [file pbio.3002446.s007.tif]
